# Deconstruction of rheumatoid arthritis synovium defines inflammatory subtypes

**DOI:** 10.1038/s41586-023-06708-y

**Published:** 2023-11-08

**Authors:** Fan Zhang, Anna Helena Jonsson, Aparna Nathan, Nghia Millard, Michelle Curtis, Qian Xiao, Maria Gutierrez-Arcelus, William Apruzzese, Gerald F. M. Watts, Dana Weisenfeld, Saba Nayar, Javier Rangel-Moreno, Nida Meednu, Kathryne E. Marks, Ian Mantel, Joyce B. Kang, Laurie Rumker, Joseph Mears, Kamil Slowikowski, Kathryn Weinand, Dana E. Orange, Laura Geraldino-Pardilla, Kevin D. Deane, Darren Tabechian, Arnoldas Ceponis, Gary S. Firestein, Mark Maybury, Ilfita Sahbudin, Ami Ben-Artzi, Arthur M. Mandelin, Alessandra Nerviani, Myles J. Lewis, Felice Rivellese, Costantino Pitzalis, Laura B. Hughes, Diane Horowitz, Edward DiCarlo, Ellen M. Gravallese, Brendan F. Boyce, Accelerating Medicines Partnership: RA/SLE Network, Jennifer Albrecht, Jennifer L. Barnas, Joan M. Bathon, David L. Boyle, S. Louis Bridges, Debbie Campbell, Hayley L. Carr, Adam Chicoine, Andrew Cordle, Patrick Dunn, Lindsy Forbess, Peter K. Gregersen, Joel M. Guthridge, Lionel B. Ivashkiv, Kazuyoshi Ishigaki, Judith A. James, Gregory Keras, Ilya Korsunsky, Amit Lakhanpal, James A. Lederer, Zhihan J. Li, Yuhong Li, Andrew McDavid, Mandy J. McGeachy, Karim Raza, Yakir Reshef, Christopher Ritchlin, William H. Robinson, Saori Sakaue, Jennifer A. Seifert, Anvita Singaraju, Melanie H. Smith, Dagmar Scheel-Toellner, Paul J. Utz, Michael H. Weisman, Aaron Wyse, Zhu Zhu, Larry W. Moreland, Susan M. Goodman, Harris Perlman, V. Michael Holers, Katherine P. Liao, Andrew Filer, Vivian P. Bykerk, Kevin Wei, Deepak A. Rao, Laura T. Donlin, Jennifer H. Anolik, Michael B. Brenner, Soumya Raychaudhuri

**Affiliations:** 1 Division of Rheumatology, Inflammation and Immunity, Department of Medicine, Brigham and Women’s Hospital and Harvard Medical School, Boston, MA USA; 2 Center for Data Sciences, Brigham and Women’s Hospital, Boston, MA USA; 3 Division of Genetics, Department of Medicine, Brigham and Women’s Hospital and Harvard Medical School, Boston, MA USA; 4 Department of Biomedical Informatics, Harvard Medical School, Boston, MA USA; 5 Broad Institute of MIT and Harvard, Cambridge, MA USA; 6 Division of Rheumatology and the Center for Health Artificial Intelligence, University of Colorado School of Medicine, Aurora, CO USA; 7 Division of Immunology, Department of Pediatrics, Boston Children’s Hospital and Harvard Medical School, Boston, MA USA; 8 Accelerating Medicines Partnership Program: Rheumatoid Arthritis and Systemic Lupus Erythematosus (AMP RA/SLE) Network, Bethesda, MD USA; 9 Rheumatology Research Group, Institute for Inflammation and Ageing, University of Birmingham, Birmingham, UK; 10 Birmingham Tissue Analytics, Institute of Translational Medicine, University of Birmingham, Birmingham, UK; 11 Division of Allergy, Immunology and Rheumatology, Department of Medicine, University of Rochester Medical Center, Rochester, NY USA; 12 Hospital for Special Surgery, New York, NY USA; 13 Weill Cornell Medicine, New York, NY USA; 14 Center for Immunology and Inflammatory Diseases, Department of Medicine, Massachusetts General Hospital (MGH), Boston, MA USA; 15 Laboratory of Molecular Neuro-Oncology, The Rockefeller University, New York, NY USA; 16 Division of Rheumatology, Columbia University College of Physicians and Surgeons, New York, NY USA; 17 Division of Rheumatology, University of Colorado School of Medicine, Aurora, CO USA; 18 Division of Rheumatology, Allergy and Immunology, University of California, San Diego, La Jolla, CA USA; 19 NIHR Birmingham Biomedical Research Center and Clinical Research Facility, University of Birmingham, Queen Elizabeth Hospital, Birmingham, UK; 20 Division of Rheumatology, Cedars-Sinai Medical Center, Los Angeles, CA USA; 21 Division of Rheumatology, Department of Medicine, Northwestern University Feinberg School of Medicine, Chicago, IL USA; 22 Centre for Experimental Medicine and Rheumatology, EULAR Centre of Excellence, William Harvey Research Institute, Queen Mary University of London, London, UK; 23 Barts Health NHS Trust, Barts Biomedical Research Centre (BRC), National Institute for Health and Care Research (NIHR), London, UK; 24 Department of Biomedical Sciences, Humanitas University and Humanitas Research Hospital, Milan, Italy; 25 Division of Clinical Immunology and Rheumatology, Department of Medicine, University of Alabama at Birmingham, Birmingham, AL USA; 26 Feinstein Institute for Medical Research, Northwell Health, Manhasset, New York, NY USA; 27 Department of Pathology and Laboratory Medicine, Hospital for Special Surgery, New York, NY USA; 28 Department of Pathology and Laboratory Medicine, University of Rochester Medical Center, Rochester, NY USA; 29 Division of Rheumatology and Clinical Immunology, University of Pittsburgh School of Medicine, Pittsburgh, PA USA; 38 Present Address: Division of Rheumatology, University of Colorado School of Medicine, Aurora, CO USA; 30 Department of Radiology, University of Pittsburgh Medical Center, Pittsburgh, PA USA; 31 Division of Allergy, Immunology and Transplantation, National Institute of Allergy and Infectious Diseases, National Institutes of Health, Bethesda, MD USA; 32 Northrop Grumman Health Solutions, Rockville, MD USA; 33 Department of Arthritis and Clinical Immunology, Oklahoma Medical Research Foundation, Oklahoma City, OK USA; 34 Laboratory for Human Immunogenetics, RIKEN Center for Integrative Medical Sciences, Yokohama, Japan; 35 Department of Surgery, Brigham and Women’s Hospital and Harvard Medical School, Boston, MA USA; 36 Department of Biostatistics and Computational Biology, University of Rochester School of Medicine and Dentistry, Rochester, NY USA; 37 Division of Immunology and Rheumatology, Institute for Immunity, Transplantation and Infection, Stanford University School of Medicine, Stanford, CA USA

**Keywords:** Translational immunology, Rheumatoid arthritis

## Abstract

Rheumatoid arthritis is a prototypical autoimmune disease that causes joint inflammation and destruction^1^. There is currently no cure for rheumatoid arthritis, and the effectiveness of treatments varies across patients, suggesting an undefined pathogenic diversity^1,2^. Here, to deconstruct the cell states and pathways that characterize this pathogenic heterogeneity, we profiled the full spectrum of cells in inflamed synovium from patients with rheumatoid arthritis. We used multi-modal single-cell RNA-sequencing and surface protein data coupled with histology of synovial tissue from 79 donors to build single-cell atlas of rheumatoid arthritis synovial tissue that includes more than 314,000 cells. We stratified tissues into six groups, referred to as cell-type abundance phenotypes (CTAPs), each characterized by selectively enriched cell states. These CTAPs demonstrate the diversity of synovial inflammation in rheumatoid arthritis, ranging from samples enriched for T and B cells to those largely lacking lymphocytes. Disease-relevant cell states, cytokines, risk genes, histology and serology metrics are associated with particular CTAPs. CTAPs are dynamic and can predict treatment response, highlighting the clinical utility of classifying rheumatoid arthritis synovial phenotypes. This comprehensive atlas and molecular, tissue-based stratification of rheumatoid arthritis synovial tissue reveal new insights into rheumatoid arthritis pathology and heterogeneity that could inform novel targeted treatments.

## Abstract (web summary)

Single-cell transcriptomic and proteomic data from synovial tissue from individuals with rheumatoid arthritis classify patients into groups based on abundance of cell states that can provide insights into pathology and predict individual treatment responses.

## Main

Rheumatoid arthritis is a systemic autoimmune disease that affects up to 1% of the population^3^. It is characterized by inflammation of synovial joint tissue and extra-articular manifestations that lead to pain, joint damage and disability^1^. The clinical course of rheumatoid arthritis has been transformed by targeted therapies, including those aimed at TNF, IL-6, B cells, T cell co-stimulation and the JAK–STAT pathway^1^. However, many patients are refractory to these therapies and do not achieve remission^2^. Thus, there is a clinical need for new treatment targets and for predictors of patient-specific responses to treatment. Genetic diversity and variable responses to targeted therapies suggest that rheumatoid arthritis is a heterogeneous disease^4^. However, genetic and clinical differences in disease duration or activity do not reliably predict the treatment response or druggable targets^1,5^.

A more granular understanding of cell states and synovial phenotypes in inflamed joints could inform prognosis and therapeutic targets. Encouragingly, clinical trials using histologic or bulk RNA-sequencing (RNA-seq) analysis of synovial tissue suggest that treatment response may depend on synovial cellular composition^6,7^. Previous studies have identified effector cell states in rheumatoid arthritis pathophysiology that represent promising treatment targets, including *HBEGF*^*+*^*IL1B*^*+*^ macrophages, SLAMF7^+^ super-activated macrophages, *MERTK*^*+*^ macrophages, CD11c^*+*^ autoimmune-associated B cells (ABCs), PD-1^hi^ T peripheral helper (T_PH_) cells, granzyme K^+^CD8^+^ T cells and *NOTCH3*^*+*^ synovial fibroblasts^8–16^. To determine whether some states are enriched only in specific subsets of patients, we analysed cell-state composition in a clinically diverse set of patients with active rheumatoid arthritis. As rheumatoid arthritis shares disease-associated tissue cell states and genetic risk loci with other autoimmune diseases^17,18^, these analyses may offer insights into other diseases that feature tissue inflammation.

## Recruitment and multi-modal analysis of tissue

**Fig. 1:**
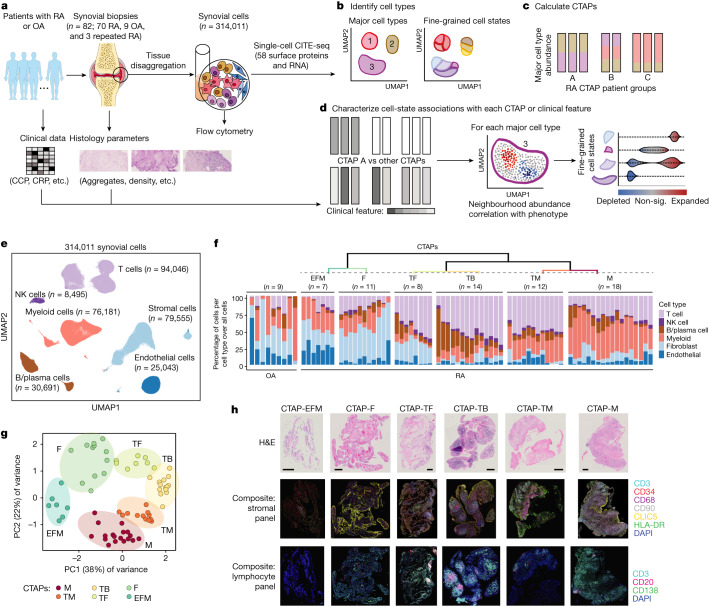
Overview of the multi-modal single-cell synovial tissue pipeline and cell-type abundance analysis that reveals distinct rheumatoid arthritis CTAPs. **a**–**d**, Description (**a**) of the patient recruitment, clinical and histologic metrics, synovial sample processing pipeline and computational analysis strategy, including identification of major cell types and fine-grained cell states (**b**), definition of distinct rheumatoid arthritis CTAPs (**c**), and cell neighbourhood associations with each CTAP or with clinical or histologic parameters for each major cell type (**d**). OA, osteoarthritis; RA, rheumatoid arthritis; sig., significant. **e**, Integrative uniform manifold approximation and projection (UMAP) based on mRNA and protein discriminated major cell types, **f**, Hierarchical clustering of cell-type abundances captures six rheumatoid arthritis subgroups, referred to as CTAPs. The nine osteoarthritis samples are shown as a comparison. Each bar represents one synovial sample, coloured by the proportion of each major cell type. **g**, PCA of major cell-type abundances. Each dot represents a sample, plotted based on its PC1 and PC2 projections and coloured by CTAPs. **h**, Representative synovial tissue fragments from each of the CTAPs. Top row, haematoxylin and eosin (H&E) staining. Middle row, immunofluorescence microscopy for CD3, CD34, CD68, CD90, CLIC5 and HLA-DR. Bottom row, immunofluorescence microscopy for CD3, CD20 and CD138. Scale bars: 100 μm (CTAP-EFM) and 250 μm (all other images). Single-colour images are presented in Supplementary Fig. 4. A total of 150 fragments from 36 donors were stained in batches and analysed as a single cohort. Parts of Fig. 1a were generated using Servier Medical Art, provided by Servier, licensed under a Creative Commons Attribution 3.0 unported license.

We obtained a total of 82 synovial tissue samples from patients exhibiting moderate to high disease activity (clinical disease activity index (CDAI) ≥ 10). To capture a clinical spectrum of rheumatoid arthritis, we collected biopsies from treatment-naive patients (*n* = 28) early in their disease course, methotrexate (MTX)-inadequate responders (*n* = 27), and anti-TNF agent-inadequate responders (*n* = 15) as well as from patients with osteoarthritis (*n* = 9) (Fig. 1a–d, Supplementary Table 1).

**Extended Data Fig. 1:**
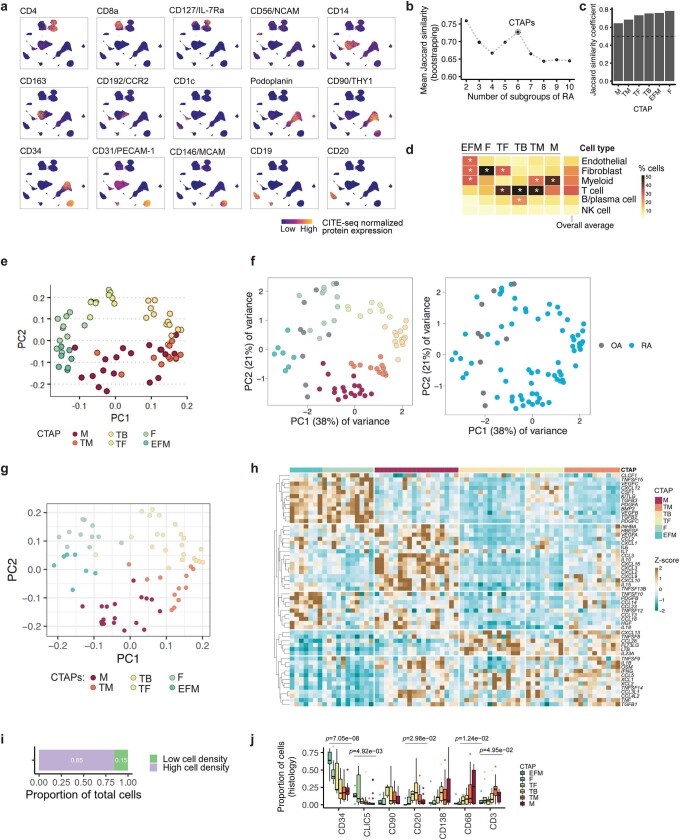
Robust CTAP definition and quantitative cellular histology analysis. **a**, UMAPs of CITE-seq antibody-based expression of cell-type lineage protein markers. Cells are colored based on expression from blue (low) to yellow (high). **b**, Mean Jaccard similarity coefficient to test CTAP stability by bootstrapping 10,000 times for each tested number of patient subgroups ranging from 2 to 10. **c**, Mean Jaccard similarity coefficient for each CTAP, comparing full clustering and 10,000 bootstrapped datasets. **d**, Average proportions of each major cell type among samples in each CTAP. Overall average proportions across all the samples are shown as a comparator. Asterisk represents the proportion that is greater than the overall average for that cell type, **e**, PCA of samples based on cell-type abundances, adjusting for disease duration and treatment. Each dot represents a sample, plotted based on its PC1 and PC2 projections and colored by CTAPs. **f**, Projection of OA samples onto PCA of samples based on cell-type abundances from Fig. 1j. OA samples are marked with gray points; RA samples are colored based on CTAP (left) or in blue (right). **g**, PCA of samples based on pseudo-bulk gene expression of 55 soluble immune mediators. Each dot represents a sample, plotted based on its PC1 and PC2 projections and colored by CTAPs. **h**, Heatmap of pseudo-bulk gene expression of soluble immune mediators across samples, grouped by CTAP. Boxes are colored based on the gene’s scaled pseudo-bulk expression across samples. **i**, Bar graph of the proportion of total cells located in high-density and low-density fragments, as captured by histology imaging. Quantitation of total cellular composition demonstrated that fragments with highest cell density (top 50%) contained 86% of total cells and are therefore likely the primary drivers of CTAP classification. **j**, Box plots of the proportion of cells in high-density fragments (N = 76) expressing each marker in histology imaging, stratified by CTAP. Points represent outlier samples (> 1.5 * IQR from median). Box plots show median (vertical bar), 25th and 75th percentiles (lower and upper bounds of the box, respectively) and 1.5 x IQR (or minimum/maximum values; end of whiskers). P-values are calculated with one-way ANOVA tests with Bonferroni correction.

We simultaneously characterized the transcriptome and surface expression of 58 proteins (Supplementary Table 2) in a total of 314,011 cells (more than 3,800 cells per sample) after quality control (Supplementary Fig. 1). We integrated surface marker and RNA data using canonical correlation analysis, corrected batch effects and defined six major cell types: T, B and plasma (B/plasma), natural killer (NK), myeloid, stromal and endothelial cells (Fig. 1e, Extended Data Fig. 1a, Supplementary Fig. 2 and Supplementary Table 3).

## Stratifying synovium by cell-type abundance

To define potentially distinct tissue inflammatory phenotypes, we hierarchically clustered synovial samples on the basis of the frequency of the six major cell lineages (Fig. 1f,g). On the basis of in-group similarity with bootstrapping, we arrived at six different categories that we call CTAPs, which are largely robust to adjustment for treatment and disease duration (Extended Data Fig. 1b–e). We named the CTAPs on the basis of relatively enriched cell type(s): (1) endothelial, fibroblast and myeloid cells (EFM); (2) fibroblasts (F); (3) T cells and fibroblasts (TF); (4) T and B cells (TB); (5) T and myeloid cells (TM); and (6) myeloid cells (M) (Extended Data Fig. 1d and Supplementary Table 4). Alternative clustering schemes using highly variable genes, all transcriptional states, or separating plasma cells from non-plasma B cells led to similar results (Supplementary Fig. 3). Post hoc mapping of the osteoarthritis samples demonstrates that they most resemble CTAP-EFM and CTAP-F (Extended Data Fig. 1f). Categorization by effector functions using pseudo-bulk expression of 55 cytokines, chemokines and growth factors was similar to the cell lineage-based CTAP categorization (Extended Data Fig. 1g,h).

## CTAP patterns are consistent across fragments

To examine the robustness of CTAPs across paired biopsy fragments from the same joint, we performed immunofluorescence microscopy staining on synovial tissue fragments from a subset of patients (*n* = 36) (Fig. 1h and Supplementary Fig. 4). We compared cell-type proportions in individual high-density biopsy fragments with the disaggregated cellular indexing of transcriptomes and epitopes (CITE-seq)-based cell frequencies (Extended Data Fig. 1i,j). The proportions of cell types followed the patterns predicted by the CITE-seq-based CTAP assignment. For example, CD20^+^ (that is, non-plasma) B cells were most frequent in CTAP-TB, whereas CD68^+^ myeloid cells were most frequent in CTAP-M and CTAP-TM. As the histology analysis was performed on synovial tissue fragments separate from those used for CITE-seq, these findings support the consistency of CTAP assignments across a joint.

## A rheumatoid arthritis synovial cell-state atlas

**Fig. 2:**
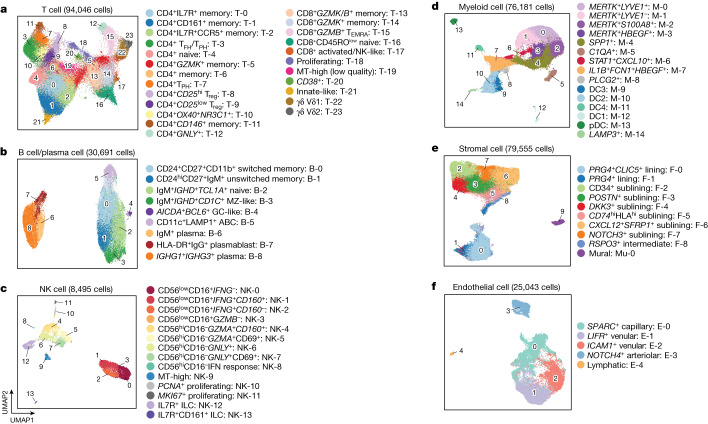
Cell-type-specific single-cell analysis captures 77 distinct cell states in rheumatoid arthritis synovium. **a**–**f**, Cell-type-specific reference UMAPs for T cells (**a**) B/plasma cells (**b**), NK cells (**c**), myeloid cells (**d**), stromal cells (**e**) and endothelial cells (**f**), coloured by fine-grained cell-state clusters. MT, mitochondrial; MZ, marginal zone; pDC, plasmacytoid dendritic cell.

**Extended Data Fig. 2:**
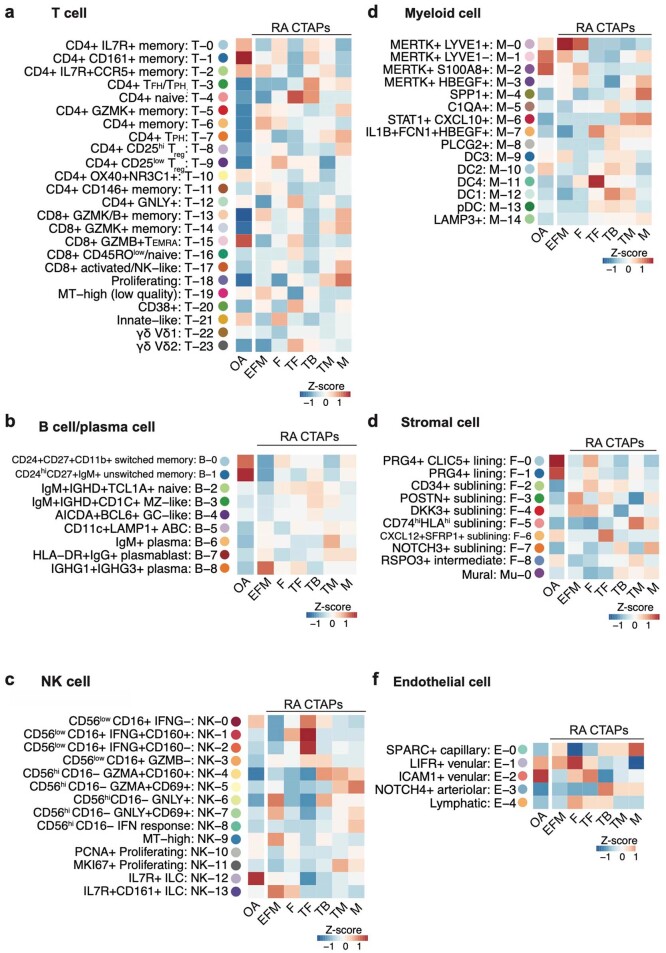
Relative enrichment of fine-grain cell clusters across CTAPs and OA. **a**-**f**, Heatmaps show the average proportions of each cluster in the given cell type across patient samples in each RA CTAP and OA, scaled within each cluster.

We defined finer-grained cell states and quantified cluster abundances within cell types (Fig. 2 and Extended Data Fig. 2) using canonical variates from canonical correlation analysis reflecting both RNA and protein for T and B cells and mRNA principal components for myeloid, stromal and endothelial cell states (Supplementary Figs. 5 and 6 and Supplementary Table 3). In total we defined 77 cell states: 24 T cell clusters (*n* = 94,046 cells), 9 B/plasma cell clusters (*n* = 30,691), 14 NK clusters (*n* = 8,495), 15 myeloid clusters (*n* = 76,181), 5 endothelial clusters (*n* = 25,043) and 10 stromal clusters (*n* = 79,555) (Fig. 2 and Supplementary Table 5). Cell states associated with rheumatoid arthritis versus osteoarthritis in a previous study of more than 5,000 synovial cells were also associated with rheumatoid arthritis in this dataset (Supplementary Fig. 7 and Supplementary Table 6).

**Extended Data Fig. 3:**
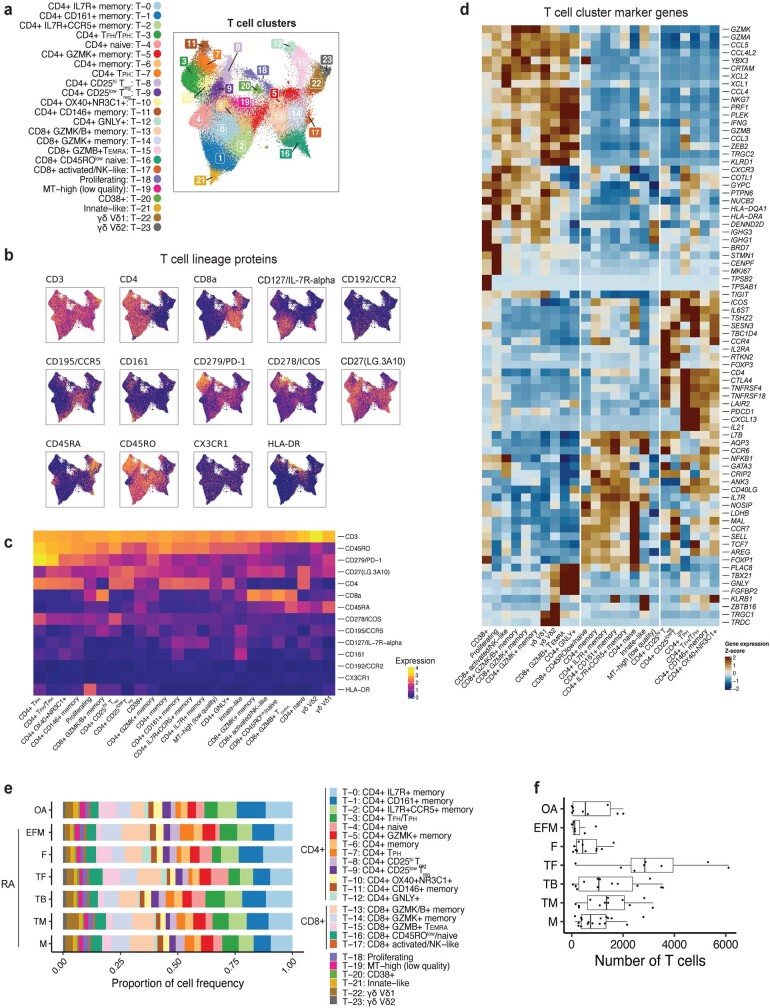
T cell-specific analysis. **a**, T cell UMAP colored by fine-grained cell-state clusters, **b**, Expression of selected surface proteins among T cells. Cells are colored from blue (low) to yellow (high), **c**, Heatmap of surface protein expression in T cell clusters colored according to the average normalized expression across cells in the cluster, **d**, Heatmap of gene expression in T cell clusters colored according to the average normalized expression across cells in the cluster, scaled for each gene across clusters, **e**, Distribution of T cells across clusters, stratified by CTAP. The size of each segment of each bar corresponds to the average proportion of cells in that cluster across donors from that CTAP. **f**, Number of T cells per individual, stratified by CTAP. Points represent individuals (N = 82); OA (N = 9), (EFM (N = 7), F (N = 11), TF (N = 8), TB (N = 14), TM (n = 12), M (N = 18). Box plots show median (vertical bar), 25th and 75th percentiles (lower and upper bounds of the box, respectively) and 1.5 x IQR (or minimum/maximum values; end of whiskers).

The 24 T cell clusters spanned innate-like states and CD4^+^ and CD8^+^ adaptive lineages, including states implicated in autoimmunity, such as regulatory CD4^+^ T cells (T_reg_) (T-8 and T-9) and *CXCL13*- and *IL21*-expressing T follicular helper (T_FH_) and T_PH_ cells^17,19^ (T-3 and T-7) (Fig. 2a and Extended Data Figs. 2 and 3). T-7 exclusively comprised T_PH_ cells and expressed more *ICOS*, *IFNG* and *GZMA*, whereas T-3 contained T_FH_ and T_PH_ (T_FH_/T_PH_) cells expressing the lymphoid homing marker gene *CCR7*. CD8^+^ subsets expressed different combinations of *GZMB* and *GZMK*, reflecting differential cytotoxic potential. Using cell surface protein data, we resolved T cell clusters that were not observed in our earlier study^8^, including CD4^+^*GNLY*^+^ (T-12), double-negative (CD4^−^CD8^−^) γδ T cells expressing *TRDC* (T-22 and T-23) and double-negative and CD8^+^ T cells expressing *ZBTB16* (which encodes PLZF) that resemble NK T cells and mucosal-associated innate T (MAIT) cells (T-21).

**Extended Data Fig. 4:**
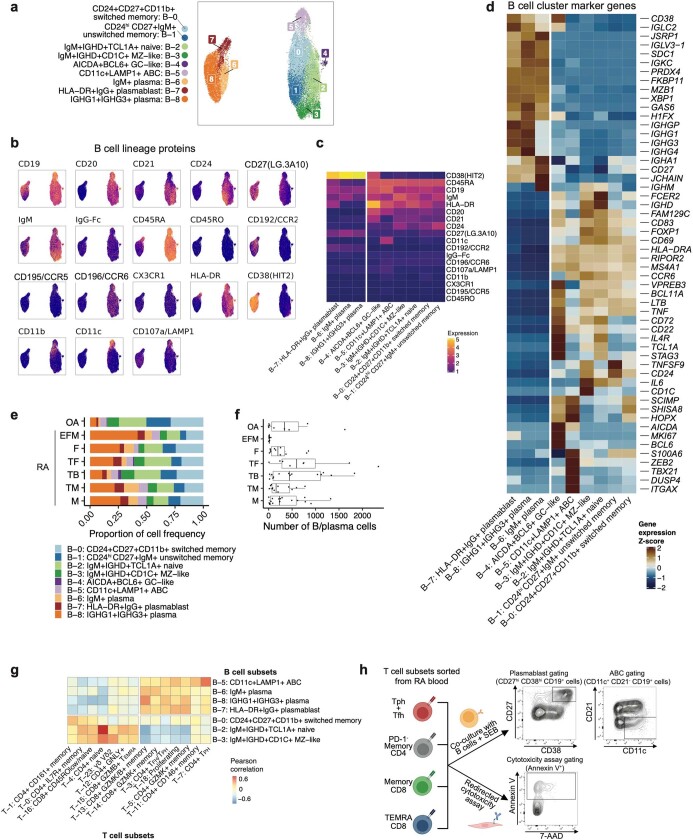
B/plasma cell-specific analysis. **a**, B/plasma cell UMAP colored by fine-grained cell state clusters, **b** Expression of selected surface proteins among B/plasma cells. Cells are colored from blue (low) to yellow (high), **c**, Heatmap of surface protein expression in B/plasma cell clusters colored according to the average normalized expression across cells in the cluster, **d**, Heatmap of gene expression in B/plasma cell clusters colored according to the average normalized expression across cells in the cluster, scaled for each gene across clusters, **e**, Distribution of B/plasma cells across clusters, stratified by CTAP. The size of each segment of each bar corresponds to the average proportion of cells in that cluster across donors from that CTAP. **f**, Number of B/plasma cells per individual, stratified by CTAP. Points represent individuals (N = 82); OA (N = 9), EFM (N = 7), F (N = 11), TF (N = 8), TB (N = 14), TM (N = 12), M (N = 18). Box plots show median (vertical bar), 25th and 75th percentiles (lower and upper bounds of the box, respectively) and 1.5 x IQR (or minimum/maximum values; end of whiskers). **g**, Heatmap of correlations between select T and B cell subsets, colored by Pearson correlation between per-donor proportions. **h**, Schematic representation of the experimental design of the T cell functional assays and representative flow cytometry plots showing gating of plasmablasts (CD27^hi^ CD38^hi^ CD19^+^ cells), ABC B cells (CD11c^+^ CD21^−^ CD19^+^ cells) and dead target cells (Annexin V^+^). Parts of this schematic were created using BioRender.

CD20 (encoded by *MS4A1*)-expressing B cells comprised six clusters, including IgM^+^*IGHD*^+^*TCL1A*^+^ naive (B-2), CD24^hi^CD27^+^IgM^+^ unswitched memory (B-1) and CD24^*+*^CD27^*+*^CD11b^*+*^ (CD11b is also known as ITGAM) switched memory (B-0) B cells (Fig. 2b and Extended Data Figs. 2 and 4). CD11c^+^*CXCR5*^low^ (CD11c is also known as *ITGAX*) ABCs (B-5) expressed LAMP1, HLA-DR and *CIITA*, indicating B cell antigen presentation^20–22^. Unexpectedly, we observed *CD1C*^+^ B cells (B-3) with CD27 and *IGHD* expression, consistent with recirculating extrasplenic marginal zone B cells. These and other non-plasma B cells expressed *IL6* and *TNF* (Extended Data Fig. 4d). We identified *AICDA*^+^*BCL6*^+^ germinal centre-like B cells (B-4), consistent with ectopic germinal centre formation in synovium^23^. Plasma cell populations included HLA-DR^+^IgG^+^ plasmablasts (B-7) expressing *MKI67*, IgM^+^ plasma cells (B-6) and mature *IGHG1*^+^*IGHG3*^+^ plasma cells (B-8), possibly reflecting both in situ generation and recruitment from the circulation.

**Extended Data Fig. 5:**
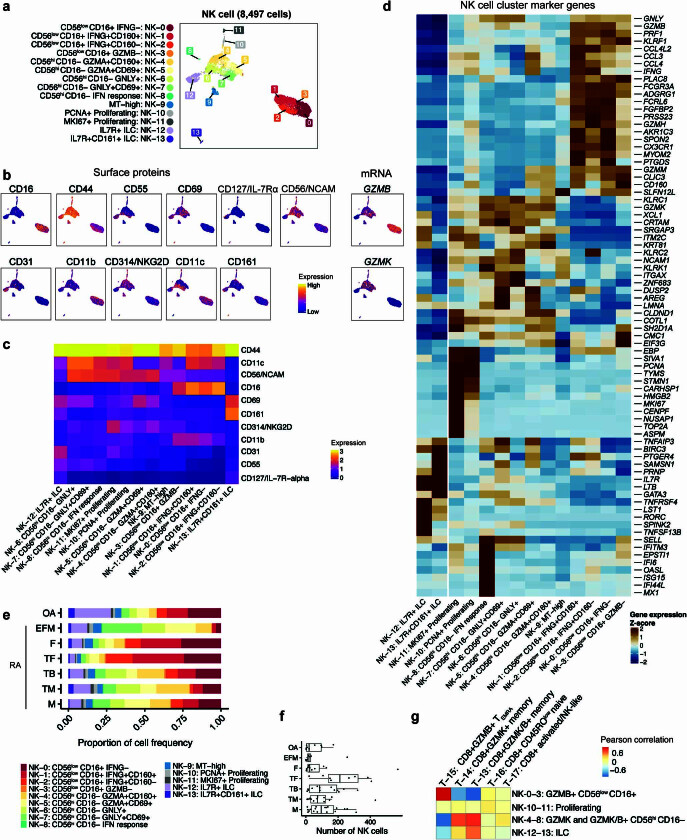
NK cell-specific analysis. **a**, NK cell UMAP colored by fine-grained cell state clusters, **b**, Expression of selected surface proteins or mRNA transcripts among NK cells colored from blue (low) to yellow (high), **c**, Heatmap of surface protein expression in NK cell clusters colored according to the average normalized expression across cells in the cluster, **d**, Heatmap of gene expression in NK cell clusters colored according to the average normalized expression across cells in the cluster, scaled for each gene across clusters, **e**, Distribution of NK cells across clusters, stratified by CTAP. The size of each segment of each bar corresponds to the average proportion of cells in that cluster across donors from that CTAP. **f**, Number of NK cells per individual, stratified by CTAP. Points represent individuals (N = 82); OA (N = 9), EFM (N = 7), F (N = 11), TF (N = 8), TB (N = 14), TM (N = 12), M (N = 18). Box plots show median (vertical bar), 25th and 75th percentiles (lower and upper bounds of the box, respectively) and 1.5 x IQR (or minimum/maximum values; end of whiskers). **g**, Heatmap colored by Pearson correlation between per-donor CD8^+^ T cell and NK cell cluster abundances.

We also captured innate lymphocytes, including CD56^hi^CD16^−^ NK (eight clusters), CD56^low^CD16^+^ NK (four clusters) and CD56^low^CD16^−^IL7R^+^ innate lymphoid cells (ILCs) (two clusters) (Fig. 2c and Extended Data Figs. 2 and 5). CD56^hi^CD16^−^ NK cells were more abundant (mean 48% per donor) than CD56^low^CD16^+^ NK cells (36%) and ILCs (13%). CD56^hi^CD16^−^ NK clusters expressed *GZMK*, with variable expression of cytotoxicity genes such as *GZMB* and *GNLY*. CD56^low^CD16^+^ NK cells exhibited universally high expression of *GZMB*, *GNLY* and *PRF1*. Several NK cell clusters highly expressed *IFNG* (Extended Data Fig. 5d). ILCs, identified by the absence of CD56 and CD16 with high CD127 (also known as IL-7Rα) protein, included group 3 ILCs (*RORC*^*+*^ NK-12) and group 2 ILCs^24^ (CD161^+^*GATA3*^*+*^ NK-13).

**Extended Data Fig. 6:**
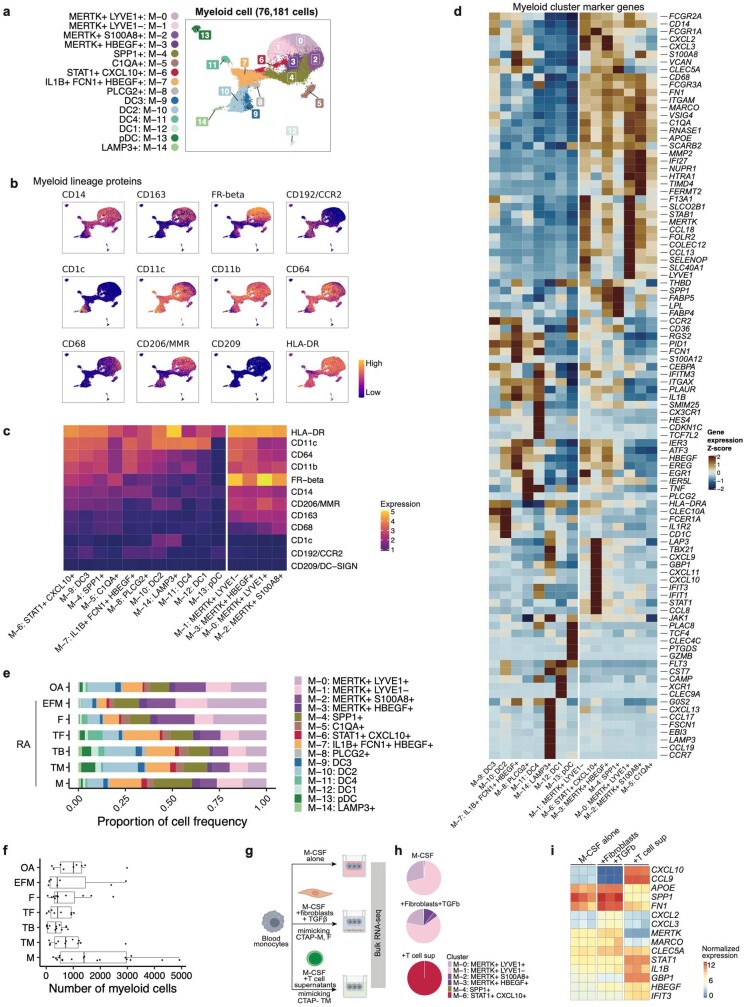
Myeloid cell-specific analysis. **a**, Myeloid cell UMAP colored by fine-grained cell state clusters, **b**, Expression of selected surface proteins among myeloid cells colored from blue (low) to yellow (high), **c**, Heatmap of surface protein expression in myeloid cell clusters colored according to the average normalized expression across cells in the cluster, **d**, Heatmap of gene expression in myeloid cell clusters colored according to the average normalized expression across cells in the cluster, scaled for each gene across clusters, **e**, Distribution of myeloid cells across clusters, stratified by CTAP. The size of each segment of each bar corresponds to the average proportion of cells in that cluster across donors from that CTAP. **f**, Number of myeloid cells per individual, stratified by CTAP. Points represent individuals (N = 82); OA (N = 9), EFM (N = 7), F (N = 11), TF (N = 8), TB (N = 14), TM (N = 12), M (N = 18). Box plots show median (vertical bar), 25th and 75th percentiles (lower and upper bounds of the box, respectively) and 1.5 x IQR (or minimum/maximum values; end of whiskers). **g**, Schematic representation of the experimental design of the myeloid cell assays. Parts of this schematic were created using BioRender. **h**, Linear discriminant analysis classification of bulk RNA-seq obtained from myeloid cells cultured in the indicated conditions. Each condition was performed with three biological replicates, and cluster proportions in each pie chart were calculated from the mean of the posterior probability values across replicates. **i**, Heatmap showing expression of selected CTAP-relevant genes in bulk RNA-seq of blood monocytes cultured in the indicated conditions. Columns correspond to three biological replicates for each condition, and boxes are colored by normalized gene expression.

We identified 15 myeloid clusters (Fig. 2d). CD68 and CCR2 discriminated tissue macrophages from infiltrating monocytes (Extended Data Figs. 2 and 6). Three tissue macrophage clusters (M-0, M-1 and M-2) were abundant in both osteoarthritis and rheumatoid arthritis synovium and expressed the phagocytic factors CD206 (also known as macrophage mannose receptor (MMR)) and CD163 and *MERTK* (Extended Data Fig. 6b–d), suggesting a homeostatic debris-clearing function^25,26^. *LYVE1* expression (M-0) is likely to indicate a perivascular function^12,27^. Infiltrating monocytes included a previously described *IL1B*^*+*^*FCN1*^*+*^*HBEGF*^*+*^ pro-inflammatory subset (M-7), probably derived from classical CD14^hi^ monocytes^8,12^ and a *STAT1*^*+*^*CXCL10*^*+*^ subset (M-6) that expresses interferon-response genes. *MERTK*^+^*HBEGF*^+^ (M-3) and *SPP1*^+^ (M-4) subsets expressed *SPP1* (osteopontin) and other factors consistent with wound-healing responses^28,29^. Four dendritic cell (DC) populations corresponded to subsets described by Villani et al.^30^. *CLEC10A*^hi^ DC2 and DC3 (M-9 and M-10) and *CLEC9A*^+^*THBD*^+^ DC1 (M-12) are likely to activate CD4^+^ and CD8^+^ T cells, respectively, whereas DC4 (M-11) expressed CD16^+^ monocyte factors and an interferon signature (Extended Data Fig. 6d). A fifth DC subset (M-14) highly expressed the endosomal marker *LAMP3*^31^.

**Extended Data Fig. 7:**
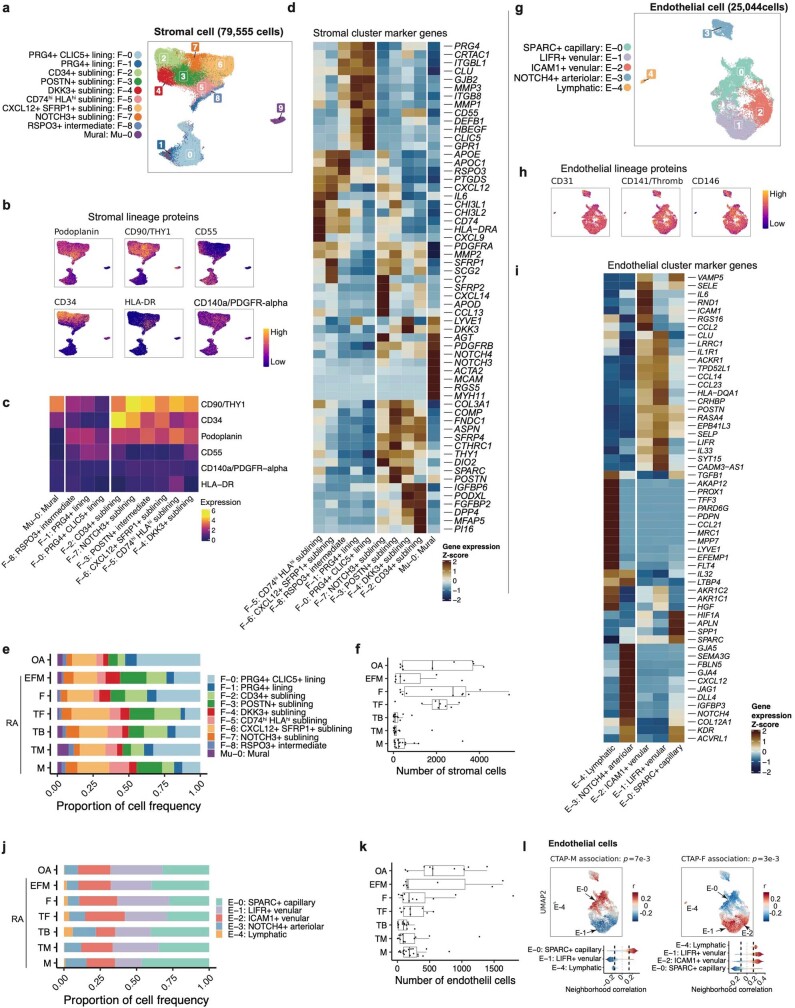
Stromal- and endothelial-specific analysis. **a**, Stromal cell UMAP colored by fine-grained cell state clusters, **b**, Expression of selected surface proteins among stromal cells colored from blue (low) to yellow (high), **c**, Heatmap of surface protein expression in stromal cell clusters colored according to the average normalized expression across cells in the cluster, **d**, Heatmap of gene expression in stromal cell clusters colored according to the average normalized expression across cells in the cluster, scaled for each gene across clusters, **e**, Distribution of stromal cells across clusters, stratified by CTAP. The size of each segment of each bar corresponds to the average proportion of cells in that cluster across donors from that CTAP, **f**, Number of stromal cells per individual, stratified by CTAP. Points represent individuals (N = 82); OA (N = 9), (EFM (N = 7), F (N = 11), TF (N = 8), TB (N = 14), TM (N = 12), M (N = 18). Box plots show median (vertical bar), 25th and 75th percentiles (lower and upper bounds of the box, respectively) and 1.5 x IQR (or minimum/maximum values; end of whiskers), **g**, Endothelial cell UMAP colored by fine-grained cell state clusters, **h**, Expression of selected surface proteins among endothelial cells colored from blue (low) to yellow (high), **i**, Heatmap of gene expression in endothelial cell clusters colored according to the average normalized expression across cells in the cluster, scaled for each gene across clusters, **j**, Distribution of endothelial cells across clusters, stratified by CTAP. The size of each segment of each bar corresponds to the average proportion of cells in that cluster across donors from that CTAP. **k**, Number of endothelial cells per individual, stratified by CTAP. Points represent individuals (N = 82); OA (N = 9), EFM (N = 7), F (N = 11), TF (N = 8), TB (N = 14), TM (n = 12), M (N = 18). Box plots show median (vertical bar), 25th and 75th percentiles (lower and upper bounds of the box, respectively) and 1.5 x IQR (or minimum/maximum values; end of whiskers). **l**, Association of endothelial cell neighborhoods with CTAP-M and CTAP-F. For these CNA results, cells in UMAPs are colored in red (positive) or blue (negative) if their neighborhood is significantly associated with the CTAP (FDR < 0.05), and gray otherwise. Distributions of neighborhood correlations are shown for clusters with >50% of neighborhoods correlated with the CTAP at FDR < 0.05; global p-values were obtained based on the permutation testing from the CNA package.

Fibroblasts segregated broadly into lining (*PRG4*^hi^) and sublining (*THY1*^+^*PRG4*^low^) subsets and *NOTCH3*^*+*^*MCAM*^*+*^(CD146) mural cells (Fig. 2e and Extended Data Figs. 2 and 7a–f). As previously described, lining fibroblasts (F-0 and F-1) were depleted in rheumatoid arthritis relative to osteoarthritis and subdivided into *PRG4*^*+*^*CLIC5*^*+*^ (F-0), *PRG4*^*+*^ (F-1) and *RSPO3*^*+*^ (F-8) populations, the last exhibiting an intermediate lining–sublining phenotype. Sublining fibroblasts separated into *HLA-DRA*^*+*^, CD34^*+*^ and *DKK3*^*+*^ groups^8,32,33^. The CD34^*+*^ sublining fibroblast cluster (F-2) highly expressed *PI16* and *DPP4* (CD26), suggesting an undifferentiated, progenitor-like state^34^. *CXCL12*^*+*^ fibroblasts included an inflammatory *CD74*^hi^*HLA*^hi^ cluster (F-5) and a *CXCL12*^*+*^*SFRP1*^*+*^ cluster (F-6) with the highest levels of *IL6*, which encodes a proven drug target in rheumatoid arthritis.

Synovial endothelial cells separated into lymphatic endothelial cells and blood endothelial cells. Lymphatic endothelial cells (E-4), identified on the basis of high expression of the lymphatic markers *LYVE1* and *PROX1*, exhibited high expression of *CCL21* and *FLT4*^35,36^ (Fig. 2f and Extended Data Figs. 2 and 7g,k). Among blood endothelial cells, we observed several clusters along an arterial-to-venous axis, including *NOTCH4*^*+*^ arteriolar (E-3), *SPARC*^*+*^ capillary (E-0) and *CLU*^*+*^ venular (E-1 and E-2) cells. Arteriolar cells expressed high levels of *CXCL12, LTBP4, NOTCH4* and the NOTCH ligand *DLL4*. *SPARC*^*+*^ capillary cells expressed collagen and extracellular matrix genes. Venular cells further subdivided into *LIFR*^*+*^ (E-1) and *ICAM1*^*+*^ (E-2) and had high expression of inflammatory genes such as *IL6* and HLA genes, along with genes that facilitate leukocyte transmigration, such as *ICAM1* and *SELE* (E-selectin) (Extended Data Fig. 7i).

## CTAPs are defined by specific cell states

We used co-varying neighbourhood analysis (CNA) to identify single-cell-resolution ‘neighbourhoods’ associated with individual CTAPs. We use ‘expanded’ and ‘depleted’ to refer to differences in relative abundance within a cell type, accounting for age, sex and cell count per sample. Of note, this may not reflect a difference relative to total synovial cells. We tested each cell type for associations with all CTAPs, recognizing that even less enriched cell types may contain critical subsets.

**Fig. 3:**
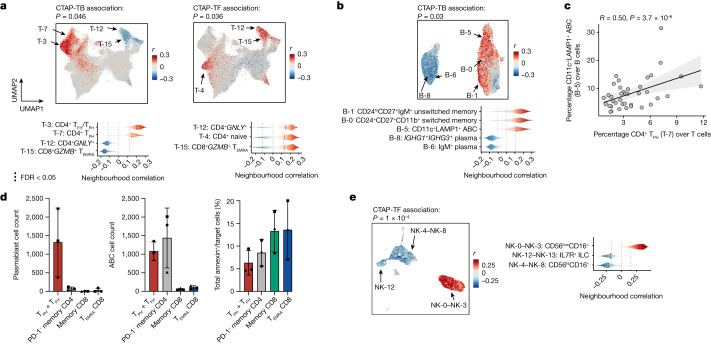
Different T cell, B cell and NK cell populations are associated with rheumatoid arthritis CTAPs. **a**, Associations of T cell neighbourhoods with CTAP-TB and CTAP-TF. *P* values are from the CNA test for each CTAP within T cells. **b**, Associations of B/plasma cell neighbourhoods with CTAP-TB. **c**, Percentage of T_PH_ (T-7) as a proportion of T cells and CD11c^+^ LAMP1^+^ ABCs (B-5) as a proportion of B/plasma cells for each donor sample. *R* and *P* values are calculated from Pearson correlation and two-sided *t*-tests, respectively. The shaded region represents 95% confidence interval. **d**, Plasmablast count (left), ABC count (centre) or percentage of annexin^+^ cells (right) stratified by co-cultured T cell subset. Points represent samples and shapes correspond to samples from the same donor, which were tested in independent experiments (*n* = 3). Data are mean ± s.d. **e**, Associations of NK cell neighbourhoods with CTAP-TF. **a**,**b**,**d**, For all CNA results, cells in UMAPs are coloured red (positive) or blue (negative) if their neighbourhood is significantly associated with the CTAP (false discovery rate (FDR) < 0.05), and grey otherwise. Distributions of neighbourhood correlations are shown for clusters with more than 50% of neighbourhoods correlated with the CTAP at FDR < 0.05. Global *P* values were obtained based on permutation testing from the CNA package.

We observed skewed T and B cell neighbourhoods in CTAP-TB (permutation *P* = 0.046 and 0.03, respectively) (Fig. 3a, Extended Data Fig. 3e, Supplementary Tables 7 and 8). T cell neighbourhoods among CD4^+^ T_FH_/T_PH_ (T-3) and CD4^+^ T_PH_ (T-7) cells were expanded, whereas neighbourhoods among cytotoxic CD4^+^*GNLY*^*+*^ (T-12) and CD8^+^*GZMB*^*+*^ cells (T-15) were depleted. Among B cells, we observed expanded neighbourhoods in memory B (B-0 and B-1) and ABC (B-5) clusters, whereas IgG1^+^IgG3^+^ and IgM^+^ plasma cells (B-8 and B-6) were relatively depleted (Fig. 3b and Extended Data Fig. 4e). We note that although plasma cells are depleted among B/plasma cells in CTAP-TB, plasma cells are enriched among total cells in CTAP-TB (4.1% compared with 0.6–3.1% in other CTAPs) (Extended Data Fig. 4e,f). Although T_PH_ (T-7), T_FH_/T_PH_ (T-3) and ABC (B-5) cells are enriched in CTAP-TB, they are present in all six CTAPs (Extended Data Figs. 3e and 4e). By contrast, germinal centre cells (B-4) were almost exclusively found in CTAP-TB (Extended Data Fig. 4e). Consistent with a role for T_FH_/T_PH_ and IL-21 in ABC generation^37^, the frequency of ABCs (B-5) amongst B/plasma cells correlated with the proportion of T_PH_ (T-7) and T_FH_/T_PH_ (T-3) among T cells (Pearson *r* = 0.50, *P* = 3.7 × 10^−6^ and Pearson *r* = 0.24, *P* = 0.034, respectively) (Fig. 3c and Extended Data Fig. 4g).

We hypothesized that the preferential enrichment of T_PH_ and T_FH_ cells in CTAP-TB reflected the ability of these subsets to sustain and activate B cells. To test this hypothesis, we sorted T_PH_ and T_FH_ cells and other memory CD4^+^ T cells, as well as CD45RA^+^ effector memory CD8^+^ T (T_EMRA_) cells and CD45RO^+^ memory CD8^+^ T cells, which are enriched for GZMB^+^ and GZMK^+^ CD8^+^ T cells, respectively^16^ from blood and co-cultured them with B cells and staphylococcal enterotoxin B superantigen (Fig. 3d, Extended Data Fig. 4h and Supplementary Fig. 8). T_PH_ and T_FH_ cells efficiently induced B cell differentiation into plasmablast and ABC phenotypes. Notably, non-T_FH_/T_PH_ memory CD4^+^ T cells were also able to induce ABC differentiation, but not plasmablast differentiation. CD8^+^ T cells did not induce B cell differentiation despite being functionally potent in cytotoxicity assays.

T cell neighbourhoods enriched in CTAP-TF (permutation *P* = 0.036) consisted mainly of cytotoxic CD4^+^*GNLY*^+^ (T-12) and CD8^+^*GZMB*^+^ cells (T-15) as well as naive CD4^+^ and CD8^+^ T cells (T-4 and T-16) (Fig. 3a, Extended Data Fig. 3e and Supplementary Tables 7 and 8). *GZMB*-expressing CD56^low^CD16^+^ NK cells (NK-0–3) were also enriched in CTAP-TF, and the proportion of *GZMB*^*+*^ NK cells (NK-0–3) correlated with the proportion of *GZMB*^*+*^ T cells (T-15) (Pearson *r* = 0.63, *P* = 4.87 × 10^−10^; Fig. 3e and Extended Data Fig. 5g). Conversely, *GZMK*^+^ CD8^+^ T cells (T-13 and T-14) correlated with *GZMK*^*+*^ NK cells (NK-4–8, Pearson *r* = 0.51, *P* = 1.41 × 10^−6^), suggesting that GZMB- and *GZMK*-expressing CD8^+^ T and NK cells share a transcriptional programme influenced by their tissue environments.

**Fig. 4:**
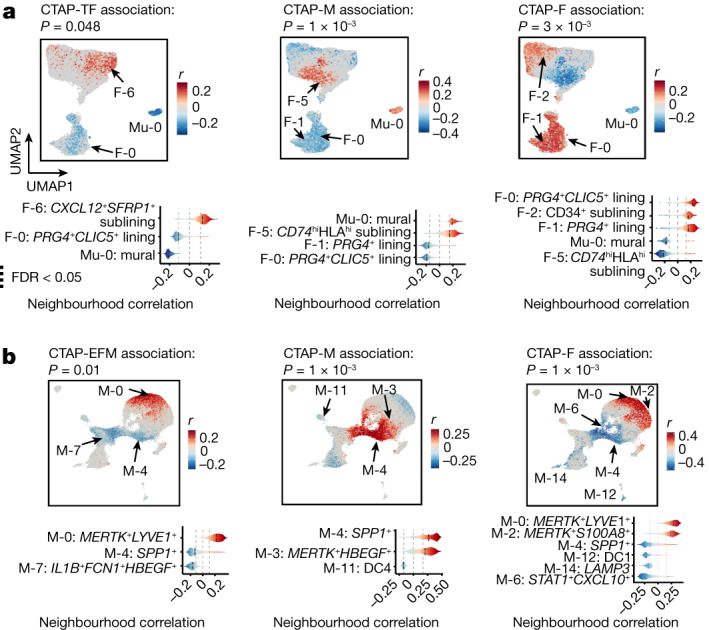
Different stromal, myeloid and endothelial cell populations are associated with rheumatoid arthritis CTAPs. **a**, Association of stromal cell neighbourhoods with CTAP-TF, CTAP-M and CTAP-F. **b**, Association of myeloid cell neighbourhoods with CTAP-EFM, CTAP-M and CTAP-F for all CNA results. Cells in UMAPs are coloured red (positive) or blue (negative) if their neighbourhood is significantly associated with the CTAP (FDR < 0.05), and grey otherwise. Distributions of neighbourhood correlations are shown for clusters with more than 50% of neighbourhoods correlated with the CTAP at FDR < 0.05. Global *P* values were obtained based on the permutation testing from the CNA package.

CTAP-TF also exhibited specific expansion among *CXCL12*^*+*^*SFRP1*^*+*^ sublining fibroblasts (F-6), which expressed *IL6* but not HLA-DR genes (Fig. 4a and Extended Data Fig. 7c). By contrast, CTAP-M demonstrated enrichment of *CD74*^hi^*HLA*^hi^ sublining fibroblast neighbourhoods (F-5) among stromal cells (permutation *P* = 10^−3^). We also observed that *SPARC*^+^ capillary cells (E-0) were expanded among endothelial cells in CTAP-M (permutation *P* = 7 × 10^−3^; Extended Data Fig. 7l).

Among myeloid populations, cell neighbourhoods within *SPP1*^*+*^ (M-4) and *MERTK*^*+*^*HBEGF*^*+*^ (M-3) macrophages were enriched in CTAP-M, suggesting recruitment of inflammatory monocytes and transition to macrophage function (Fig. 4b). Pro-inflammatory *IL1B*^*+*^ macrophages (M-7), known to be expanded in patients with rheumatoid arthritis in general^8^, were less frequent in CTAP-EFM relative to other CTAPs.

Of note, CTAP-M and CTAP-F exhibited contrasting cell enrichments and depletions across three cell types. (Fig. 4a,b and Extended Data Fig. 7l). Specifically, lining (F-0 and F-1) and CD34^+^ sublining (F-2) fibroblasts (permutation *P* = 3 × 10^−3^), *MERTK*^*+*^*LYVE1*^*+*^ (M-0) and *MERTK*^*+*^*S100A8*^*+*^ (M-2) macrophages (permutation *P* = 10^−3^), and *LIFR*^*+*^ venular (E-1) and *ICAM1*^*+*^ venular (E-2) endothelial cells were expanded in CTAP-F (permutation *P* = 3 × 10^−3^) and depleted in CTAP-M.

Given their high plasticity, we hypothesized that monocytes entering synovial tissue are shaped by the network of cell types and soluble factors associated with each CTAP. We tested this concept for CTAP-M and CTAP-TM by exposing human blood CD14^+^ monocytes to factors enriched in these tissues and then examining which CTAP-associated myeloid state these cells resembled (Extended Data Fig. 6g). We found that activated CD8^+^ T cell factors that mark CTAP-TM induced a set of genes that mark the *STAT1*^+^*CXCL10*^+^ macrophage state that is enriched in CTAP-TM (Extended Data Fig. 6h,i). Conversely, factors enriched in CTAP-M, including M-CSF, TGFβ and fibroblasts, drove monocytes towards the *MERTK*^+^*HBEGF*^+^ phenotype that is enriched in CTAP-M.

## Cell states are associated with histology

**Fig. 5:**
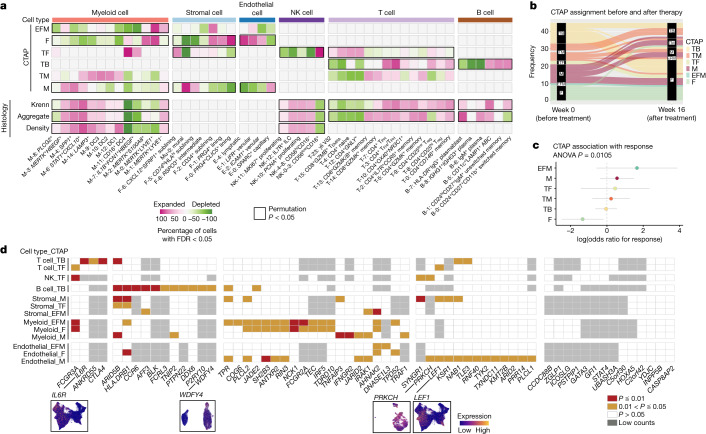
Single-cell CNA reveals significant association of cell states with disease indicators, genetic factors and treatment response. **a**, Heat map of CNA associations of specific cell states with each rheumatoid arthritis CTAP. Colours represent the percentage of cell neighbourhoods from each cell state with local (neighbourhood-level) phenotype correlations passing FDR < 0.05 significance from white to pink (expanded) or green (depleted). Cell types significantly associated globally (at cell-type level) with a phenotype at permutation *P* < 0.05 are boxed in black. **b**, Alluvial plot showing CTAP classification of samples prior to and at week 16 after starting treatment with either tocilizumab or rituximab (*n* = 45). **c**, Associations between clinical response and CTAPs after correcting for sex, age, treatment and CCP status in the baseline (week 0) samples from the R4RA study (*n* = 133). The percentage of variance explained by CTAPs alone and *P* value are calculated with ANOVA tests. Dots represent odds ratios and bars represent 95% confidence intervals. **d**, Significance of correlations between rheumatoid arthritis risk gene expression and CTAP-associated cells. Significance levels are shown in red (*P* < 0.01), yellow (0.01 < *P* < 0.05), and white (*P* > 0.05). Genes with low counts (more than one unique molecular identifier among less than 5% of cells with a given cell type) were not analysed in that cell type (grey boxes). Bottom, UMAPs displaying normalized expression levels of selected genes in T cells (*IL6R* and *LEF1*), B cells (*WDFY4*) and endothelial cells (*PRKCH*).

We used CNA to test for cell neighbourhoods associated with histologic features of rheumatoid arthritis synovium, including Krenn scores and discrete histologic cell density and aggregate scores reflecting inflammatory cell infiltration and organization (Fig. 5a, Supplementary Fig. 9a and Methods). Several T cell states were associated with aggregate scores (permutation *P* = 0.0088), including neighbourhoods among CD4^+^ T_FH_/T_PH_ (T-3), *GZMK*^+^CD8^+^ T cells, and some memory CD4^+^ T cells (Fig. 5a, Supplementary Fig. 9b and Supplementary Table 7). A *GZMK*^+^ NK cell cluster, NK-4, was associated with both density and aggregate scores (permutation *P* = 3 × 10^−4^ and 10^−4^, respectively) (Supplementary Fig. 9b). Neighbourhoods within *STAT1*^+^*CXCL10*^+^ (M-6), *SPP1*^+^ (M-4) and inflammatory DC3 (M-9) (Fig. 5a and Supplementary Fig. 9b) were associated with both aggregate and density scores (permutation *P* = 0.006 and *P* = 0.005, respectively). Among B cells, IgM^+^ plasma cells (B-6), plasmablasts (B-7) and ABCs (B-5) were associated with aggregate scores (permutation *P* = 0.007) (Fig. 5a and Supplementary Fig. 9b). These disparate cell-state associations with aggregate scores probably reflect the diverse composition of aggregates, which can be T cell-dominant, plasma cell-dominant or T and B cell follicles^38,39^.

**Extended Data Fig. 8:**
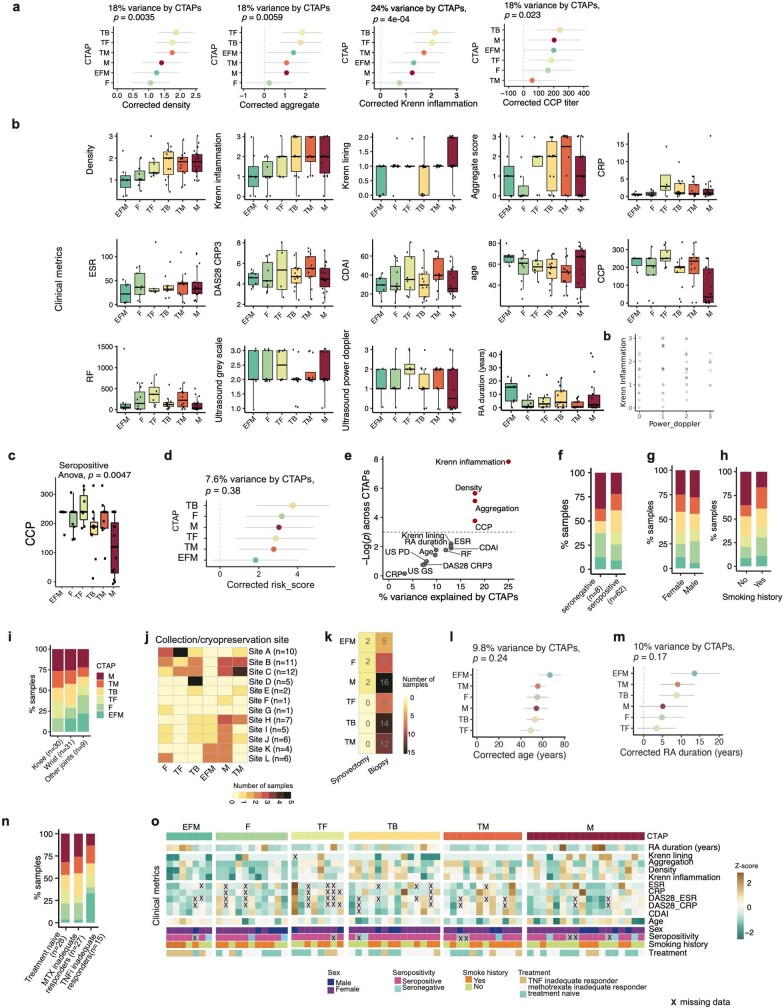
Association of single-cell RA CTAPs with different clinical characteristics. **a**, Associations between clinical features and CTAPs (N = 70), adjusting covariates for age, sex, cell number, and clinical collection site. Percentage of variance explained by CTAPs alone and p-value are calculated with ANOVA tests. Points represent odds ratios and bars represent 95% confidence intervals. **b**, Clinical, histologic, and ultrasound parameters of patients in each CTAP. For all box plots, each dot represents an individual (N = 70); boxes show median (vertical bar), 25th and 75th percentiles (lower and upper bounds of the box, respectively) and 1.5 x IQR (or minimum/maximum values; end of whiskers), **c**, Dotplot of Krenn inflammation versus power doppler scores. Each point is a patient. **d**, CCP levels among seropositive patients alone (N = 59). Points represent individuals and box plots show median (vertical bar), 25th and 75th percentiles (lower and upper bounds of the box, respectively) and 1.5 x IQR (or minimum/maximum values; end of whiskers)., **e**, Corrected RA *HLA-DRB1* risk scores and their associations with CTAPs, percent of variance explained by CTAPs only and p-value are calculated with ANOVA test, **f**, Clinical, demographic, and histologic metrics plotted by percentage of variance explained by CTAPs and the ANOVA p-value for its association with CTAPs. Features in red are significant at *p* < 0.05. **g**, CTAP frequency among seropositive (CCP-positive, RF-positive, or both) versus seronegative patients. **h**, CTAP frequency by sex. **i**, CTAP frequency by smoking history, **j**, CTAP frequency by anatomic site of synovial biopsy. **k**, Number of samples per CTAP in each collection/cryopreservation site. **l**, Number of patient samples for each CTAP between biopsy and synovectomy, **m**-**n**, Association of age and RA duration with CTAPs (N = 70), adjusting covariates for age, sex, cell number, and clinical collection site. Points represent odds ratios and bars represent 95% confidence intervals. Percentage of variance explained by CTAPs alone and p-values are calculated with one-way ANOVA tests. **o**, Sample distributions across CTAPs by recruitment cohort, **p**, Heatmap of clinical variables for patient samples grouped by CTAP. Boxes are colored based on z-score of the metric across samples. “X” represents missing data.

After accounting for age, sex, cell count and clinical collection site (Methods), we found that CTAPs account for 18% of variance of histologic density (*P* = 0.0035) and 18% of variance for aggregates (*P* = 0.0059), with CTAP-TB and CTAP-TF having the highest scores for both (Extended Data Fig. 8a,b). Consistent with these observations, CTAPs are associated with Krenn inflammation scores (*P* = 4 × 10^−4^), but not with Krenn lining scores (*P* = 0.11) (Extended Data Fig. 8a,b). Ultrasound measurements in the biopsied joint did not vary by CTAP (Extended Data Fig. 8b). In our dataset, we observed no association between Krenn inflammation and power doppler scores, consistent with some previous studies^40–42^ (Extended Data Fig. 8c).

## CTAPs are largely independent of clinical metrics

Cyclic citrullinated peptide (CCP) autoantibodies are known to confer a higher risk of severe disease and radiographic progression^43^. CCP titre values differed across CTAPs (*P* = 0.023, 18% variance), with CTAP-M having the lowest CCP titres, even after restricting the analysis to seropositive patients (*P* = 0.0047) (Extended Data Fig. 8a,d). *HLA-DRB1* is the strongest genetic rheumatoid arthritis risk factor for seropositive disease, yet we did not find that *HLA-DRB1* risk alleles were associated with a particular CTAP, although there was a trend toward association with CTAP-TB (Extended Data Fig. 8e and Methods).

We did not find a significant association between CTAPs and disease activity score-28 for rheumatoid arthritis with C-reactive protein (DAS28-CRP) or CDAI (Extended Data Fig. 8b), although our patient cohort is not ideal for testing such associations because it only includes patients with high disease activity. CTAPs were also independent of other clinical factors, smoking history and sex, and mostly independent of anatomic category and clinical site (Extended Data Fig. 8b,f–l and Supplementary Table 9). Patients with CTAP-EFM had statistically nonsignificant trends to be older, have longer-standing rheumatoid arthritis and be inadequate responders to TNF inhibitors (Extended Data Fig. 8m–p).

## CTAPs have disease-relevant cytokine profiles

**Extended Data Fig. 9:**
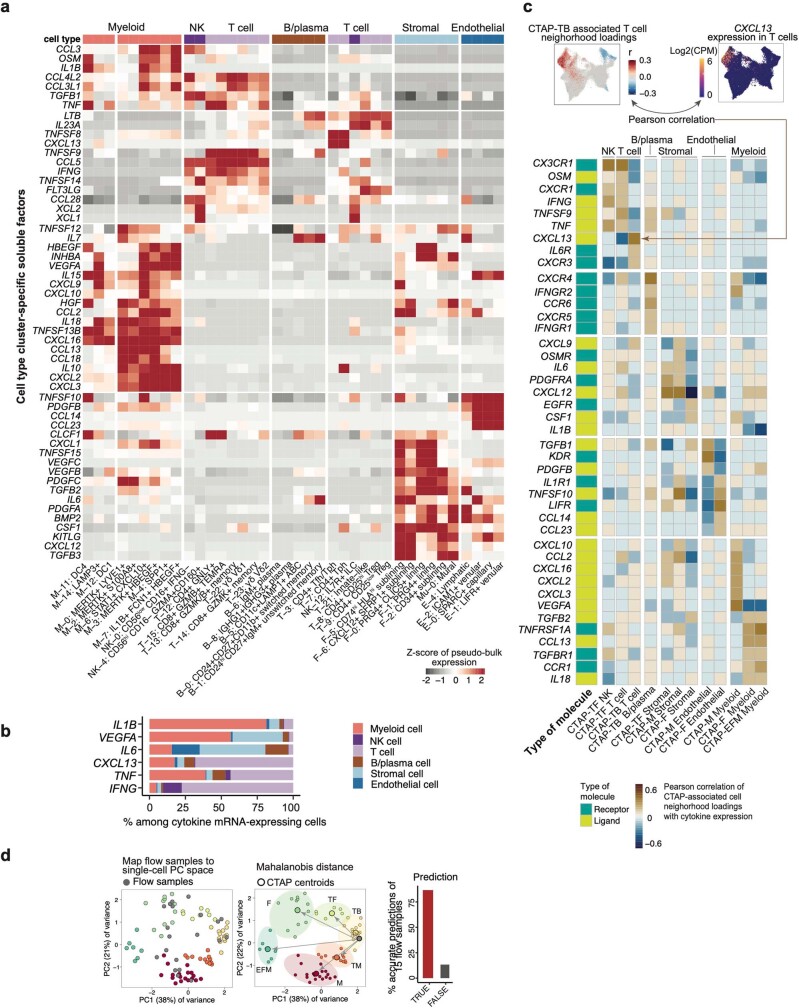
Correlations of cytokines/receptors with CTAP-associated cells. **a**, Heatmap depicting expression profiles of cell type cluster-specific soluble factors, **b**, Percent contribution among cytokine mRNA-expressing cells from each major cell type, **c**, At top, expression of *CXCL13*, a representative cytokine that is significantly correlated with CTAP-associated cell neighborhoods. Cells in UMAPs of CTAP associations are colored in red (positive) or blue (negative) if their neighborhood is significantly associated with the CTAP (FDR < 0.05), and gray otherwise. Cells in expression UMAPs are colored from blue (low) to yellow (high). Below, an aggregate heatmap visualizing the cytokines and receptors whose expressions are significantly correlated (r > 0.5) with CTAP-associated cells; we then hierarchically clustered them based on cell type-specific CTAPs. Each gene is labeled with receptor/ligand designation. **d**, Pipeline and results to map and classify flow cytometry samples by single-cell RA CTAPs. Bar plot shows accuracy of flow sample classification (i.e., assigned to the same CTAP as a single-cell sample from the same patient).

We next analysed transcript levels of cytokines, chemokines, and their receptors, recognizing that these transcripts are often sparse in single-cell RNA-seq data (Supplementary Fig. 10). Most cytokines and chemokines are detected predominantly in one cell type, although some key cytokines were produced by multiple cell types (Extended Data Fig. 9a,b). For example, we detected *TNF* in roughly equal numbers of T cells and myeloid cells, whereas fibroblasts, endothelial cells and B cells dominated among cells with detectable *IL6*.

Next, we correlated CTAP neighbourhood association scores with the expression of key cytokines and receptors to identify soluble factors produced by CTAP-associated cell states. For example, as predicted, CTAP-TB, enriched for T_FH_/T_PH_ cell states, had T cell neighbourhood association scores that correlated with expression of the T_FH_/T_PH_ marker *CXCL13* (Fig. 3a and Extended Data Fig. 9c). By contrast, CTAP-TF-associated *GZMB*^+^ T and NK cell neighbourhoods had association scores correlating with the expression of *IFNG* and *TNF* (Fig. 3a,e and Extended Data Fig. 9c), suggesting that these cytokines may be key molecular drivers of CTAP-TF.

In some CTAPs, this analysis revealed potential cytokine networks. For example, in CTAP-M, myeloid neighbourhood association scores correlated with expression of angiogenic factor *VEGFA*, whereas endothelial cell neighbourhood association scores correlated with expression of *KDR* (also known as *VEGFR2*), potentially explaining the observed enrichment of capillaries in this CTAP (Extended Data Figs. 7l and 9c). By contrast, in CTAP-F, enriched *LIFR*^+^ and *ICAM1*^+^ venular endothelial cell neighbourhoods expressed high levels of *CCL14*, whose cognate receptor *CCR1* was highly expressed by *MERTK*^+^ macrophage neighbourhoods, which are also enriched in CTAP-F (Fig. 4b and Extended Data Fig. 7l and Fig. 9c). Cell–cell communication analysis confirmed these putative interactions (Supplementary Fig. 11).

Our study included three patients with replicate biopsies obtained from the same joint 98 to 190 days after the initial biopsy. Cell-type composition of repeat biopsies was similar to the initial biopsy (permutation *P* = 0.073) (Supplementary Fig. 12a,b), but more samples are needed to understand how dynamic CTAPs are.

## Mapping CTAPs to other patient cohort data

To enable investigation of these and other CTAP-related questions in larger studies, we examined whether samples can be classified into CTAPs using lower-resolution technologies such as flow cytometry and bulk tissue RNA-seq. We first built a nearest-neighbour classifier for flow cytometry data and were able to accurately replicate CITE-seq-based CTAP assignments (accuracy = 87%; Extended Data Fig. 9d, Supplementary Fig. 12c,d and Supplementary Table 10).

**Extended Data Fig. 10:**
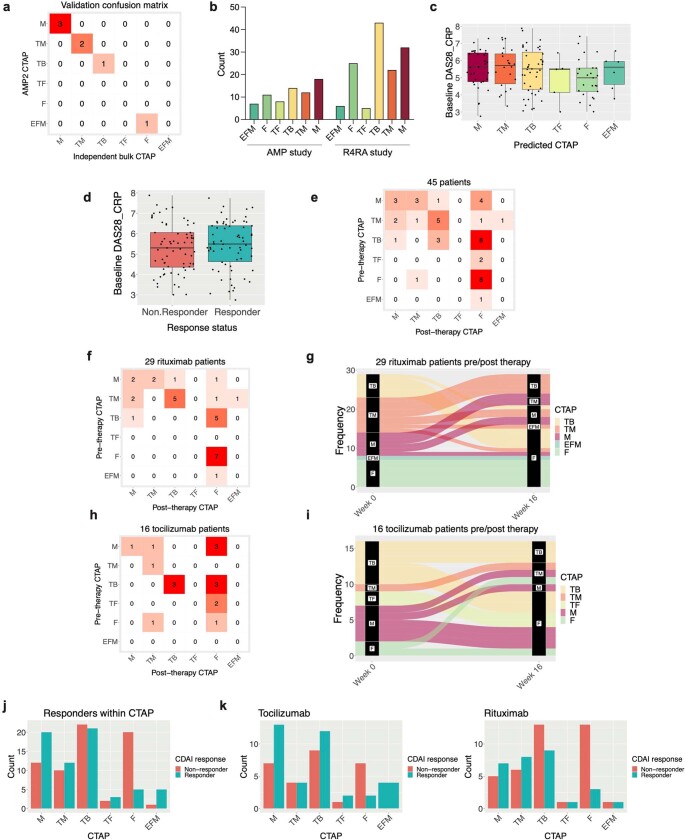
Assigning CTAP labels to bulk RNA-seq samples and clinical association analysis. **a**, Confusion matrix showing CTAP assignment by the single-cell CITE-seq panel (gold standard) versus classification of synovial tissue bulk RNA-seq obtained from the same individuals (N = 7). **b**, Patients per CTAP category in the current AMP study, which enrolled a clinically diverse patient cohort, versus the published R4RA study, which restricted enrollment to patients with inadequate response to TNF inhibitor therapies. **c**, Baseline DAS28-CRP scores stratified by predicted CTAP (N = 133 patients). **d**, Baseline DAS28-CRP score stratified by clinical response status ( ≥ 50% improved CDAI after treatment) (N = 133 patients). In **c** and **d**, Points represent individuals and box plots show median (vertical bar), 25th and 75th percentiles (lower and upper bounds of the box, respectively) and 1.5 x IQR (or minimum/maximum values; end of whiskers). **e**, Confusion matrix showing predicted CTAP assignment of pre-treatment (week 0) and post-treatment (week 16) synovial tissue samples obtained from 45 patients. **f**-**g**, Confusion matrix and alluvial plot showing predicted CTAP assignment before and after treatment with rituximab (N = 29). **h**-**i**, Confusion matrix and alluvial plot showing predicted CTAP assignment before and after treatment with tocilizumab (N = 16). **j**, Graph of responder and non-responders stratified by CTAP (N = 133) among all patients in the R4RA study. **k**, Graph of responders and non-responders among patients receiving tocilizumab (left, N = 65) or rituximab (right, N = 68), stratified by CTAP.

We next developed a method to classify CTAPs using bulk RNA-seq data of intact synovial tissue from a recent clinical trial^6^. CTAP classification based on bulk RNA-seq agreed with the CITE-seq-based CTAP assignment for 6 out of 7 samples in the present study that were also analysed with bulk RNA-seq (Extended Data Fig. 10a).

We applied our CTAP classification algorithm to bulk RNA-seq profiles from the R4RA clinical trial comparing rituximab and tocilizumab for the treatment of patients with rheumatoid arthritis with inadequate response to TNF inhibitor therapy^44^ (*n* = 133). The distribution of CTAPs differs between these datasets, probably reflecting differences in cohort recruitment criteria (Extended Data Fig. 10b). As in our cohort, we found no association between CTAP assignment and disease activity or between treatment response and disease activity (Extended Data Fig. 10c,d), supporting our hypothesis that CTAPs reflect distinct inflammatory phenotypes driving arthritis rather than differences in clinical disease activity.

To investigate whether CTAPs change over time, we applied our CTAP classification algorithm to 45 patients from the R4RA trial who had synovial tissue biopsies before and 16 weeks after starting treatment. CTAPs were dynamic during this period, with 30 out of 45 (67%) patients changing to a different CTAP (Fig. 5b and Extended Data Fig. 10e). Patients in the tocilizumab and rituximab treatment arms exhibited similar frequencies of CTAP change (20 out of 29 (69%) and 10 out of 16 (63%) patients, respectively) (Extended Data Fig. 10f–i). Among patients who changed CTAPs, CTAP-F was the most common CTAP at week 16 (16 out of 30 (53%)), consistent with rituximab and tocilizumab targeting inflammatory cells and pathways.

## Response to biologic therapy varies by CTAP

To determine whether CTAPs can predict the response to these treatments, we used our algorithm to determine the CTAPs of pre-treatment bulk RNA-seq for R4RA samples (*n* = 133). We then compared the frequencies of responders (defined as at least 50% improvement in CDAI) versus non-responders among the CTAPs (Extended Data Fig. 10j,k). We found that responses varied by CTAP (*P* = 0.0105), with CTAP-F having the poorest response to both treatments, even after controlling for covariates (odds ratio = 0.2619, *P* = 0.0403; Fig. 5c).

## CTAP-enriched cell states express risk genes

We next tested whether genes implicated by recent multi-ancestry rheumatoid arthritis genetic studies are preferentially expressed by cell states associated with specific CTAPs^45,46^. We identified 71 genes that were likely to be causal, all of which were detected in one or more cell types in our dataset (Methods, Supplementary Fig. 13a and Supplementary Table 11).

We identified 48 genes with expression that was significantly positively correlated with CNA loadings for one or more CTAPs for a cell type (*P* < 0.05, controlling for expression level), indicating that cell states expanded in that CTAP specifically express the rheumatoid arthritis risk gene (Fig. 5d). This is significantly higher than predicted by chance (median = 34, permutation *P* < 0.01; Supplementary Fig. 13b,c). Some cell types expressed different rheumatoid arthritis genes in different subsets of cells (for example, *LEF1* in CTAP-TF-associated naive states and *IL6R* in CTAP-TB-associated T_FH_/T_PH_ states). *HLA-DRB1* expression was correlated with CTAP-associated cell states in several cell types (Fig. 5d). CTAP-associated rheumatoid arthritis risk genes may also be expressed agnostic of CTAP in a given cell type, such as *IL6R* in myeloid cells (Supplementary Fig. 13d).

Some genes point to signalling pathways that may be important in a specific CTAP, such as VEGF in CTAP-M (Extended Data Fig. 9c). *PRKCH*—which encodes protein kinase C (PKC)-η, a mediator of VEGF-induced endothelial cell differentiation^47^—is highly expressed in endothelial cell states expanded in CTAP-M, which has high expression of VEGF receptor genes *KDR* and *FLT1* among expanded endothelial cell states and *VEGFA* among expanded myeloid cell states (Fig. 5d and Supplementary Fig. 13e–g).

## Discussion

We constructed a comprehensive rheumatoid arthritis synovial tissue reference of more than 314,000 single cells which revealed diverse cellular composition that we characterized into six CTAPs. Previously identified pathogenic cell states in rheumatoid arthritis are expanded in specific CTAPs. For example, CD4^+^ T_FH_ and T_PH_ cells, which are enriched among T cells in rheumatoid arthritis compared with osteoarthritis^11^, are present in synovium of all CTAPs but are most expanded in CTAP-TB. Our work also suggests the presence of extra-follicular activation pathways, especially in CTAP-TB, given the rarity of germinal centre dark-zone B cells and abundance of ABCs. Our study also provided more granular insights into previously identified pathogenic cells. For example, inflammatory sublining fibroblast subsets *CXCL12*^*+*^ and *CD74*^hi^*HLA*^hi^ cells were enriched in CTAP-TF and CTAP-M, respectively. *MERTK*^*+*^*HBEGF*^*+*^ and *SPP1*^*+*^ macrophages were also enriched in CTAP-M, probably reflecting different inflammatory axes. These and other instances of co-enriched populations (for example, *GZMK*^+^ versus *GZMB*^+^CD8^+^ T and NK cells) inspire new questions about cell–cell interactions underlying inflammatory phenotypes in rheumatoid arthritis and other tissues and diseases.

We found that CTAPs are associated with histologic and serologic (CCP) parameters, in line with studies^48^ that report increased lymphocyte infiltration (suggesting CTAP-TB, CTAP-TF or CTAP-TM) in CCP-positive synovium compared with CCP-negative synovium. Our finding that CTAP-M, and not CTAP-F or CTAP-EFM, was associated with CCP-negative status warrants further investigation in future studies.

CTAPs can be inferred from single-cell RNA-seq, bulk RNA-seq or flow cytometry data to provide cellular and molecular insights in clinical trials. Even within the more limited clinical diversity of the R4RA cohort^44^, we found that CTAPs can change over time with treatment, and that CTAP-F was associated with poor clinical response. The dynamic heterogeneity of rheumatoid arthritis synovitis may explain the observation that clinical measures of patients treated with TNF inhibitors do not fall into a bimodal distribution of responders and non-responders^49^. It is possible that specific CTAPs are more likely to respond to specific therapies that preferentially target infiltrating cell types and relevant pathways. We anticipate that future longitudinal studies will investigate the association of CTAP changes with treatment effects across a larger array of treatments.

The CTAP paradigm provides a tissue classification system that captures coarse cell-type and fine cell-state heterogeneity. This model has the potential to serve as a powerful prototype to classify other types of tissue inflammation, including other immune-mediated diseases. A deeper understanding of the heterogeneity of tissue inflammation in rheumatoid arthritis and other autoimmune diseases may provide new insights into disease pathogenesis and reveal new treatment targets, and key elements of precision medicine.

### Reporting summary

Further information on research design is available in the Nature Portfolio Reporting Summary linked to this article.

## Online content

Any methods, additional references, Nature Portfolio reporting summaries, source data, extended data, supplementary information, acknowledgements, peer review information; details of author contributions and competing interests; and statements of data and code availability are available at 10.1038/s41586-023-06708-y.

### Supplementary information

Supplementary Information This file contains Supplementary Methods, Supplementary Figs. 1–3 and 5–13, Supplementary Tables 1–4, 6, 9 and 10 and Supplementary References.

Reporting Summary

Supplementary Figure 4

Peer Review File

Supplementary Table 5

Supplementary Table 7

Supplementary Table 8

Supplementary Table 11 Genes associated with RA risk alleles.

## Data Availability

CITE-seq single-cell expression matrices and sequencing and bulk expression matrices are available on Synapse (10.7303/syn52297840). Associated genotype and clinical data are available through the Arthritis and Autoimmune and Related Diseases Knowledge Portal (ARK Portal, https://arkportal.synapse.org/Explore/Datasets/DetailsPage?id=syn52297840). A cell browser website https://immunogenomics.io/ampra2/ is available to visualize our data and results. AMP Phase 1 single-cell data from ref. ^8^ are available on Immport (SDY998). PEAC clinical trial RNA-seq data from ref. ^6^ are available on ArrayExpress (E-MTAB-6141). R4RA clinical trial RNA-seq data from ref. ^44^ are available on ArrayExpress (E-MTAB-11611). Single-cell and bulk RNA-seq data were aligned to GRCh38 (Ensembl 93), available as part of Cell Ranger v. 3.1.0.

## Code availability

The source code for the analyses is available at https://github.com/Immunogenomics/RA_Atlas_CITEseq/ and https://zenodo.org/record/8118599.
