# Supplementary material for: Deconstruction of rheumatoid arthritis synovium defines inflammatory subtypes
Source: Nature. 2023 Nov 8;623(7987):616–24. doi: 10.1038/s41586-023-06708-y (PMC10651487; doi:10.1038/s41586-023-06708-y)
Supplement: Supplementary file 1 — This file contains Supplementary Methods, Supplementary Figs. 1–3 and 5–13, Supplementary Tables 1–4, 6, 9 and 10 and Supplementary References. [file 41586_2023_6708_MOESM1_ESM.pdf]

nature portfolio

https://doi.org/10.1038/s41586-023-06708-y

Supplementary information

Deconstruction of rheumatoid arthritis synovium defines inflammatory subtypes

In the format provided by the authors and unedited

Supplementary Information

Table of Contents

Supplementary Methods…………………………...2
Supplementary Figures 1-3 and 5-13……………17
Supplementary Tables 1-4, 6, and 9-10…..……..42
Supplementary References……………………….53

Supplementary Figure 4 and Supplementary Tables 5, 7, and 8 are provided separately.

Supplementary Methods

RA patient recruitment and clinical data collection

The Accelerating Medicines Partnership (AMP) Network for RA and SLE constructed a cross-sectional cohort - samples were collected from 13 clinical sites across the United States and 2 sites in the United Kingdom. Patients were required to be aged ≥18 years, meet a diagnosis of RA by ACR/EULAR 2010 and/or 1987 criteria, and have at least two active inflamed joints (including the sentinel joint), a minimum CDAI >10, and an ultrasound gray scale ≥2 for QuickCore or ≥1 for portal and forceps biopsies. Patients were recruited to three groups based on their treatment status: (1) ≤ 4 weeks of MTX at (15mg once a week) and/or ≤ 4 weeks on any other nbDMARD (2) ≥ 3 months of MTX and ≥15mg once a week for 1 of the 3 months and stable dose ≥4 weeks; (3) ≥3 months on a TNFi with up to 2 TNFi failures allowed. The collection occurred over the course of a 45-month period from October 2016 to February of 2020. The study was performed with informed consent in accordance with protocols approved by the institutional review board at Stanford University. Demographics, RA clinical data, clinical assessments, and measurements of ESR and CRP were performed at the baseline visit [50]. Data collected include age, sex, RA duration, RF or anti-CCP status, RA treatments, tender and swollen joint counts. ESR and CRP were measured using commercial assays in each institution's clinical laboratory. Disease activity for each subject was calculated using a DAS28-CRP3 validated instrument[51,52]. Clinical data were managed using RedCap version 6.9.0 through 13.8.1.

Synovial tissue collection and processing

Synovial tissue samples were obtained from ultrasound-guided biopsies or surgical procedures. Of the 82 samples that completed the tissue processing pipeline, 54 samples were biopsies obtained with a Quick-Core needle, 15 samples were biopsies obtained with portal and forceps, 10 samples were collected during arthroplasty surgery, and 6 samples were collected during surgical synovectomy procedures. All specimens consisted of a median of 13 samples (range 4-36), of which 6-8 fragments were fixed in formalin for subsequent paraffin embedding and processing for histologic analysis. The remaining fragments were cryopreserved in one or more aliquots in Cryostor CS10 (Sigma-Aldrich) cryopreservation media. Samples were shipped to a central biorepository site until sample collection was complete. They were then transited to the central pipeline site, where samples were thawed and processed in batches.

Histology assessment, definition of density and aggregation for RA synovium

In order to exclude low-quality synovial tissue samples from our multi-omics tissue processing platform, we analyzed hematoxylin and eosin-stained slides of formalin-fixed, paraffin-embedded synovial tissue from each patient. At least six tissue fragments per patient were included in the analysis to mitigate sampling bias. Synovial tissue was identified as previously described[8], and samples that lacked any discernible synovial tissue were excluded from further analysis. To separate histologic domains of the density of the infiltrate and the extent of formation of discrete aggregates that are not distinguished by the Krenn inflammatory infiltrate domain, we developed consensus semiquantitative four point scales for density and aggregate radial size with a custom atlas using a test set of tissues from the Birmingham Early Arthritis

Cohort[53], scored by three pathologists. This approach was validated by scoring tissues from the first AMP RA cohort[8], achieving an intra-class correlation coefficient score of 0.896 for summary mean density score of fragments for each tissue and kappa 0.862 for the worst case aggregate score achieved in each tissue. Equivalent ICC figures for the summary mean scores of fragments for Krenn inflammatory domain and Lining layer thickness domains were 0.937 and 0.646 respectively. Three pathologists independently determined Krenn lining and inflammatory infiltrates scores (0-3 each)[54], cellular density scores (0-3), and aggregate (0-3) scores for each tissue sample, and the mode of the three scores was used for further analysis.

Tissue disaggregation, live cell sorting, and cell allocations

For pipeline analysis, cryopreserved synovial tissue samples were thawed and disaggregated into single-cell suspension as previously described[55]. Briefly, thawed synovial tissue fragments were mechanically and enzymatically separated in digestion buffer (Liberase TL (Roche) 100 µg/ml and DNase I (New England Biolabs) 100 µg/ml in RPMI) in 37°C water bath for 30 min. Single-cell suspensions from disaggregated synovial tissues were stained with anti-CD235a antibodies (clone 11E4B-7-6 (KC16), 1:100, Beckman Coulter) and Fixable Viability Dye eFlour 780 (eBioscience/ThermoFisher). Live non-erythrocyte cells (viability dye$^-$ CD235a$^-$) were isolated by fluorescence-activated cell sorting on a BD FACSAria Fusion using BD FACSDiva 8.0.1 software. Cells were allocated as follows, in order of priority: (1) 60,000 cells for CITE-seq analysis; (2) 50,000 cells for flow cytometry and bulk RNA-seq analysis; (3) remaining cells re-frozen in aliquots of 70,000 - 100,000 cells in CryoStor CS10 for other analyses (e.g. single-cell ATAC-seq and immune cell repertoire studies). Samples with fewer than 60,000 cells were applied to CITE-seq analysis alone.

Flow cytometry and bulk RNA-seq

Up to 50,000 sorted live synovial cells were stained with the following antibodies to define cell subsets: CD3 (UCHT1, 1:50), CD4 (OKT4, 1:50), CD8A (SK1, 1:100), CD11c (3.9, 1:50), CD14 (M5E2, 1:200), CD19 (HIB191:50), CD27 (M-T271, 1:100), CD31 (WM59, 1:200), CD45 (HI30, 1:200), CD90 (5E10, 1:200), CD146 (P1H12, 1:200), HLA-DR (L243, 1:50), PD-1 (EH12.2H7, 1:50) (**Supplementary Table 10**). All antibodies were purchased from Biolegend, and staining was performed in the presence of Fc block (eBioscience/ThermoFisher, True-Stain Monocyte Blocker (Biolegend), and Brilliant Stain Buffer (BD Bioscience). We collected flow cytometry data in conjunction with fluorescence-activated cell sorting of up to 1,000 B cells (CD45$^+$CD3$^-$CD14$^-$CD19$^+$), fibroblasts (CD45$^-$CD31$^-$CD146$^-$), myeloid cells (CD45$^+$CD3$^-$CD14$^+$), and T cells (CD45$^+$CD3$^+$CD14$^-$) on a BD FACSAria Fusion cell sorter BD FACSDiva 8.0.1 software.

Multicolor immunofluorescence staining and microscopy

Staining for lymphocyte immunofluorescence microscopy panel

Formalin-fixed paraffin-embedded synovial tissue fragments from 36 individuals (150 fragments total, mean 4.2 fragments per individual, range 2-9 fragments per individual) were incubated at 60°C for deparaffinization. Tissue sections were transferred to xylenes and gradually hydrated by sequentially transfer into alcohol, 95% alcohol, 70% alcohol and water. Sections were then immersed in antigen retrieval solution (S1699, DakoCytomation) and boiled for 30 minutes. Non-specific binding was blocked by incubating tissue sections with 5% normal donkey serum

(Jackson ImmunoResearch Laboratories) at room temperature (RT) for 30 minutes in a humid chamber. Primary antibodies were added to the tissues sections immediately after removing the blocking solution. Slides were incubated overnight at room temperature with the primary antibodies, which included goat anti-CD3 (1:100, Clone M-20, sc-1127, Santa Cruz Biotechnology), Rabbit anti-CD138 (1:50, PA5-32305, Thermo Fisher Scientific), and mouse anti-human CD20 (1:50, Clone L26, GTX29475, GeneTex). Tissue sections were washed with PBS and incubated for one hour at room temperature with secondary antibodies including Alexa Fluor 568-conjugated donkey anti-goat IgG (A-11057, Thermo Fisher Scientific), Alexa Fluor 488-conjugated donkey anti-rabbit IgG (711-546-152, Jackson ImmunoResearch), Alexa Fluor 647-conjugated donkey anti-mouse IgG (715-606-150, Jackson ImmunoResearch), all at 1:200 dilution. Synovial tissues were washed with PBS and incubated for 20 minutes with NucBlue Live Ready Probes Reagent (R37605, Thermo Fisher Scientific) to enhance nuclear stain. Synovial sections were finally mounted with Vectashield antifade mounting media with DAPI (H-1200, Vector Laboratories). Whole-slide immunofluorescent and bright field images were scanned at x20 magnification in a Whole Slide VS120 scanner (OLYMPUS, Center Valley, PA). A region of interest was chosen using the Visiomorph tissue detecting algorithm (Visiopharm 2022.01: Version 2022.01.3.12053), which automatically recognizes and outlines the tissues. We utilized multiple focus points and semi-automatic focusing for fluorescent whole slide images and multiple focus points and automatic focusing for bright field images.

Quantitation of immune cells in whole slide images of lymphocyte panel.
Images were pre-processed in Visiopharm (version 2022.01.3.12053) to adjust image threshold for optimal identification of nuclei. Polyblobs segmentation tool, tissue, and cell count apps were utilized for the accurate counting of nuclei and cells. Positively stained cells were rigorously identified by their morphological features: size (small = $CD20^+$ B and $CD3^+$ T cells vs. large = $CD138^+$ plasma cells), membrane stain ($CD20^+$ B and $CD3^+$ T cells) and cytoplasmic stain ($CD138^+$ plasma cells), and separately counted. We used bimodal segmentation for distinguishing pixels representing stained membrane from other pixels in the image. Images were then post-processed to merge membranes that were not completely connected and eliminate small membrane fragments. Proportions were calculated by dividing the number of positive cells by the total number of nuclei in tissues with high cell density.

Staining for stromal/myeloid immunofluorescence microscopy panel
Sections from the same tissue blocks from the same 36 individuals as the lymphocyte panel above were also staining for stromal and myeloid markers. Prior to staining, RA synovium tissue slides were deparaffinized on the Leica BOND RX automated immunostainer (Leica Microsystems, Milton Keynes, UK) by baking for 30 min at 60°C, soaking in BOND Dewax Solution at 72°C, and then rehydrating in ethanol. Staining was performed on the Leica BOND RX with HIER pretreatments applied at 95°C using BOND Epitope Retrieval (ER) Solutions: EDTA-based pH 9.0 ER2 (Leica Biosystems; Cat. No. AR9640). Multiplex tissue immunofluorescence (IF) staining for CLIC5 (1:50, clone 1E6, SAB1402589, Sigma), CD68 (1:50, clone 514H12, CD68-L-CE, Leica) CD3 (1:25, clone LN10, CD3-565-L-CE, Leica), HLA-DR (1:200, clone EPR3692, ab92511, Abcam), CD34 (1:100, clone QBEnd/10, END-L-CE, Leica) and CD90 (1:200, clone D3V8A, 13801, Cell Signaling Technology) was also then

performed on the Bond RX automated staining platform with the Opal 7-Color Automation IHC Kit (NEL871001KT, Akoya Biosciences) according to the manufacturer's instructions. Immunofluorescence signals for CLIC5, CD68, CD3, HLA-DR, CD34 and CD90 were visualized using TSA dyes 570, 520, 620, 480, 690 and 780 respectively, and counterstained with spectral DAPI. Slides were mounted with ProLong™ Diamond Antifade Mountant (Invitrogen, Cat. No. P36965). Multispectral images were acquired at ×20 magnification using the Vectra Polaris Automated Quantitative Pathology Imaging System (Akoya). MoTIF settings were used for multispectral image acquisition and to remove autofluorescence. Multispectral image processing of multiplex IHC stains was performed using Phenochart (version 1.0.11/Akoya) and inForm Image Analysis Software (version 2.3, Akoya).

Quantitation of cells in whole slide images of stromal/myeloid panel.
The Visiopharm platform (version 2022.10) was used to analyze images. This software relies on linking Analysis Protocol Packages (APPs) to analyze images in a stepwise fashion. APPs for general tissue detection, tissue compartmentalization to vessels and non-vessels areas using vessel morphology and CD34 staining (AI), cellular segmentation based on DAPI staining (AI), and automatic identification of phenotypes based on trained phenotyping classification were developed for the various steps of image analysis.

Quantitative histology analysis
Adjacent slices of 150 synovial tissue fragments from 36 synovial tissue samples were stained with a lymphocyte panel (CD20, CD138, CD3) and stromal cell panel (CD3, CD68, CLIC5, CD34, CD90, HLA-DR), as described above. The designation of $CD90^+$ cells excludes $CD34^+$ cells. We quantified CD3 staining from the stromal cell panel because of higher quality control parameters. For each CTAP, we filtered out low-quality or non-synovial fragments with low (<50%) cell density across all markers. We corrected for differences in fragment size and density by calculating the proportion of total cells positive for each marker within each fragment. We tested the association between proportion of cells positive for each marker and CTAP using ANOVA.

Single-cell CITE-seq antibody staining, RNA library preparation, and sequencing
CITE-seq[56] antibody staining using TotalSeq™-A antibodies was performed as per the recommended protocol (BioLegend). Briefly, we first curated a list of surface proteins based on markers of cell states identified in previous RA studies and TotalSeq™-A antibodies available at the time. To identify optimized concentrations of these antibodies for synovial tissue, we conducted a series of pilot studies where we titrated antibodies and measured their staining quality with mean expression (i.e., intensity) and Kullback-Leibler (K-L) divergence (i.e., specificity). We calculated K-L divergence by comparing the distribution across mRNA-defined cell clusters expressing the protein highly (>85th percentile) versus the null distribution of all cells. If an antibody had low mean staining and low K-L divergence, we removed it from the panel. If it had high mean staining and low K-L divergence, we used it at a lower concentration.

The optimized list of antibodies included CD107a/LAMP-1 (H4A3); CD314/NKG2D (1D11); CD19 (HIB19); CD8a (RPA-T8); CD21 (Bu32); IgG Fc (M1310G05); CD209/DC-SIGN (9E9A8);

EGFR (AY13); CD196/CCR6 (G034E3); CD1c (L161); CD309/VEGFR2 (7D4-6); CD127/IL-7Rα (A019D5); CD273/B7-DC/PD-L2 (24F.10C12); CD226/DNAM-1 (TX25); CD278/ICOS (C398.4A); CD119/IFN-γ R α chain (GIR-208); CD274/B7-H1/PD-L1 (29E.2A3); CD3 (UCHT1); CD55 (JS11); IgM (MHM-88); CD155/PVR (SKII.4); CD112/Nectin-2 (TX31); CD4 (SK3); CD11c (S-HCL-3); CD34 (581); CD90/Thy1 (5E10); CD45RA (HI100); CD16 (3G8); CD45RO (UCHL1); CD20 (2H7); Podoplanin (NC-08); CD140a/PDGFRα (16A1); CD146 (P1H12); CD195/CCR5 (J418F1); CD69 (FN50); CD161 (HP-3G10); HLA-DR (L243); CD64 (10.1); CD24 (ML5); CD192/CCR2 (K036C2); CD163 (GHI/61); CD44 (IM7); CD141/Thrombomodulin (M80); CD27 (LG.3A10); CD206/MMR (15-2); Folate Receptor β/FR-β (94b/FOLR2); CD45 (2D1); CD31 (WM59); CD11b (ICRF44); CD68 (Y1/82A); CD38 (HIT2); CD144/VE-Cadherin (BV9); CD304/Neuropilin-1 (12C2); CD86 (IT2.2); CD279/PD-1 (EH12.2H7); CX3CR1 (K0124E1); CD56/NCAM (QA17A16); CD14 (63D3). **Supplementary Table 2** lists these antibodies with barcode sequences, catalog numbers, and dilutions. Antibodies against CD107a (LAMP-1), CD314 (NKG2D), CD19, CD8a, CD21, IgG Fc, CD209 (DC-SIGN), EGFR, CD196 (CCR6), CD1c, CD309 (VEGFR2), CD127 (IL-7Rα), CD273 (B7-DC, PD-L2), CD226 (DNAM-1), CD278 (ICOS), CD119 (IFN-γ R α chain), CD274 (B7-H1, PD-L1), CD3, CD55, IgM were used at a dilution of 1:250 (0.2 ug per 100 uL staining reaction), whereas the remaining antibodies were used at a dilution of 1:50 (1 ug per 100 uL staining reaction). To perform CITEseq antibody staining, we prepared a cocktail of TotalSeq antibodies and centrifuged for 10 min at 14,000G to remove precipitates. Up to 60,000 sorted live synovial cells were pre-incubated with Human TruStain FcX (BioLegend) in Cell Staining Buffer (BioLegend) for 10 minutes prior to adding 100 uL of the antibody cocktail. Single-cell RNA-seq for all synovial samples was performed by the BWH Single Cell Genomics Core. After a 30-minute incubation at 4°C, cells were washed twice with Cell Staining Buffer and resuspended in 0.4% BSA/PBS. After performing a live cell count using Trypan blue exclusion method, cells were resuspended at 1,000 cells per microliter and a maximum of 15,000 cells were loaded into a Chromium Next GEM Chip G (10x Genomics). For samples with fewer than 15,000 live cells, all cells were loaded into the chip. cDNA and library generation were done according to the manufacturer's protocol. mRNA libraries were sequenced to an average of 50,000 reads per cell using Illumina Novaseq S4. CITE-seq antibody-derived tag (ADT) libraries were sequenced to an average of 5,000 reads per cell using Illumina Hi-Seq X Ten.

Single-cell CITE-seq gene expression and protein expression quantification
We quantified mRNA and antibody-derived tag (ADT) unique molecular identifier (UMI) counts using Cell Ranger v3.1.0. First, raw BCL files were demultiplexed using cellranger mkfastq with default parameters to generate FASTQ files. Then, these FASTQ files were aligned to the GRCh38 human reference genome using Cell Ranger v3.1.0. Gene and ADT reads were quantified simultaneously using cellranger count.

Quality control of single-cell CITE-seq data
We show each QC step in **Supplementary Fig. 1**. Specifically, we performed consistent QC to remove cells that expressed fewer than 500 genes or contained more than 20% of their total UMIs mapping to mitochondrial genes, resulting in 403,596 cells. Then, we performed sample-level QC and removed samples with a low percentage (< 40%) of cells passing QC. We

removed three lower-quality samples (processed on the same day) with less than 40% of cells passing QC compared to 71% for other good quality samples. In the end, we obtained 393,344 cells from 82 samples that passed QC.

We identified and removed doublets based on a combined strategy:

1. To detect doublets/multiplets based on gene count, we utilized the Scrublet[57] framework implemented in Python on each sample. We input the full raw, unnormalized UMI count data into the Scrublet() function with default parameters. We determined the doublet scores and the threshold for doublet detection by using the scrub_doublets() function with the following parameters: min_counts = 2, min_cells = 3, min_gene_variability_pctl = 85, and n_prin_comps = 30. Based on the distribution of modes of simulated doublet gene expression distributions, we set the threshold at 0.66. Based on this threshold, we identified 4.5% of cells as doublets.
2. Using protein expression, we trained an LDA (Linear Discriminant Analysis)-based classifier on non-doublet cells and then predicted the posterior probability of doublets using cell-type-specific antibodies (CD45, CD3, CD14, CD19, CD20, CD56, CD1C, PDPN, CD146), which improved the precision of doublet detection in a multimodal fashion. We obtained 314,011 cells after doublet detection.

To assess the accuracy of protein measurements in CITE-seq, we selected antibodies for surface markers of each cell-type lineage: T cells (CD45 and CD3D), NK cells (CD45, CD56, CD16, and IL17R), B cells and plasma cells (CD45 and CD19), macrophages (CD45 and CD14), classical dendritic cells (cDCs, CD1c), fibroblast (PDPN), mural cells (PDPN and CD146), and endothelial cells (CD146) (**Supplementary Table 2**). For flow cytometry, we used 13 antibodies (**Supplementary Table 10**). Flow cytometry data were collected on a BD FACSAria Fusion running BD FACSDiva 8.0.1 software and were analyzed using FlowJo 10.7.1. We measured the Pearson correlation between the per-donor proportion of cells in each gate across donors. We removed surface proteins with low expression overall.

mRNA feature normalization, selection, and scaling

Global: For each cell, we normalized the expression of each gene with log(1 + UMIs for gene/total UMIs in cell *10,000). Then, we selected the top 1,000 most highly variable genes in each sample based on a variance stabilizing transformation (VST)[58], which considers overall variance of the transcript per sample. We excluded cell cycle genes from “Seurat::cc.genes” for downstream analysis. We then pooled the most highly variable genes across all samples for a cell type into a data matrix and performed z-score scaling on each gene to have mean=0 and variance=1 across cells.

By cell type: We carried out the same normalization, feature selection, and scaling steps as described for the global analysis, but on only the cells of each given cell type.

Protein feature normalization, selection, and scaling

Global: For each cell, we normalized each protein with centered-log ratio (CLR): $\{ln(x_1/g(x)),...,ln(x_n/g(x))\}$, where x is a vector of protein counts[56]. For each feature, we then performed z-score scaling on each protein to have mean 0 and variance 1 across cells. To improve discrimination of signal and background in visualizations, we corrected for antibody background staining by fitting a Gaussian mixture model (with the normalmixEM function from the mixtools R package; k = 2, lambda = 0.5) to the CLR-normalized expression of each protein in each cell type. Then we calculated the mean of the first (lower) Gaussian in each cell type, identified the lowest mean across cell types, and subtracted this value—representing background—from all cells' expression of the protein (with a lower bound of 0 for any values that would otherwise become negative).

To select variable proteins, we measured Kullback-Leibler (KL) divergence for each protein by comparing the distribution of cells with normalized expression above the 75th percentile for that protein across broad cell-type clusters, versus the distribution of all cells across broad cell-type clusters. For each feature, we then performed z-score scaling on each protein to have mean = 0 and variance = 1 across cells. We used a KL-divergence threshold of 0.3.

By cell type: We carried out the same normalization as described for global analysis, but only on the cells of each given cell type. For T and B/plasma cells only, we conducted protein feature selection and scaling as described for global analysis. We removed proteins expressed in < 1% of cells and selected variable proteins based on KL divergence (computed as described above except using the 85th percentile to define the distribution of protein-expressing cells). Proteins with KL divergence greater than or equal to 0.025 were considered variable.

A unimodal dimensionality reduction strategy for single-cell gene expression
For cell-type-specific analysis of myeloid cells, fibroblasts/mural cells, endothelial cells, and natural killer cells, we used a unimodal pipeline to reduce the dimensionality of the data based on mRNA expression. For each cell type, we used truncated principal component analysis (PCA) as implemented in the prcomp_irlba function from the irlba R package[59] and calculated 20 principal components (PCs) based on the scaled mRNA data. We then corrected sample-driven batch effects with the HarmonyMatrix function from the harmony R package[60] with parameters as specified in **Supplementary Table 3** and projected the cells into two dimensions with UMAP[61].

A multi-modal dimensionality reduction strategy for CITE-seq data
For global analysis of all cell types and cell-type-specific analysis of T and B/plasma cells, we used a multi-modal pipeline to integrate mRNA and surface protein expression from the same cells and project the cells into a low dimensional embedding informed by both modalities[62]. After scaling the protein features so that their total variance was equal to the total variance of the mRNA features, we used canonical correlation analysis (CCA) as implemented in the cc function from the CCA R package to calculate canonical variates (CVs)[63] based on the scaled mRNA and surface protein data. These are projections of cells onto axes defined by maximally correlated linear combinations of genes and surface proteins that capture the greatest amount of shared variance. For further analysis, we selected the top 20 CVs with highest canonical

correlations, as defined in the mRNA space. We then corrected sample-driven batch effects with the HarmonyMatrix function from the harmony R package[60] with default parameters and projected the cells into two dimensions with UMAP[61].

Graph-based clustering, differential gene expression, and cell type annotation

We then constructed shared nearest neighbor graphs derived from the top 20 CVs/PCs and applied graph-based Louvain clustering[64] at various resolution levels (0.2, 0.4, 0.6, 0.8, 1.0). We selected optimized resolution values for each cell type (1.2 for T cells, 0.8 for NK cells, 0.6 for myeloid cells, 0.6 for B cells, 0.6 for stromal cells, 0.3 for endothelial cells) to gain the biological interpretations that made the most sense. We incorporated the number of variable genes chosen per sample and parameters for each cell type's analytical pipeline in **Supplementary Table 3**. In the end, we identified 24 T cell clusters (94,046 cells), 9 B cell clusters (30,691 cells), 14 NK clusters (8,495 cells), 15 myeloid clusters (76,181 cells), 5 endothelial clusters (25,043 cells), and 10 stromal cell clusters (79,555 cells), for a total of 77 clusters.

For each major cell type, we identified differentially expressed mRNA features and surface proteins by comparing cells from one cluster with all the other cells. We collapsed single-cell mRNA and protein expression profiles into pseudo-bulk count matrices by summing the raw UMI counts for each gene or surface protein across all cells from the same donor and cluster. For mRNA, we tested all mRNA features that were detected in more than 100 cells per individual with non-zero UMI counts. For each feature, we normalized counts in each pseudo-bulk sample into counts per million (CPM). Using linear models, we estimated the effect of each cluster for each feature on pseudo-bulk expression accounting for effects from the donor and the number of UMIs for each pseudo-bulk sample. Next, we used likelihood ratio tests (LRT) between two models: one that has the cluster variable, and another that doesn't have the cluster variable. Finally, we selected a feature to be a cluster marker if it had a fold change greater than 2 and p-value less than FDR 5%, which is $p < 0.05/(\text{number of tested genes} \times \text{number of clusters})$. We repeated a similar analytical pipeline of normalization and scaling, feature selection, multi-modal dimensionality reduction, clustering, and differential expression analysis for T cells ($p < 1.5\text{x}10^{-6}$), B cells and plasma cells ($p < 1.9\text{x}10^{-6}$), NK cells ($p < 1.6\text{x}10^{-6}$), myeloid cells ($p < 1.8\text{x}10^{-8}$), stromal cells ($p < 4.3\text{x}10^{-7}$), and endothelial cells ($p < 1.2\text{x}10^{-6}$), respectively. Furthermore, we annotated each cell-type cluster based on literature. We present cluster-specific marker genes and relative statistics in **Supplementary Table 5**. We visualized our data and results at our cell browser website https://immunogenomics.io/ampra2/ using Cell Guide [65].

Building and mapping to global and cell-type-specific references

We used the buildReferenceFromHarmonyObj() function from the Symphony[66] package to build integrated reference atlases for the global and cell-type specific atlases from the Harmony objects. To find concordance between cell types from our previous study[8] and this study, we used the Symphony mapQuery() function to map the 5,265 scRNA-seq query cells from Zhang *et al*, 2019 onto the global and respective cell-type reference atlases. We predicted reference cell types and states for the query cells using the knnPredict() function with k = 5. For the cell-type specific mapping, we excluded reference dendritic cells or mural cells because they were absent in the query. Note that because the gene expression matrices for the reference (this

study) and query[8] datasets were generated using different versions of Gencode (version 19 vs. version 29, respectively), certain genes were named differently between the two datasets (e.g. *IL8* and *CXCL8* are synonyms for the same gene ENSG00000169429). Because the mapping procedure uses overlapping gene names between reference and query, we “synced” the query gene names to the version 29 names using the shared Ensembl IDs (which do not change between Gencode versions) using the Gencode .gtf files. This converted 9,663 query gene names, and the synced expression matrix was used as input to mapping.

Identification of CTAPs based on single-cell cell-type abundance
We identified six cell-type abundance phenotypes (CTAPs) based on hierarchical clustering on cell-type abundances for each CITE-seq patient sample. The differences across CTAPs are also reflected in the PCA space. We named each CTAP based on the cell types whose average proportions were higher among samples in the CTAP compared to their average across all samples (**Supplementary Table 4**). To assess the stability of CTAPs, 1) We first bootstrapped the patient samples and clustered the resampled dataset, 2) For every original CTAP subgroup, we found the most similar cluster (based on Jaccard similarity) in each resampled clustering and recorded that value, giving us the maximum Jaccard similarity coefficient for each CTAP. The Jaccard similarity coefficient can be a value between 0 and 1, where 1 indicates complete overlap and 0 indicates no overlap between two sets of the clustering results, 3) We repeated the above two steps 1e4 times and calculated the mean Jaccard similarity coefficient. We performed this process on different possible numbers of patient subgroups ranging from 2 to 10, and evaluated the statistical stability retaining in-group similarity. We selected six clusters as CTAPs because they gave us relatively high stability (mean Jaccard similarity coefficient=0.727) and also high granularity of biologically meaningful interpretations.

Covarying neighborhood analysis (CNA) to identify cell populations associated with patient CTAP membership
We evaluated whether the global RA CTAPs are associated with changes in the relative abundances of cell states within each of our six major cell types, which would indicate that these CTAP groupings reflect both coarse (relative abundance of major cell types) and fine-scale heterogeneity in synovial tissue composition.

For each major cell type, we used CNA[67] to associate sample-level attributes to the abundances of cell states within that cell type. CNA defines many small cell neighborhoods in the batch-corrected low-dimensional space and stores that fractional abundance of cells from each sample in each neighborhood in a neighborhood abundance matrix (NAM). By decomposing the NAM with principal component analysis, CNA defines NAM-PCs within each cell type that capture axes of heterogeneity defined by groups of neighborhoods whose abundances vary in a coordinated manner. Here, we use CNA to test for associations between sample-level clinical characteristics and the abundance of covarying neighborhood groups. For associations with histologic metrics such as histology density and aggregate scores, we only used samples that passed histology-level QC grades (Grade A and B). We also use CNA to identify neighborhoods that are associated with one CTAP compared to other CTAPs.

To perform CNA, we used the tl.association() function in the cna Python package with default parameters and top four NAM-PCs as inputs, while controlling for the "age", "sex", and "number of cells per sample" as covariates. As CNA utilizes a permutation test, we determined a significant association based on a global permutation $p < 0.05$. For visualization of local associations, which indicate the particular neighborhoods driving a found global association, we used the 5% FDR threshold from CNA to determine which neighborhoods featured a locally significant correlation. In violin plots, we plotted this threshold as dotted lines. In UMAP plots, we colored neighborhoods that pass local significance based on the intensity of their correlation, with red indicating a higher positive correlation, while we colored neighborhoods that did not attain local significance as grey. We used a modified version of CNA, available on Github, which included the following features: 1) scaling the variance per neighborhood within the NAM inversely to the sample size of the source sample for that neighborhood's anchor cell such that total variance across all neighborhoods anchored on cells from the same sample sums to 1, and 2) the addition of a pseudo-count, a small number that was added to each entry in the NAM. Using CNA, we tested associations of cell neighborhoods that are associated with histology, ultrasound, clinical metrics, and also each CTAP group. The statistics are in **Supplementary Table 7.**

Modeling histologic, clinical, and demographic characteristics using CTAPs

We used linear mixed modeling to model each histologic parameter and clinical demographic variable using single-cell CTAPs. Only samples that passed histology-level QC (Grade A and B) were included to seek an association between molecular-level categories and histologic metrics. Taking histologic density Y as an example, we fitted a mixed-effect model for each CTAP with the number of cells per sample as a cell-level fixed effect, age and sex as demographical level fixed effects, and clinical collection site as a random effect covariate:

Full model: $Y_i = 0 + \sum_j \beta_j X_{i,j} + \beta_{age} X_{i,age} + \beta_{sex} X_{i,sex} + \beta_{tech} X_{i,tech} + (1|site)$,

Null model: $Y_i = 0 + \beta_{age} X_{i,age} + \beta_{sex} X_{i,sex} + \beta_{tech} X_{i,tech} + (1|site)$

where β_j is the effect size for each CTAP j for sample i, β_{age} is a vector of age values and β_{sex} is a vector of sex values, β_{tech} is a vector containing a technical covariate that captures the number of cells for each single sample, $X_{i,j}$ is the one-hot encoded variable for sample i in CTAP j as appropriate, and $(1|site)$ is the random effect for clinical collection sites. Thus, we used the full model to calculate the corrected values of CTAPs accounting for these technical, cell-level, and donor-level covariates. For modeling age and disease duration, we used a similar model but we removed the age fixed effect from both the full and null model. We obtained percent of variance explained by the CTAPs only by subtracting the variance explained by the null model from the variance explained by the full model. ANOVA p-value was also calculated. The R package lme4 was used for the mixed effect modeling[68].

Classifying flow cytometry samples into RA CTAPs

We provided a proof-of-concept framework to assign RA samples processed by other data modalities (e.g., flow cytometry) to the RA CTAPs generated from single-cell technology. Specifically for **Fig. 5c**, 1) we quantified the major cell type abundances in a sample using flow

cytometry based on cell type markers derived from the single-cell technology, then 2) we mapped each flow sample to the principal component space generated from the CITE-seq cell-type abundances based on the same features. Here, the features are T, B, myeloid, stromal, endothelial, and NK cell canonical markers. Now that each flow sample has a loading in the original single-cell abundance space, 3) we built a Mahalanobis-distance-based nearest-neighbor classifier to measure the distance of a flow sample to each of the CTAP centroids. We use Mahalanobis distance to handle the covariance, because our CTAP clusters in PC space are elliptical shaped covariances rather than circular shapes. 4) For each flow sample, we assigned a CTAP label based on which CTAP centroid had the smallest Mahalanobis distance. We calculated the accuracy of our classifications based on a subset (n = 15) of RA synovial tissues that were analyzed by both single-cell CITE-seq and flow cytometry.

Pseudo-bulk analysis of soluble immune factors
To perform the pseudo-bulk analysis of soluble immune factors, we summed and normalized the gene counts of the chosen 55 soluble immune mediators from KEGG gene set M9809[69] across all cells within a sample, resulting in a soluble immune mediator count by sample matrix. We then performed principal component analysis (PCA) to obtain the resulting principal component embeddings shown in **Extended Data Fig. 1g**. Samples in the figure were colored by their original CTAP label defined by cell types.

T cell functional assays
T cells were isolated from cryopreserved RA donor PBMCs (n = 3) by thawing and subsequently staining directly with LIVE/DEAD Fixable Aqua Dead Cell Stain (1:1000, L34957, Invitrogen), anti-CD4 APC (1:100, RPA-T4, 300537), anti-CD8A BV711 (1:100, RPA-T8, 301044), anti-CD3 APC-Cy7 (1:100, OKT3, 317342), anti-CD14 FITC (1:100, HCD14, 325604), anti-CD45RA BV605 (1:100, HI100, 304134), anti-CCR7 PE-Cy7 (1:100, G043H7, 353226) and anti-PD-1 BV421 (1:100, EH12.2H7, 329920), all from Biolegend, and sorted with a 5-Laser BD FACS-Aria Fusion cell sorter. T cells were sorted as follows: $CD14^-CD3^+CD4^+CD8^-CD45RA^-PD\text{-}1^{hi}$ (T_{PH}+T_{FH}), $CD14^-CD3^+CD4^+CD8^-CD45RA^-PD\text{-}1^-$ ($PD\text{-}1^-$ Memory CD4), $CD14^-CD3^+CD4^-CD8^+CD45RA^-$ (Memory CD8), $CD14^-CD3^+CD4^-CD8^+CD45RA^+CCR7^-$ (TEMRA CD8). Sorted T cell populations from RA donors were either plated immediately together with allogeneic memory B cells to assess B cells activation or rested overnight at 37°C for subsequent quantitation of cytotoxic activity.

For the B cell assays, B cells were isolated from thawed cryopreserved PBMCs from leukoreduction collars (n = 3) using a B cell isolation kit (MACS; Miltenyi) and subsequently sorted for memory B cells using LIVE/DEAD Fixable Aqua Dead Cell Stain (1:1000, L34957, Invitrogen), anti-CD19 PE (1:100, HIB19, 302208), anti-CD27 BV421 (1:100, M-T271, 356418), anti-CD3 FITC (1:100, OKT3, 317306) and anti-CD14 APC (1:100, HCD14, 325608) all from Biolegend, using a 5-Laser BD FACS-Aria Fusion cell sorter. Memory B cells from control donors and RA T cells were plated at a ratio of 10:1 and stimulated with staphylococcal enterotoxin B (SEB; 1µg/ml; Toxin Technology) for 5 days before analysis by flow cytometry. Cells were stained with anti-CD3 FITC (1:100, OKT3, 317306), anti-CD20 BV605 (1:100, 2H7, 302334), anti-CD19 APC-Cy7 (1:100, HIB19, 302218), anti-CD27 PE-Cy7 (1:100, M-T271,

356412), anti-CD38 BV785 (1:100, HIT2, 303530), anti-CD11c PE (1:50, Bu15, 337206), and anti-CD21 PerCP-Cy5.5 (1:100, Bu32, 354908), all from Biolegend, and analyzed on a BD Fortessa analyzer.

Cytotoxic capacity of RA T cells was measured with a novel cytotoxicity assay using CD32-expressing L cells loaded with an agonist anti-CD3 antibody as target cells. T cells co-cultured with target cells then become stimulated resulting in target cell death measurable by AnnexinV positivity[70]. CD32-expressing L cells were incubated with an agonist anti-CD3 antibody (OKT3; 50 ug/mL, BioXcell) on ice for 30 min and then plated with the sorted and rested RA T cells at a ratio of 1:5. Following a 3.5-hour incubation, the cells were removed from culture and stained with AnnexinV APC and 7-AAD (5 uL/100uL each, Biolegend) and analyzed immediately on a BD Canto II analyzer.

***In vitro* modeling of macrophage phenotypes in CTAP-M and CTAP-TM**

Monocytes and $CD8^+$ T cells were isolated from human donor PBMCs using anti-CD14 and anti-CD8 magnetic microbeads (Miltenyi Biotec), respectively. Monocytes were isolated from three separate donors while the T cells were isolated from one of two donors. All cultures contained RPMI++ (RPMI medium (Corning), 10% fetal bovine serum (HyClone) and 1% L-glutamine (Gibco)). To generate a homogenous monocyte population, $1x10^6$ cells/mL CD14+ cells were plated in a 24-well plate for 24 hours in RPMI++ with 50ng/mL M-CSF (Biolegend). To generate activated T cell-conditioned media, $CD8^+$ T cells were rested overnight in RPMI++ and then activated for 6 hours using 1:1 cell:bead ratio of anti-CD3/anti-CD28 antibody-coated beads (Dynabeads, Gibco). Synovial fibroblast lines were generated from mildly inflamed rheumatoid arthritis arthroplasty tissue. To model CTAP-M, $6x10^5$ monocytes were input into the upper chamber of a 24-well transwell (Celltreat) with 1mL RPMI++, containing 50ng/mL M-CSF and 20ng/mL TGF-β (Biolegend), while $7.5x10^4$ fibroblasts at passage 4 were plated in the lower chamber for 72 hours. The M-CSF-alone condition was prepared similarly, but with no cells in the bottom chamber. To model CTAP-TM, $6x10^5$ macrophages were placed into the upper chamber of a 24-well transwell with 1mL T cell conditioned RPMI++ containing 50ng/mL M-CSF for 12 hours. The bottom chamber of the transwell was empty. All cells were collected in RLT with 1% beta-mercaptoethanol for bulk RNA-sequencing and purified RNA was prepared using the RNAeasy kit (Qiagen). Libraries were generated using the Illumina Stranded mRNA Prep, Ligation kit (20040534 v02). The sequencing was performed on an Illumina NovaSeq 6000 - S4 Flow Cell (PE 2x100 cycles) at a depth of 40M reads per sample.

Bulk RNA-seq analysis of monocyte differentiation experiments using linear discriminant analysis

To perform the bulk RNA-seq analysis for the monocyte differentiation experiments, bulk FASTQ files were processed with default FastQC parameters, and then converted into a count matrix with default STAR alignment parameters using GRCh38 as the reference genome. We then inputted the bulk RNA-seq count matrix and our full CITE-seq count matrix into independent Seurat objects and log-normalized the count matrices with scale factors of 1e7 and 1e4 for the bulk RNA-seq and CITE-seq matrices respectively. We then subsetted the CITE-seq

count matrix to only include cells in the monocyte and macrophage clusters (myeloid clusters 0, 1, 2, 3, 4, 5, 6, and 7).

To select genes for the linear discriminant analysis (LDA), we utilized the “wilcoxauc” function from the “presto” package to find the top 500 AUC genes for each CITE-seq cluster, resulting in 3,001 unique genes. We then thresholded these genes such that we only kept genes with a mean > 0.25 expression in the CITE-seq dataset, resulting in 1,741 genes. Finally, these remaining genes were intersected with the genes measured in the bulk RNA-seq dataset, resulting in 1,713 unique genes. Both count matrices were then subsetted to include only these 1,713 genes, and then each gene was scaled to have mean = 0 and variance = 1.

To perform LDA, we utilized the “lda” function from the “MASS” package with the following parameters: the formula was “cluster ~ .”, the data was the CITE-seq scaled count matrix, and the prior was a uniform value of 0.125 (1 / (# of clusters) = 1 / 8). To predict posterior probabilities for the bulk RNA-seq data, we used the “predict” function with the following parameters: the object was the previously fitted LDA, and the new data was the bulk RNA-seq count matrix. Since clusters 5 and 7 had posterior probabilities equal to zero, the pie charts only include the posterior probabilities of clusters 0, 1, 2, 3, 4, and 6

Associating imputed *HLA-DRB1* alleles with RA CTAPs

As described in Kang, et al., we genotyped donors in the AMP RA/SLE Network across three batches using the Illumina Multi-Ethnic Genotyping Array[71]. We used PLINK v1.90 to QC the data, including removing variants with high missing rates (--geno 0.01), low MAF (--maf 0.01), or violating Hardy-Weinberg equilibrium (--hwe 1e-6)[72]. After merging batches and applying additional filters, 820,019 genome-wide variants (10,159 in MHC) and 788 individuals passed QC, of which 78 individuals had paired CITE-seq data. For HLA imputation, we used SNP2HLA and a multi-ethnic reference panel (v2)[73–75]. We imputed MHC SNPs, classical HLA alleles at one- and two-field resolution (HLA-A, -B, -C, -DPA1, -DPB1, -DQA1, -DQB1, and -DRB1), and HLA amino acid positions. For the *HLA-DRB1* genotype analysis, we used the imputed dosage (ranges from 0 to 2) for each two-field HLA-DRB1 allele. Then, to calculate the HLA RA risk score for each individual, we multiplied each of their variants' dosage by the RA risk odds ratio from ACPA$^+$ RA GWAS summary statistics[76] and summed them. We incorporated 46 overlapped alleles in our analyses. We associated HLA imputation RA risk scores with CTAPs using mixed effects linear modeling controlling age, sex, and site.

Cross-cell-type communication analysis

To provide a systematic evaluation of cell-cell cross-talk, we used CellChat[77]. We queried more than 2,000 ligand-receptor pairs collected from CellChatDB including secreted signaling, ECM-receptor, and cell-cell contact using default parameters.

Pre-processing PEAC and R4RA bulk RNA-seq data, and converting our CITE-seq data into pseudo-bulk data

To pre-process PEAC bulk samples, metadata and FASTQ files were downloaded from the ArrayExpress link provided by Lewis et al. Cell Reports 2019 (Accession Number: E-MTAB-

6141). FASTQ files were analyzed with default FastQC parameters, and then converted into a count matrix with default STAR alignment parameters using GRCh38 as the reference genome, resulting in a gene count by sample matrix. Furthermore, seven unpublished bulk samples from this cohort that derive from the same patient and biopsy procedure as seven CITE-seq dataset samples were added in order to validate the concordance of our CTAP classification algorithm-based label with a sample's cell type proportion-based CTAP label. These unpublished samples were processed, sequenced, and aligned as previously described[6].

To pre-process R4RA bulk samples, metadata and FASTQ files for each of the 178 samples from 133 patients were downloaded from the ArrayExpress link provided by Rivellese, Surace, *et al*. Nature Medicine 2022 (Accession Number: E-MTAB-11611). FASTQ files were analyzed with default FastQC parameters, and then converted into a count matrix with default STAR alignment parameters, using GRCh38 as the reference genome, resulting in a gene count by sample matrix.

To pre-process our CITE-seq data into pseudo-bulk samples, each gene's total counts across all cells within a sample were summed. We performed this count summation for each of the 70 RA samples in the CITE-seq data. Overall, this resulted in a gene count by sample matrix (70 samples).

Assigning CTAP labels to bulk RNA-seq samples with CCA and a k-nearest neighbor classifier

To assign CTAP labels to PEAC bulk RNA-seq samples, the genes in the PEAC and pseudo-bulk AMP gene count by sample matrices were filtered within each dataset respectively such that they retained only genes with non-zero expression in every bulk sample. After filtering each gene count by sample matrix, we took the intersection of both dataset's genes, and filtered the matrices such that they retained only intersecting genes. Each gene count by sample matrix was then inserted into an individual Seurat object. Each matrix was then log-normalized with the "NormalizeData" function, with a scaling factor of 1e7. Variablef features were identified with the "vst" method of the "FindVariableFeatures" function (nfeatures = 4500). The variable features of each dataset were then intersected, the normalized data was retrieved from each Seurat object, and each gene was scaled such that the mean and variance of a gene across samples were 0 and 1 respectively. After pre-processing, the resulting gene count by sample matrices consisted of scaled gene expression.

To map our CITE-seq dataset and the PEAC dataset into the same shared embedding, we input each scaled gene count by sample matrix into the "cc" function from the "CCA" package in order to obtain a CCA list. The loadings for each dataset contain embeddings for each sample into the shared CCA space. Loadings from the CCA list were extracted from the "scores" slot. Loadings for each dataset (corr.X.xscores and corr.Y.xscores) were then concatenated into the same dataframe. To assign CTAP labels to a bulk PEAC sample, we used a k-nearest neighbor classifier. Based on the top 8 CVs—because the correlation explained after the first 8 CVs noticeably decreased—we used Seurat's FindNeighbors function to identify the 5 nearest CITE-

seq pseudo-bulk samples for each PEAC bulk sample, and then classified the PEAC bulk sample based on a majority vote of the CTAP labels of its CITE-seq pseudo-bulk neighbors.

To map our CITE-seq dataset and the R4RA dataset into the same embedding, we performed the same steps as previously described for the PEAC dataset. Note that because some of the bulk R4RA samples were from multi-visit patients (biopsied after 16 weeks of treatment), only the baseline week 0 samples were used for the logistic regression analyses that utilizes pre-treatment CTAP assignment to predict responder status. Likewise, for the analyses that compare pre-treatment CTAP labels with post-treatment CTAP labels, only samples from multi-visit patients were used.

Linking RA causal genes to CTAP-associated cell states by cell type

We first identified likely causal genes from a recent genetic study of RA with >250,000 individuals from five ancestry groups[45] (**Supplementary Table 11**). We identified RA genes as those that either (1) had a RA risk allele that conferred a nonsynonymous change, (2) had an eQTL in GTEx or Blueprint that colocalized with an RA risk allele, (3) had a high probability fine-mapped causal variant (posterior probability >0.5) within a promoter, intron or exon or (4) had a rare nonsynonymous risk allele implicated through a recent sequencing study[46]. Second, we examined the genes expressed by more than 5% of the cells in a given cell-type (e.g. B cells). For genes expressed in a cell type, we assessed if loadings from CNA for specific CTAPs were correlated with expression. A positive correlation indicates that a cell state expanded within a CTAP specifically expresses an RA risk gene (**Fig. 5d**). Lastly, we defined significance by comparing to genes with matched expression within the same cell type (one-tailed $p < 0.05$). Taking gene *IL6R* as an illustration, we identified a gene list L (n = 192) whose genes have similar expression levels with *IL6R* in T cells (**Supplementary Fig. 13b**). We created a distribution of the Spearman correlations between genes in list L and the CTAP-TB associated T cell neighborhood loadings. We determined significance by comparing the number of genes with expression patterns significantly correlated with CTAP-associated cell states in one or more cell types to the number of such genes that we expect by chance (median = 34 genes) through an 1e3 permutation test (permutation $p < 0.01$).

Supplementary Figure 1

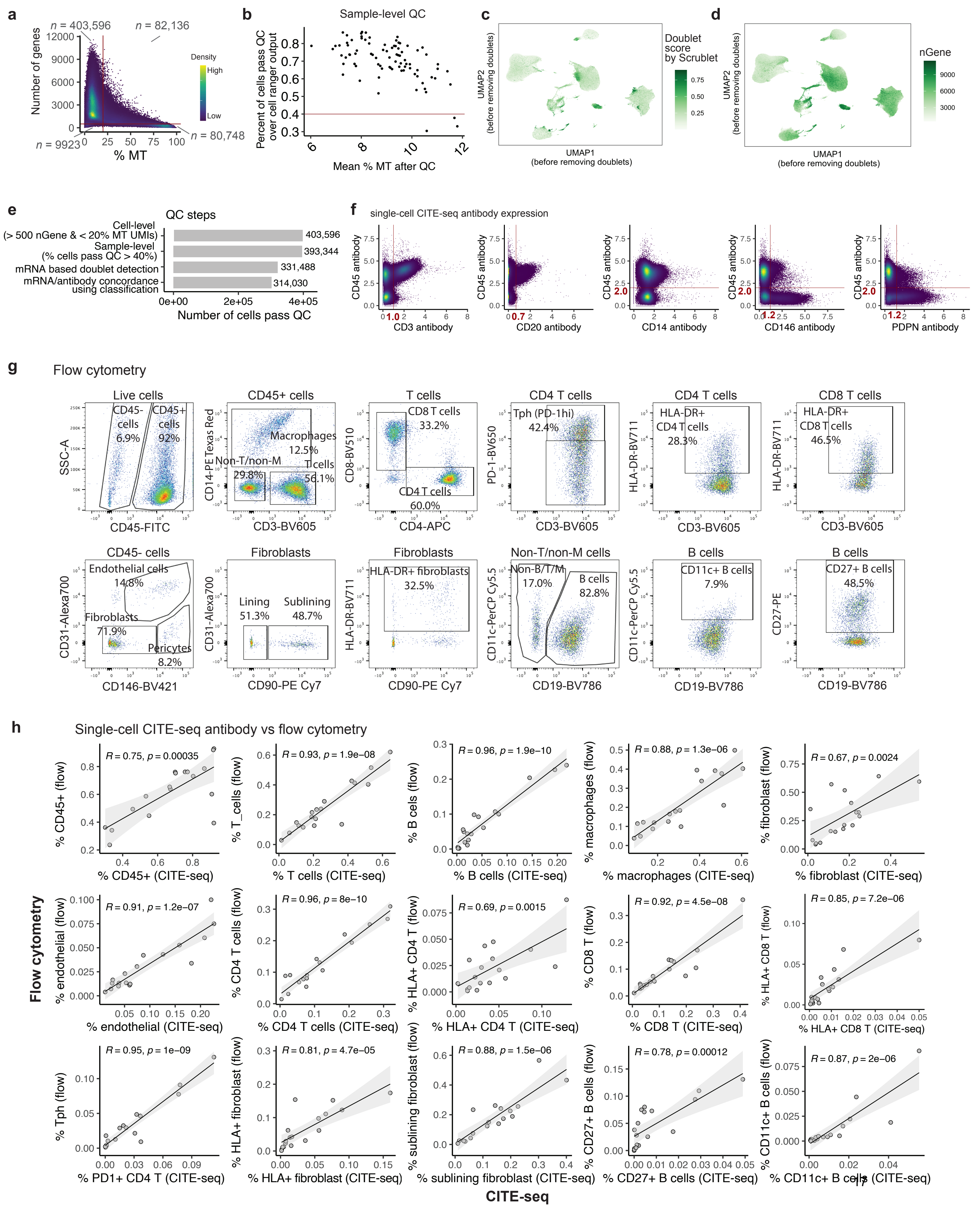

Supplementary Fig. 1. Detailed single-cell CITE-seq quality control. **a**. Quality of the cells based on number of genes detected and percent mitochondrial UMIs (%MT), **b**. Percentage of good quality cells for sample-level QC, **c**. Doublet detection using Scrublet, **d**. UMAP of the number of genes detected, **e,** Number of cells remaining after each step of QC, **f**. Distributions of cell type lineage antibody staining from CITE-seq determine percentage of major cell types based on the thresholds (red line) including % $CD45^+$ cells, % T cells based on $CD3^+$, % B cells based on $CD20^+$, % macrophages based on $CD14^+$, % endothelial cells based on $CD146^+$, and % fibroblasts based on $PDPN^+$, **g**. Representative gating of flow cytometry data to quantify selected synovial cell populations, **h**. The proportion of cells within 15 lineage gates in single-cell CITE-seq antibody staining with an analogous gating schema for flow cytometry (N=18, median Pearson r=0.88). For flow gating, we determined % $CD45^+$ based on $CD45^+$ over all live cells, % T cells based on $CD45^+CD3^+$ over all live cells, % B cells based on $CD45^+CD3^-CD14^-CD20^+$, % macrophages based on $CD45^+CD14^+$, % fibroblasts based on $CD45^-CD146^-CD31^-$, % endothelial cells based on $CD45^-CD146^+CD31^+$, % $CD4^+$ T cells based on $CD45^+CD3^+CD4^+$, % HLA^+ $CD4^+$ T cells based on $CD45^+CD3^+CD4^+HLA\text{-}DR^+$, % $CD8^+$ T cells based on $CD45^+CD3^+CD8^+$, % HLA^+ $CD8^+$ T cells based on $CD45^+CD3^+CD8^+HLA\text{-}DR^+$, % $PD1^+$ $CD4^+$ T cells based on $CD45^+CD3^+CD4^+PD1^+$, % HLA^+ fibroblasts based on $CD45^-CD146^-CD31^-HLA^+$, % sublining fibroblast based on $CD45^-CD146^-CD31^-CD90^+$, % $CD27^+$ B cells based on $CD45^+CD3^-CD14^-CD20^+CD27^+$, and % $CD11c^+$ B cells based on $CD45^+CD3^-CD14^-CD20^+CD11c^+$, respectively. R is the Pearson correlation coefficient and p-values are from two-sided t-tests. Shaded regions represent 95% confidence intervals.

Supplementary Figure 2

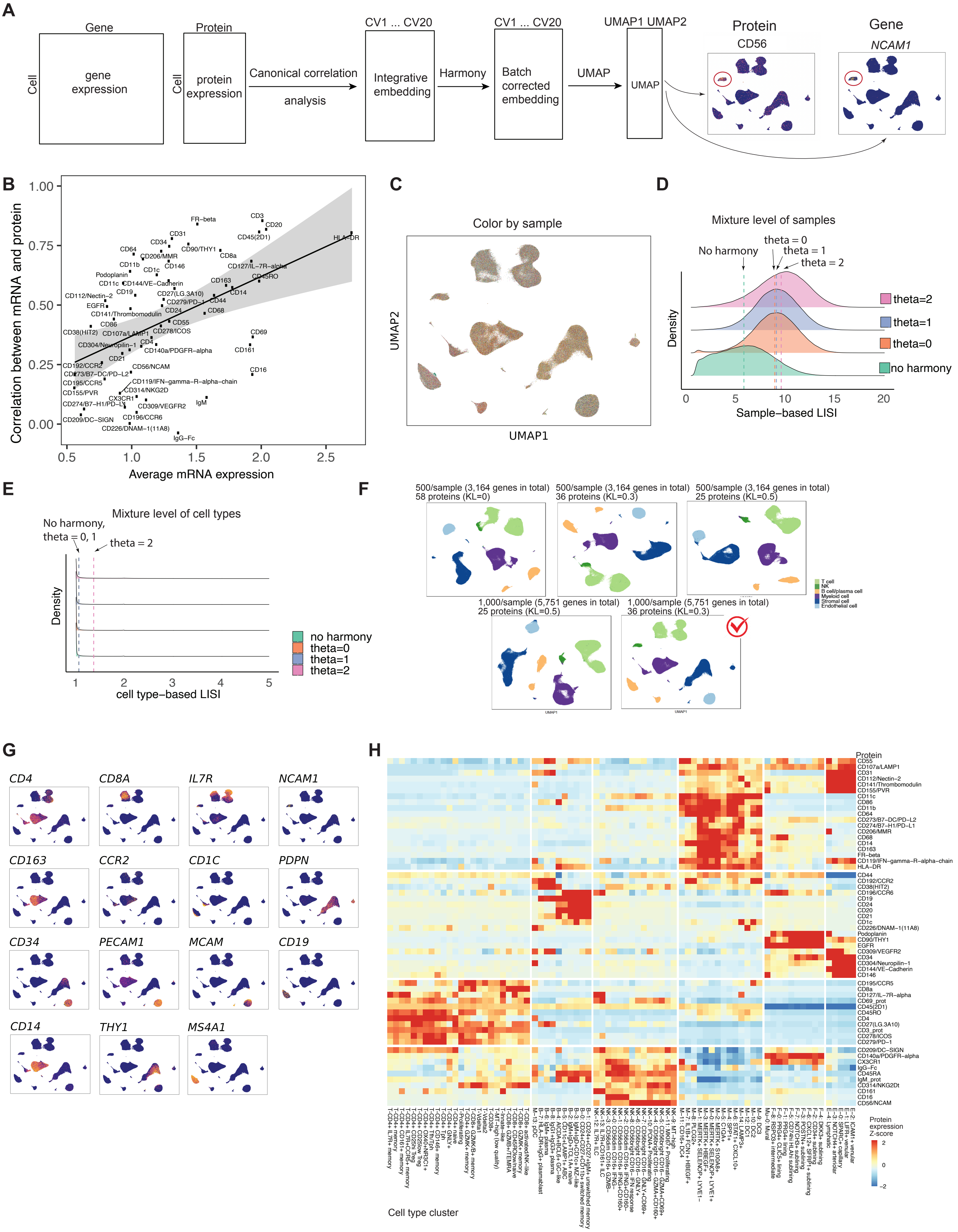

Supplementary Fig. 2. Single-cell CITE-seq integrative analysis. **a**. CCA-based pipeline for integrating mRNA and protein expressions, **b**. Concordance between average mRNA expression and the correlations of corresponding protein and mRNA expression. Black line represents the linear best fit line and the shaded region represents the 95% confidence interval, **c**. Sample sources (n=82) in the UMAP space, paired with sensitivity analyses of Harmony parameters based on LISI scores to measure mixture levels on **d**. samples and **e**. cell types, **f**. The effect of varying the selected number of antibodies based on each antibody's specificity: KL divergence equals 0.5 (25 proteins), 0.3 (36 proteins), and 0 (58 proteins), while also varying the number of highly variable genes used: 500/sample (3,164 genes in total) and 1,000/sample (5,751 genes in total) on the mRNA and protein integrative analysis. We used the top 1,000 most variable genes per sample and 36 most specific proteins because it best recovered major cell types and more clearly identified rare cell types, **g**. Gene expression of cell-type lineage signatures, **h**. Pseudo-bulk expression of surface proteins in each cluster. Expression levels are based on pseudo-bulk log2(CPM)-normalized values, scaled across clusters for each marker.

Supplementary Figure 3

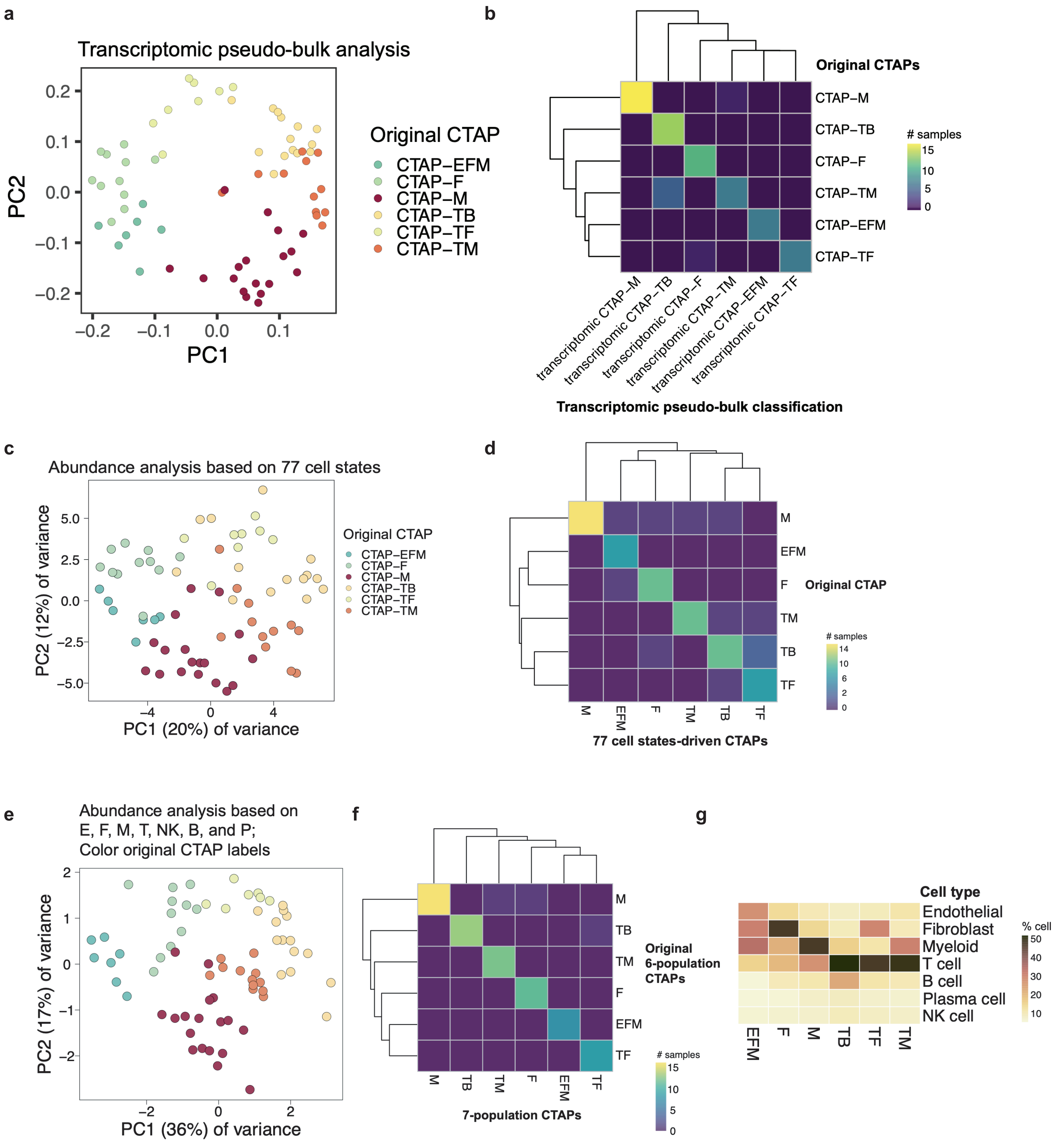

Supplementary Fig. 3. Alternative cluster schemes largely reproduce the CTAP classifications. a, PCA of the pseudo-bulk analysis for the full transcriptomes of the 70 RA samples, with samples colored by original CTAP classification. **b**, Heatmap visualizing concordance of the pseudo-bulk classification (columns) and original CTAP classification (rows) for each sample, **c**, PCA of the abundance analysis using the fine-grained cell states for the 70 RA samples, with samples colored by original CTAP classification. **d**, Heatmap visualizing concordance of the fine-grained cell states-driven classification (columns) and original CTAP classification (rows) for each sample, **e**, PCA of the abundance analysis using 7 cell populations including fibroblasts, endothelial cells, myeloid cells, T cells, NK cells, B cells, and plasma cells for the 70 RA samples, **f**, Heatmap visualizing concordance of the 7-cell population-driven classification (columns) and original CTAP classification (rows) for each sample. **g**, Heatmap depicting average proportions of each of the seven major cell types among samples in each of the original CTAPs.

Supplementary Fig. 4 is provided separately.

Supplementary Fig. 4. Representative histology images for each CTAP. Representative fragments from patients in each CTAP, showing composite and individual staining of each marker in the lymphocyte panel (**a, c, e, g, i, k**) or stromal cell panel (**b, d, f, h, j, l**). A total of 150 fragments from 36 individuals (mean 4.2 fragments per individual, range 2-9 fragments per individual) were stained in batches and analyzed as a single cohort. A high-resolution version of this figure is available at https://doi.org/10.5281/zenodo.8364277.

Supplementary Figure 5

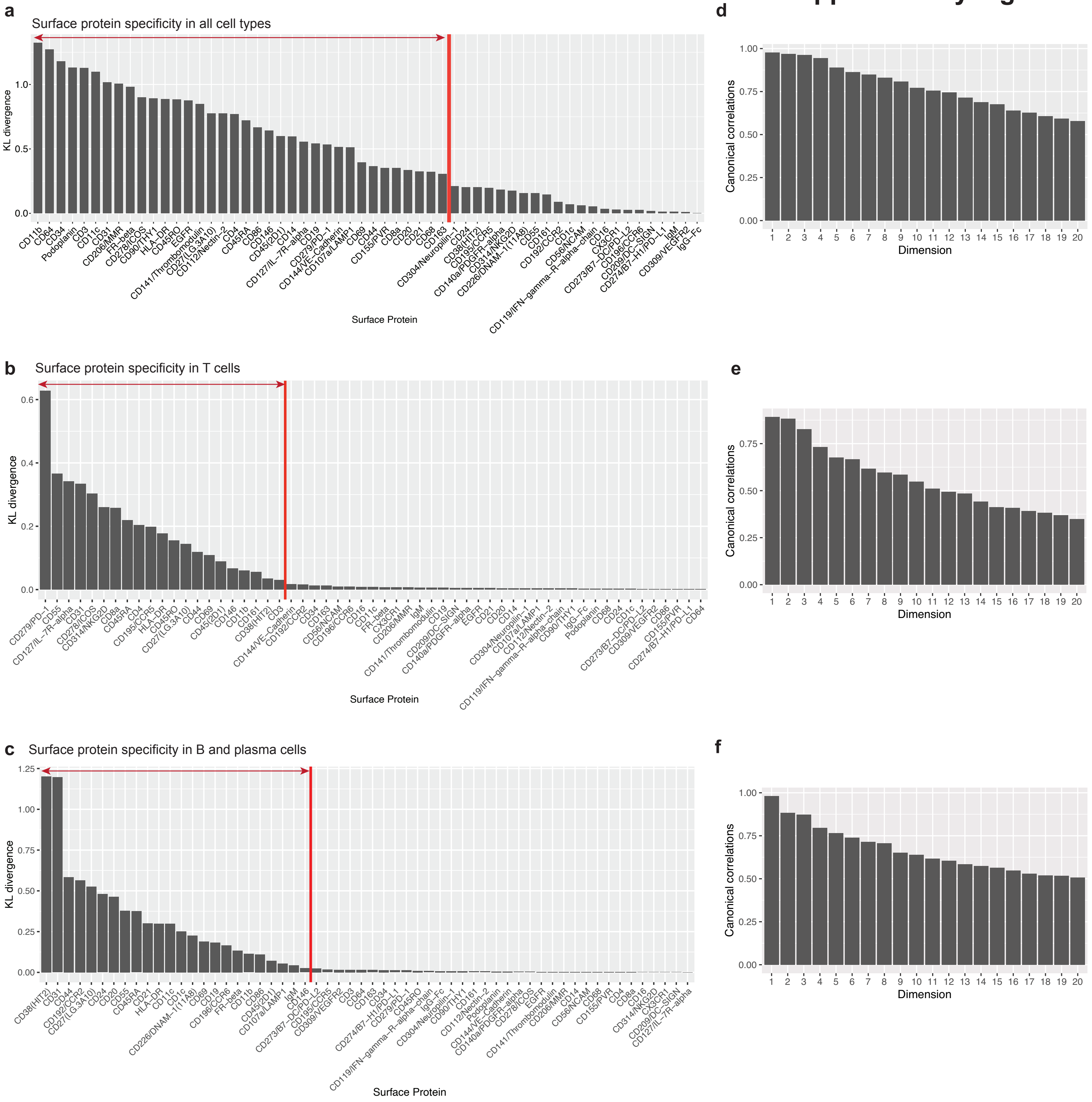

Supplementary Fig. 5 Surface protein specificity and selection for integrative analysis. Kullback-Leibler divergence measures the specificity of each protein across **a,** all cells, **b,** T cells, and **c,** B/plasma cells. Proteins to the left of the red line were chosen for the CCA integration of each cell type. Canonical correlations for each of the top 20 canonical variates (CVs) from canonical correlation analysis of **d,** all cells, **e,** T cells and **f,** B/plasma cells, respectively.

Supplementary Figure 6

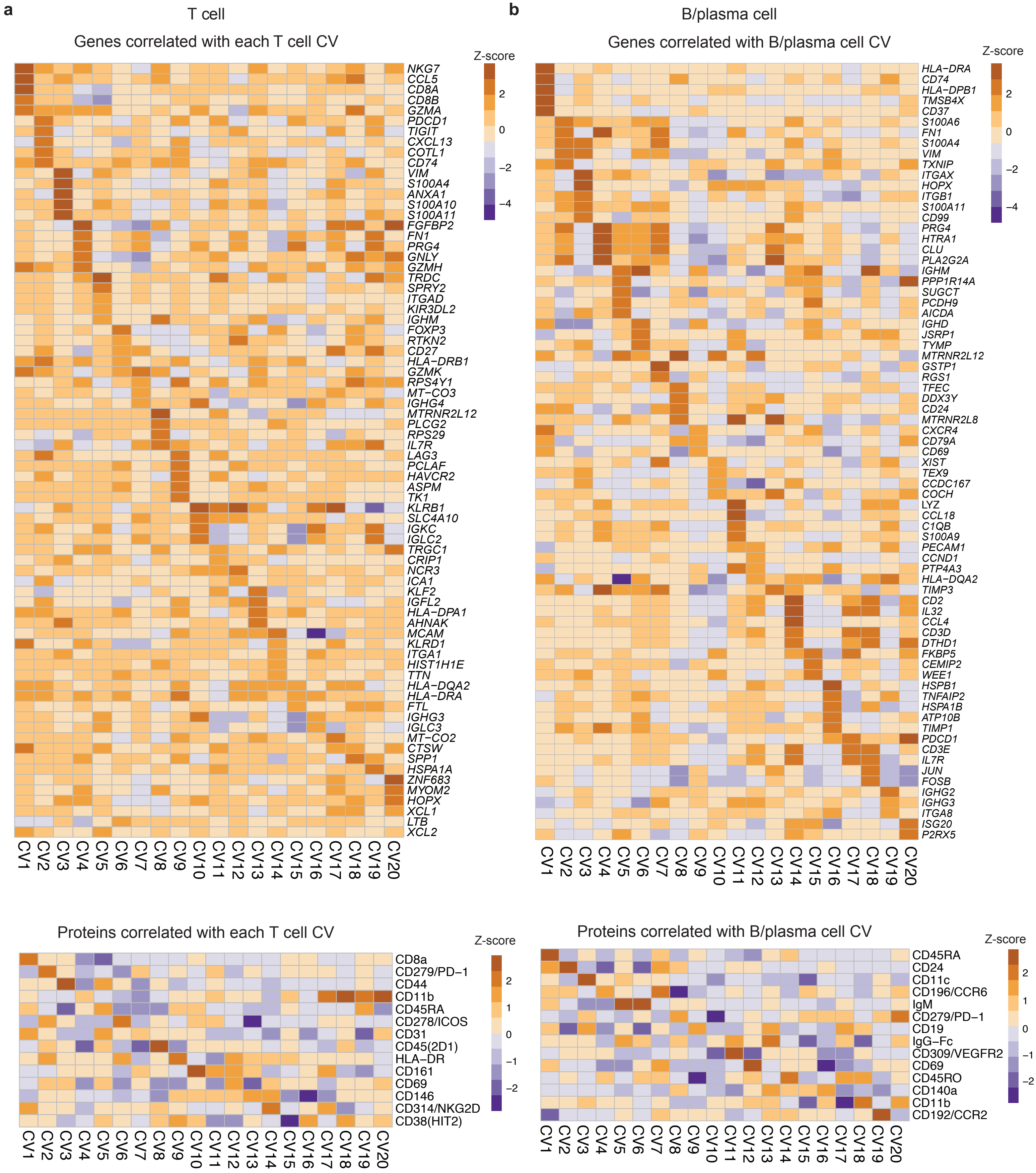

Supplementary Fig. 6. Correlations between gene and protein features and top 20 integrative CVs. Correlation z-scores for genes (top) and proteins (bottom) in **a,** T cells, and **b,** B/plasma cells.

Supplementary Figure 7

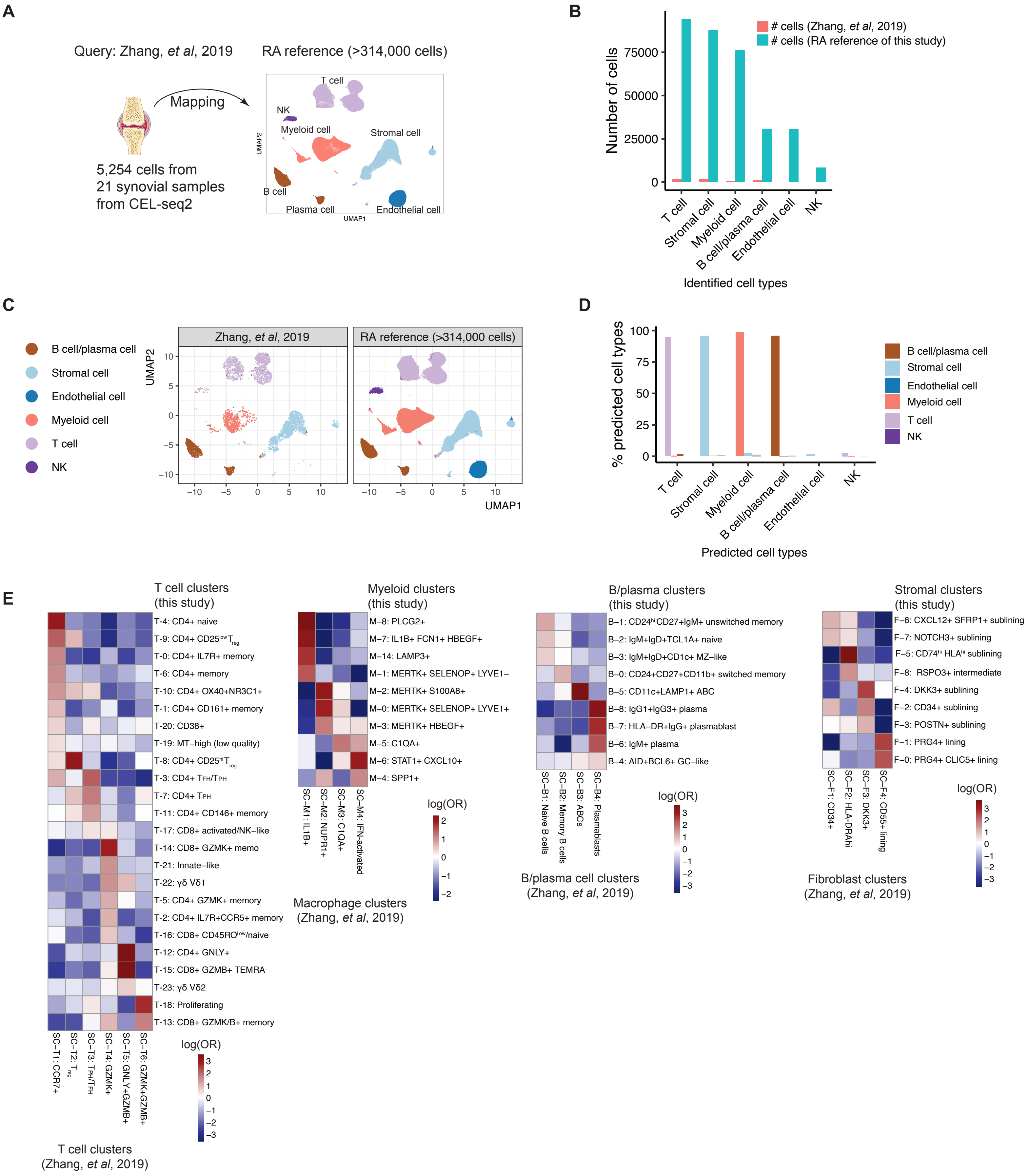

Supplementary Fig. 7. CITE-seq RA reference atlas to query other cells. a, We used Symphony[66] to map synovial cells from the AMP phase I RA dataset (Zhang, *et al.*, 2019) [8] onto this AMP phase II single-cell RA reference (current study), **b,** Barplot of number of cells per cell type in Phase I and Phase II. **c,** Cells from Phase I (query) and Phase II (reference) plotted in the same UMAP coordinates and colored by cell type. **d,** Barplot of reference-based cell-type-assignment (maximum of 5-nearest neighbors) for cells from the Phase I query, stratified by originally annotated cell state, **e,** We further used Symphony to map AMP Phase I cells from each cell type including B/plasma cells (n=1,142), T cells (n=1,529), fibroblasts (n=1,844), and macrophages (n=750) onto the corresponding cell-type-specific references from Phase II (B/plasma cell, T cell, stromal cell, and myeloid cell). Heatmaps show results for each major cell type, with rows corresponding to cell states from the Phase II reference and columns corresponding to cell states from Phase I. Blue-red color scale indicates the log(OR) for mapping a cell from the Phase I cluster to the Phase II cluster vs. mapping other Phase I cells to this Phase II cluster. Higher values indicate larger overlap between the corresponding Phase I and Phase II clusters.

Supplementary Figure 8

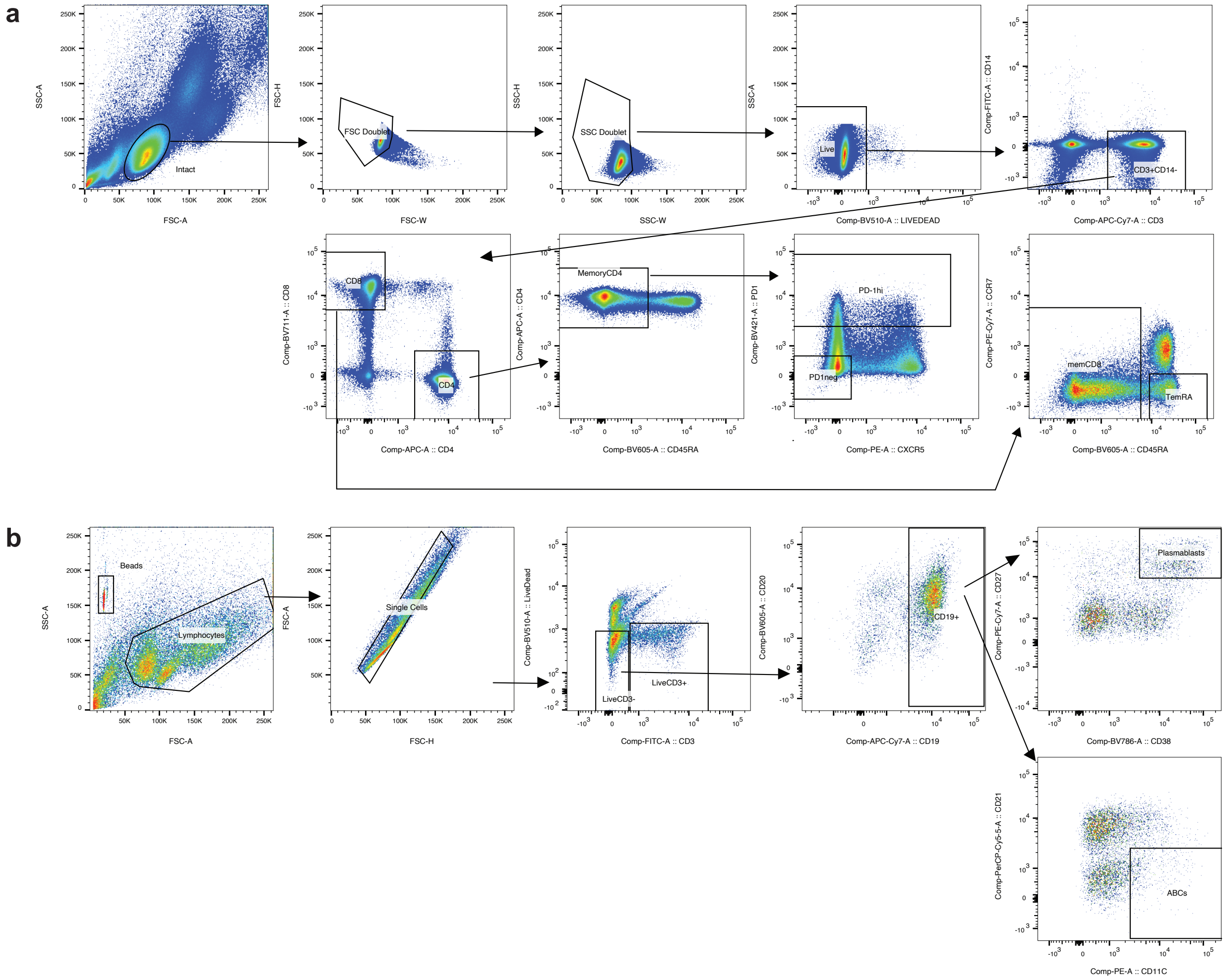

Supplementary Fig. 8. Representative flow cytometry gating for T-B cell co-culture assays. **a**, Representative gating of fluorescence-associated cell sorting of PD-1hi Tph and Tfh cells, PD-1low memory CD4 T cells, TemRA CD8 T cells and non-TemRA memory CD8 T cells. **B**, Representative gating of flow cytometry data to quantify B cell subsets after co-culture of B cells with T cells. Data are representative of three independent experiments, each with one T cell donor and one B cell donor.

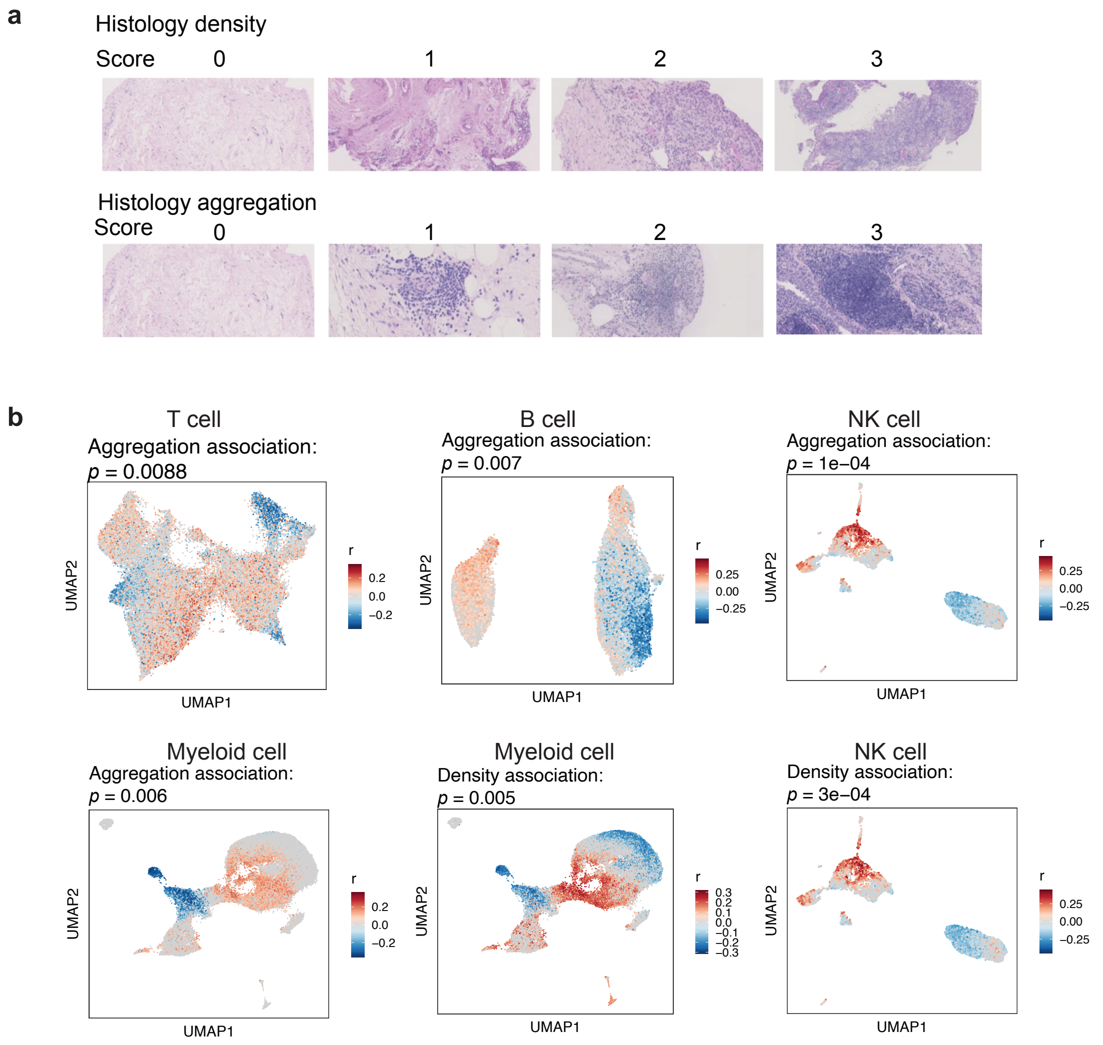
a
Histology density
Score 0
1
2
3
Histology aggregation
Score 0
1
2
3
b
T cell
Aggregation association:
p = 0.0088
B cell
Aggregation association:
p = 0.007
NK cell
Aggregation association:
p = 1e−04
Myeloid cell
Aggregation association:
p = 0.006
Myeloid cell
Density association:
p = 0.005
NK cell
Density association:
p = 3e−04
UMAP1
UMAP2
r

Supplementary Fig. 9. Clinical and histologic association results using CNA. a, Representative histologic images illustrating different levels of density and aggregation scores, **b,** For each broad cell type, the UMAP is colored by the results of CNA testing associations with histologic density and/or aggregation scores, controlling age, sex, and number of cells per sample. The red/blue color scale represents positive/negative associations whose correlations are FDR < 0.05;global p-values were obtained from permutation tests.

Supplementary Figure 10

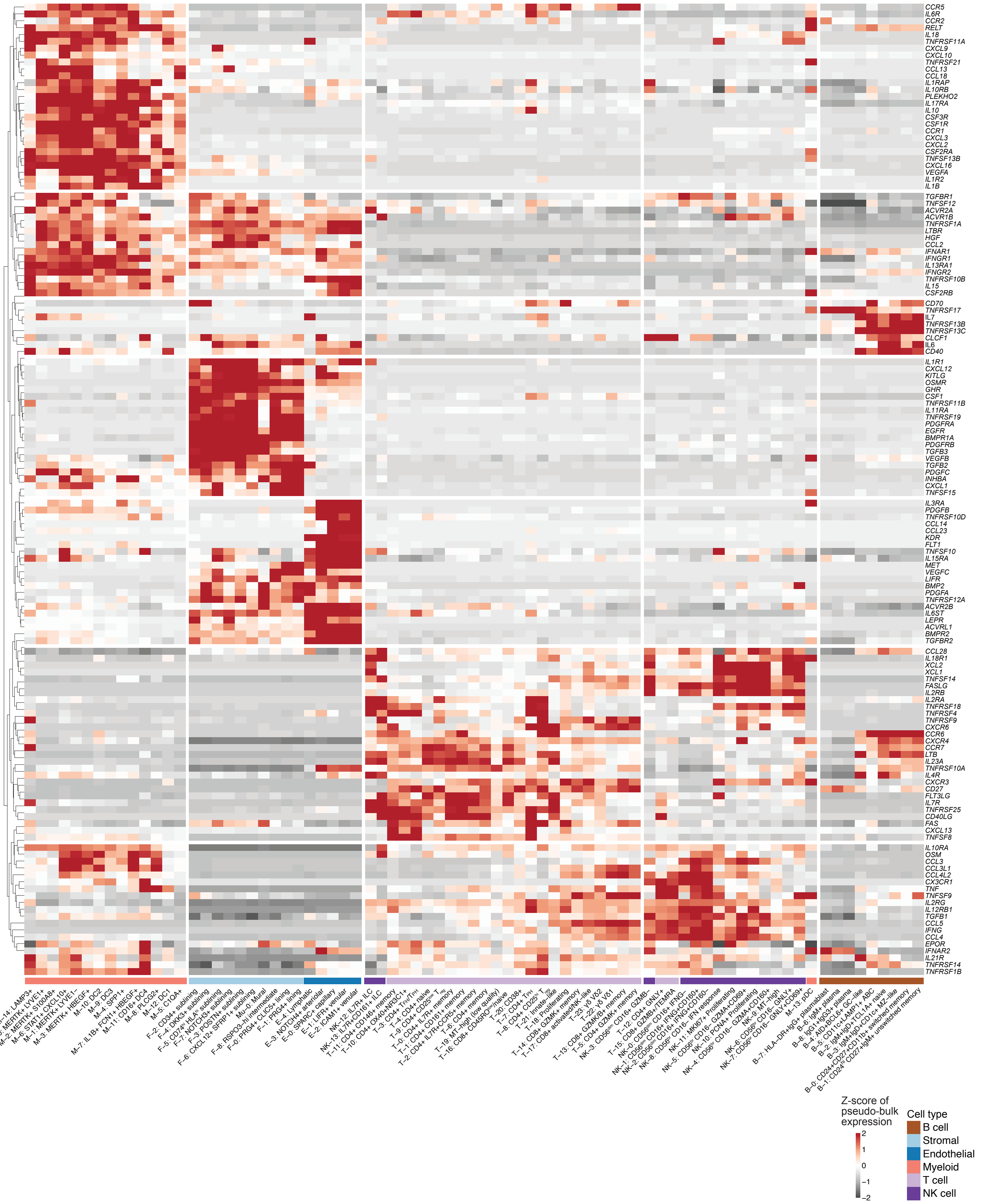

Supplementary Fig. 10. Cellular sources for cytokine/receptor RNA. Z-scored pseudo-bulk expression across the identified 77 cell states of a curated cytokine and receptor list from KEGG (M9809)[69] is shown. 138 cytokines and receptors that are expressed in more than 3% of total single cells are shown here.

Supplementary Figure 11

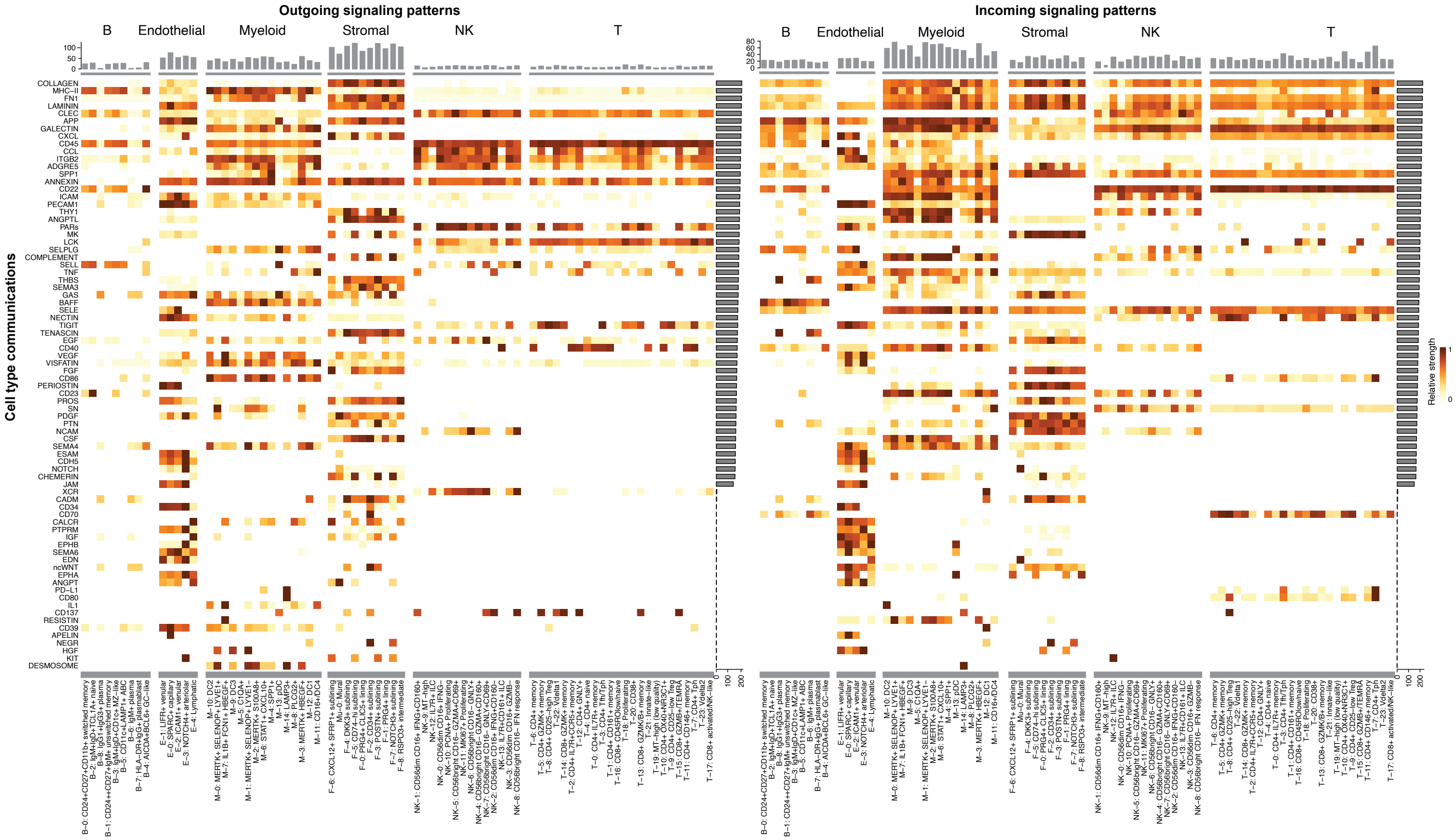

Supplementary Fig. 11. Inferred cell-cell interactions based on ligand-receptor analysis. Outgoing (left) and incoming (right) cell-state interaction patterns identified with CellChat. Boxes are colored based on the relative strength of the signaling molecule in that cluster.

Supplementary Figure 12

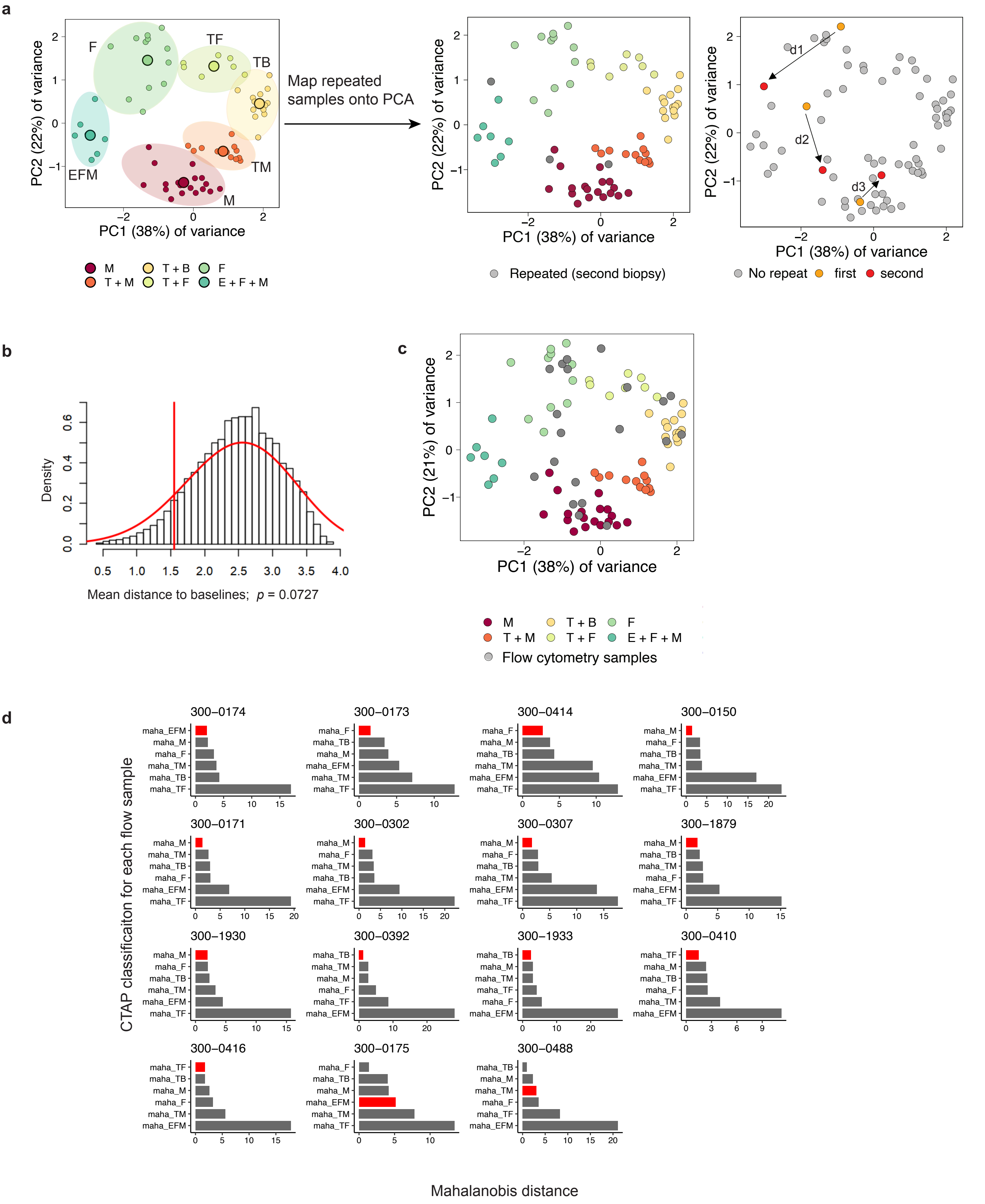

Supplementary Fig. 12. Classification of repeat biopsies and flow cytometry into CTAPs.
a, Mapping three repeated biopsy samples onto CTAP PC space based on the cell-type abundance, **b,** Histogram of Mahalanobis distance between 10,000 random samples to the baseline samples**, c,** Flow cytometry samples plotted in the same PCA coordinates as originally used to cluster CITE-seq samples into CTAPs**, d,** Mahalanobis distance between each flow sample and each CTAP centroid. The original CTAP of the single-cell samples from the same donors are labeled as red.

Supplementary Figure 13

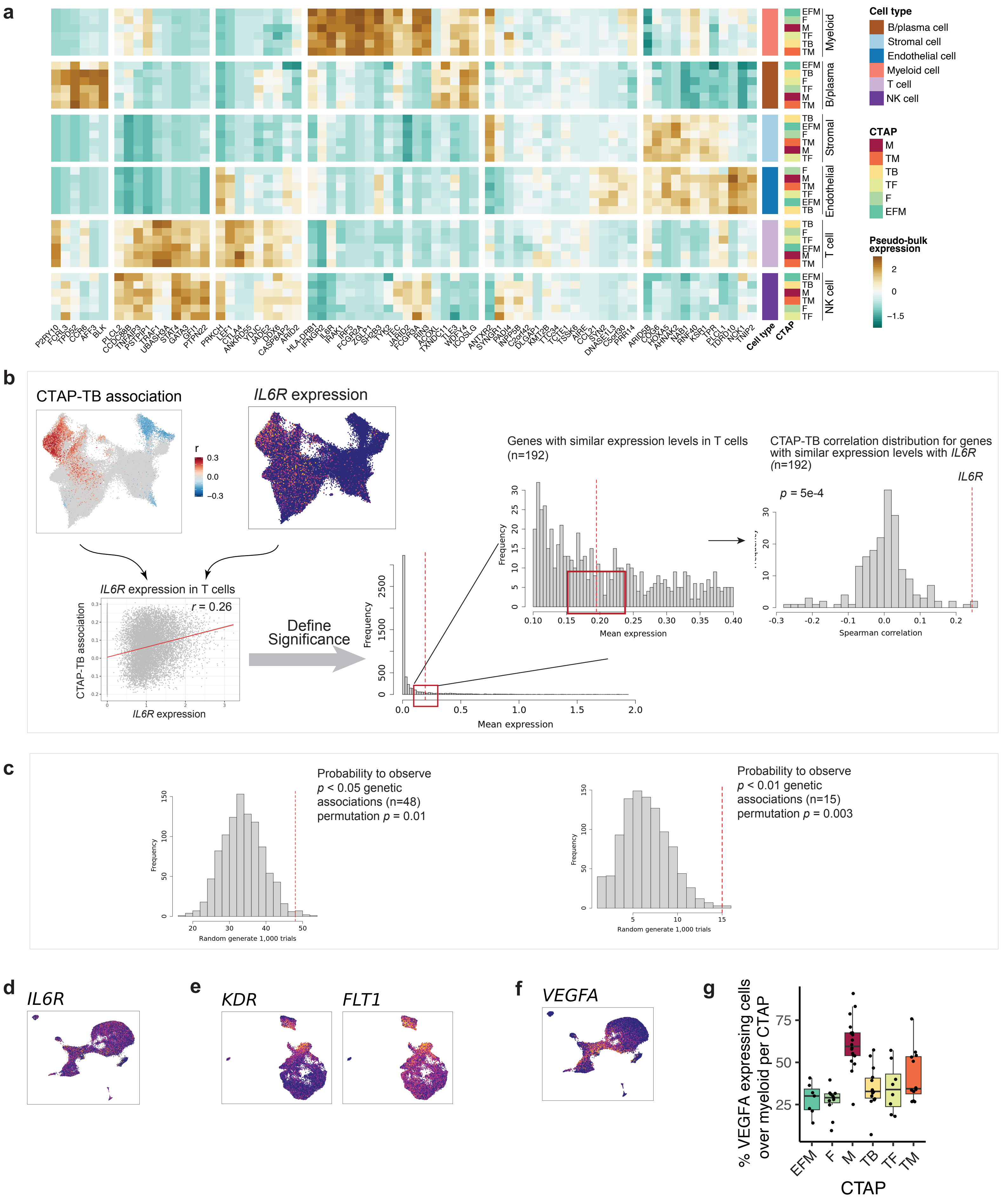

Supplementary Fig. 13. Expression of RA GWAS-implicated genes. a, Heatmap of pseudo-bulk normalized expression of genes implicated in RA GWAS studies in cells from each indicated cluster and CTAP combination. Color scale ranges from brown (high) to turquoise (low) and is scaled across cluster+CTAP combinations shown. **b,** Statistical strategy to correlate RA-associated genes with CTAP-associated cells. Briefly, we measured the Pearson correlation coefficient between an RA GWAS gene's normalized expression and the cell-neighborhood correlations with a CTAP (left). To define significance, we compared this correlation to the correlations computed between other genes of similar expression level and the same CTAP (right). **c,** Histogram of number of RA genes significantly correlated with CTAP-associated cells in at least one CTAP/cell type pair in null simulations. Significance threshold for correlation is $p < 0.05$ (left) or $p < 0.01$ (right). P-values are calculated with permutation tests (n=1,000). **d-f.** UMAPs colored by normalized expression of selected RA-associated genes in myeloid cells **(d,f)** or endothelial cells. **g**, Box plot of percent of myeloid cells expressing *VEGFA* in each CTAP. Points represent individuals (N=70); EFM (N=7), F (N=11), TF (N=8), TB (N=14), TM (n=12), M (N=18). Box plots show median (vertical bar), 25th and 75th percentiles (lower and upper bounds of the box, respectively) and 1.5 x IQR (or minimum/maximum values; end of whiskers).

Supplementary Table 1a. Demographic, clinical, and histology metrics for the study cohort (stratified by treatment group)

	DMARD naive	MTX inadequate	TNFi inadequate	P-value	Missing %
N	28	27	15		
Age in years, mean (SD)	56.43 (15.98)	52.11 (16.33)	63.20 (7.90)	0.074	0.0
Female, N (%)	18 (64.3)	22 (81.5)	12 (80.0)	0.293	0.0
Race and ethnicity, N (%)					0.0
White	16 (57.1)	19 (70.4)	14 (93.3)	0.047	
Black or African American	7 (25.0)	5 (18.5)	1 (6.7)	0.338	0.0
Hispanic	4 (14.3)	12 (44.4)	4 (26.7)	0.046	0.0
Years since diagnosis, mean (SD)	2.64 (8.33)	7.42 (8.80)	12.58 (8.79)	0.002	0.0
CRP (mg/dL), mean (SD)	2.09 (2.84)	2.40 (4.01)	1.08 (1.27)	0.468	10.0
ESR, mean (SD)	44.00 (29.66)	40.59 (30.96)	26.33 (20.58)	0.211	14.3
DAS28-CRP3, mean (SD)	4.84 (1.38)	5.08 (1.57)	4.55 (1.15)	0.561	10.0
CDAI, mean (SD)	32.25 (16.95)	39.69 (16.04)	29.69 (12.24)	0.093	0.0
Number of tender joints, mean (SD)	11.00 (8.52)	12.70 (9.29)	8.60 (6.88)	0.331	0.0
Number of swollen joints, mean (SD)	9.00 (8.16)	14.30 (6.69)	9.00 (5.44)	0.013	0.0
Patient global assessment, mean (SD)	6.33 (2.26)	5.25 (2.32)	6.03 (1.98)	0.227	8.6
Physician global assessment, mean (SD)	6.09 (1.67)	6.32 (1.59)	5.75 (1.70)	0.569	0.0
Serology					
Seropositive, N (%)	24 (85.7)	21 (77.8)	14 (100.0)	0.159	1.4
RF+, N (%)	21 (75.0)	18 (66.7)	11 (84.6)	0.471	2.9
anti-CCP+, N (%)	21 (75.0)	20 (74.1)	13 (92.9)	0.332	1.4
anti-CCP+ (u/mL), mean (SD)	165.22 (126.51)	158.68 (102.07)	269.47 (246.44)	0.059	0.0
Comorbidity, N (%)	13 (46.4)	7 (25.9)	6 (40.0)	0.281	0.0
Histology, mean (SD)					
Krenn lining	1.15 (0.61)	0.92 (0.56)	0.79 (0.43)	0.114	5.7
Krenn inflammation	2.07 (0.73)	1.62 (1.06)	1.50 (1.02)	0.100	4.3
Density	1.88 (0.65)	1.42 (0.55)	1.29 (0.65)	0.005	1.4
Aggregates	1.57 (1.29)	0.96 (1.19)	1.21 (1.12)	0.186	1.4
Pathotype	2.46 (0.64)	2.26 (0.66)	2.36 (0.74)	0.525	1.4

Note: Comorbidities include diabetes, cardiovascular disease, pulmonary disease, autoimmune thyroid disease, inflammatory
P-values are from one-way ANOVAs for continuous variables and chi-squared tests for binary variables.

Supplementary Table 1b: Demographic, clinical, and histology metrics for the study cohort (stratified by disease activity)

	Remission	Low disease activity	Moderate disease activity	High disease activity	P-value	Missing %
N	3	4	29	27		
Age in years, mean (SD)	49.00 (23.30)	68.00 (9.90)	56.28 (13.68)	56.22 (17.08)	0.414	0.0
Female, N (%)	3 (100.0)	3 (75.0)	21 (72.4)	19 (70.4)	0.749	0.0
Race and ethnicity, N (%)						
White	1 (33.3)	3 (75.0)	22 (75.9)	18 (66.7)	0.462	0.0
Black or African American	2 (66.7)	0 (0.0)	4 (13.8)	5 (18.5)	0.103	0.0
Hispanic	0 (0.0)	0 (0.0)	7 (24.1)	10 (37.0)	0.252	0.0
Years since diagnosis, mean (SD)	2.11 (1.93)	11.56 (19.62)	7.49 (8.89)	4.94 (8.52)	0.424	0.0
CRP (mg/dL), mean (SD)	0.00 (0.00)	0.55 (0.58)	1.07 (1.08)	3.43 (4.22)	0.012	10.0
ESR, mean (SD)	20.67 (5.77)	17.25 (10.75)	37.82 (28.54)	47.08 (31.25)	0.151	14.3
DAS28-CRP3, mean (SD)	2.36 (0.16)	2.89 (0.27)	4.17 (0.57)	6.18 (0.81)	<0.001	10.0
CDAI, mean (SD)	14.40 (3.50)	19.12 (3.97)	25.12 (8.90)	48.89 (12.95)	<0.001	0.0
Number of tender joints, mean (SD)	1.33 (1.15)	1.00 (1.41)	6.28 (4.49)	18.70 (6.68)	<0.001	0.0
Number of swollen joints, mean (SD)	5.00 (5.20)	7.25 (6.70)	6.90 (5.31)	16.59 (6.86)	<0.001	0.0
Patient global assessment, mean (SD)	2.50 (2.29)	5.38 (1.80)	5.70 (2.26)	6.36 (1.91)	0.032	8.6
Physician global assessment, mean (SD)	4.07 (1.10)	5.50 (2.08)	5.80 (1.70)	6.72 (1.41)	0.019	0.0
Serology						
Seropositive, N (%)	3 (100.0)	3 (75.0)	24 (82.8)	23 (85.2)	0.831	1.4
RF+, N (%)	3 (100.0)	1 (25.0)	20 (69.0)	20 (76.9)	0.120	2.9
anti-CCP+, N (%)	3 (100.0)	3 (75.0)	21 (72.4)	21 (77.8)	0.752	1.4
anti-CCP+ (u/mL), mean (SD)	247.67 (80.83)	137.62 (130.32)	181.00 (211.69)	174.89 (109.79)	0.854	0.0
Comorbidity, N (%)	1 (33.3)	3 (75.0)	8 (27.6)	13 (48.1)	0.194	0.0
Histology, mean (SD)						
Krenn lining	1.00 (0.00)	1.50 (0.58)	0.93 (0.65)	0.96 (0.46)	0.312	5.7
Krenn inflammation	2.00 (1.00)	2.00 (0.82)	1.79 (1.03)	1.81 (0.98)	0.966	4.3
Density	1.56 (0.51)	1.75 (0.50)	1.56 (0.72)	1.61 (0.70)	0.964	1.4
Aggregates	2.00 (1.73)	1.00 (1.15)	1.25 (1.29)	1.33 (1.21)	0.754	1.4
Pathotype	2.67 (0.58)	2.50 (0.58)	2.32 (0.67)	2.33 (0.73)	0.831	1.4

Note: Comorbidities include diabetes, cardiovascular disease, pulmonary disease, autoimmune thyroid disease, inflammatory bowel
P-values are from one-way ANOVAs for continuous variables and chi-squared tests for binary variables.

Supplementary Table 2: Antibodies used for CITE-seq panel

Category	Barcode	Specificity	Clone	Barcode Sequence	Amount of antibody per 100 uL staining volume (dilution)	Vendor	Catalog number
TotalSeq™-A	0155	CD107a (LAMP-1)	H4A3	CAGCCCACTGCAATA	0.2ug (1:250)	BioLegend	328647
TotalSeq™-A	0165	CD314 (NKG2D)	1D11	CGTGTTTGTTCCTCA	0.2ug (1:250)	BioLegend	320835
TotalSeq™-A	0050	CD19	HIB19	CTGGGCAATTACTCG	0.2ug (1:250)	BioLegend	302259
TotalSeq™-A	0080	CD8a	RPA-T8	GCTGCGCTTTCCATT	0.2ug (1:250)	BioLegend	301067
TotalSeq™-A	0181	CD21	Bu32	AACCTAGTAGTTCGG	0.2ug (1:250)	BioLegend	354915
TotalSeq™-A	0375	IgG Fc	M1310G05	CTGGAGCGATTAGAA	0.2ug (1:250)	BioLegend	410725
TotalSeq™-A	0597	CD209 (DC-SIGN)	9E9A8	TCACTGGACACTTAA	0.2ug (1:250)	BioLegend	330119
TotalSeq™-A	0132	EGFR	AY13	GCTTAACATTGGCAC	0.2ug (1:250)	BioLegend	352923
TotalSeq™-A	0143	CD196 (CCR6)	G034E3	GATCCCTTTGTCACT	0.2ug (1:250)	BioLegend	353437
TotalSeq™-A	0160	CD1c	L161	GAGCTACTTCACTCG	0.2ug (1:250)	BioLegend	331539
TotalSeq™-A	0362	CD309 (VEGFR2)	7D4-6	TTCACGCAGTAAGAT	0.2ug (1:250)	BioLegend	359919
TotalSeq™-A	0390	CD127 (IL-7Rα)	A019D5	GTGTGTTGTCCTATG	0.2ug (1:250)	BioLegend	351352
TotalSeq™-A	0008	CD273 (B7-DC, PD-L2)	24F.10C12	TCAACGCTTGGCTAG	0.2ug (1:250)	BioLegend	329619
TotalSeq™-A	0805	CD226 (DNAM-1)	TX25	AGACCAACTCATTCA	0.2ug (1:250)	BioLegend	337111
TotalSeq™-A	0171	CD278 (ICOS)	C398.4A	CGCGCACCCATTAAA	0.2ug (1:250)	BioLegend	313555
TotalSeq™-A	0219	CD119 (IFN-γ R α chain)	GIR-208	TGTGTATTCCCTTGT	0.2ug (1:250)	BioLegend	308607
TotalSeq™-A	0007	CD274 (B7-H1, PD-L1)	29E.2A3	GTTGTCCGACAATAC	0.2ug (1:250)	BioLegend	329743
TotalSeq™-A	0034	CD3	UCHT1	CTCATTGTAACTCCT	0.2ug (1:250)	BioLegend	300475
TotalSeq™-A	0383	CD55	JS11	GCTCATTACCCATTA	0.2ug (1:250)	BioLegend	311317
TotalSeq™-A	0136	IgM	MHM-88	TAGCGAGCCCGTATA	0.2ug (1:250)	BioLegend	314541
TotalSeq™-A	0023	CD155 (PVR)	SKII.4	ATCACATCGTTGCCA	1ug (1:50)	BioLegend	337623
TotalSeq™-A	0024	CD112 (Nectin-2)	TX31	AACCTTCCGTCTAAG	1ug (1:50)	BioLegend	337417
TotalSeq™-A	0045	CD4	SK3	GAGGTTAGTGATGGA	1ug (1:50)	BioLegend	344649
TotalSeq™-A	0053	CD11c	S-HCL-3	TACGCCTATAACTTG	1ug (1:50)	BioLegend	371519
TotalSeq™-A	0054	CD34	581	GCAGAAATCTCCCTT	1ug (1:50)	BioLegend	343537
TotalSeq™-A	0060	CD90 (Thy1)	5E10	GCATTGTACGATTCA	1ug (1:50)	BioLegend	328135
TotalSeq™-A	0063	CD45RA	HI100	TCAATCCTTCCGCTT	1ug (1:50)	BioLegend	304157
TotalSeq™-A	0083	CD16	3G8	AAGTTCACTCTTTGC	1ug (1:50)	BioLegend	302061
TotalSeq™-A	0087	CD45RO	UCHL1	CTCCGAATCATGTTG	1ug (1:50)	BioLegend	304255
TotalSeq™-A	0100	CD20	2H7	TTCTGGGTCCCTAGA	1ug (1:50)	BioLegend	302359
TotalSeq™-A	0127	Podoplanin	NC-08	GGTTACTCGTTGTGT	1ug (1:50)	BioLegend	337019
TotalSeq™-A	0128	CD140a (PDGFRα)	16A1	ATGCGCCGAGAATTA	1ug (1:50)	BioLegend	323509
TotalSeq™-A	0134	CD146	P1H12	CCTTGGATAACATCA	1ug (1:50)	BioLegend	361017
TotalSeq™-A	0141	CD195 (CCR5)	J418F1	CCAAAGTAAGAGCCA	1ug (1:50)	BioLegend	359135
TotalSeq™-A	0146	CD69	FN50	GTCTCTTGGCTTAAA	1ug (1:50)	BioLegend	310947
TotalSeq™-A	0149	CD161	HP-3G10	GTACGCAGTCCTTCT	1ug (1:50)	BioLegend	339945
TotalSeq™-A	0159	HLA-DR	L243	AATAGCGAGCAAGTA	1ug (1:50)	BioLegend	307659
TotalSeq™-A	0162	CD64	10.1	AAGTATGCCCTACGA	1ug (1:50)	BioLegend	305037
TotalSeq™-A	0180	CD24	ML5	AGATTCCTTCGTGTT	1ug (1:50)	BioLegend	311137
TotalSeq™-A	0242	CD192 (CCR2)	K036C2	GAGTTCCCTTACCTG	1ug (1:50)	BioLegend	357229
TotalSeq™-A	0358	CD163	GHI/61	GCTTCTCCTTCCTTA	1ug (1:50)	BioLegend	333635
TotalSeq™-A	0073	CD44	IM7	TGGCTTCAGGTCCTA	1ug (1:50)	BioLegend	103045
TotalSeq™-A	0163	CD141 (Thrombomodulin)	M80	GGATAACCGCGCTTT	1ug (1:50)	BioLegend	344121
TotalSeq™-A	0191	CD27	LG.3A10	CAAGGTATGTCACTG	1ug (1:50)	BioLegend	124235
TotalSeq™-A	0205	CD206 (MMR)	15-2	TCAGAACGTCTAACT	1ug (1:50)	BioLegend	321143
TotalSeq™-A	0427	Folate Receptor β (FR-β)	94b/FOLR2	TGTGGCTAGTCAGTT	1ug (1:50)	BioLegend	391707
TotalSeq™-A	0048	CD45	2D1	TCCCTTGCGATTTAC	1ug (1:50)	BioLegend	368543
TotalSeq™-A	0124	CD31	WM59	ACCTTTATGCCACGG	1ug (1:50)	BioLegend	303137
TotalSeq™-A	0161	CD11b	ICRF44	GACAAGTGATCTGCA	1ug (1:50)	BioLegend	301353
TotalSeq™-A	0234	CD68	Y1/82A	CGGTGTTTGTAGCAA	1ug (1:50)	BioLegend	custom conjugate
TotalSeq™-A	0389	CD38	HIT2	TGTACCCGCTTGTGA	1ug (1:50)	BioLegend	303541
TotalSeq™-A	0400	CD144 (VE-Cadherin)	BV9	TCCACTCATTCTGTA	1ug (1:50)	BioLegend	348517
TotalSeq™-A	0406	CD304 (Neuropilin-1)	12C2	GGACTAAGTTTCGTT	1ug (1:50)	BioLegend	354525
TotalSeq™-A	0006	CD86	IT2.2	GTCTTTGTCAGTGCA	1ug (1:50)	BioLegend	305443
TotalSeq™-A	0088	CD279 (PD-1)	EH12.2H7	ACAGCGCCGTATTTA	1ug (1:50)	BioLegend	329955
TotalSeq™-A	0179	CX3CR1	K0124E1	AGTATCGTCTCTGGG	1ug (1:50)	BioLegend	355709
TotalSeq™-A	0047	CD56 (NCAM)	QA17A16	TCCTTTCCTGATAGG	1ug (1:50)	BioLegend	custom barcode
TotalSeq™-A	0081	CD14	63D3	TCTCAGACCTCCGTA	1ug (1:50)	BioLegend	custom barcode

Supplementary Table 3: Parameters for single-cell integration and clustering

Cell type	Number of variable genes selected per sample	Total number of variable genes	KL divergence threshold for proteins	Number of proteins selected for CCA	Harmony theta	Louvain clustering resolution
Global	1000	5751	0.3	36	1	NULL
B and plasma	400	6647	0.025	23	0	0.6
T cell	400	6603	0.025	21	1	1.6

Cell type	Number of variable genes selected per sample	Total number of variable genes	KL divergence threshold for proteins	Number of proteins selected for CCA	Harmony theta	Louvain clustering resolution
Endothelial	400	5993	NULL	NULL	1	0.3
Stromal	400	3758	NULL	NULL	1	0.6
Myeloid	400	3547	NULL	NULL	1	0.6
NK	300	4987	NULL	NULL	1	0.8

Supplementary Table 4: Percentages of cell types by CTAP

CTAP	Endothelial	Stromal	Myeloid	T cell	B cell	NK cell
EFM	29%	28%	29%	11%	1%	2%
F	10%	51%	17%	14%	6%	2%
M	8%	11%	44%	26%	9%	3%
TB	4%	6%	11%	50%	23%	5%
TF	5%	30%	7%	44%	10%	3%
TM	8%	6%	27%	48%	8%	3%
Overall	11%	22%	25%	30%	10%	3%

Supplementary Table 5: Differentially expressed genes per CITE-seq cluster

This table is provided separately in .xlsx format

Supplementary Table 6: Associations of previously identified RA cell states

Zhang, et al, 2019 (phase 1)		This study (phase 2)					
Pre-identified RA expanded clusters defined by Leukocyte-rich RA vs Leukocyte-poor RA and OA	# cells	Matched clusters	# cells	RA vs OA Odds Ratio (CI)	RA vs OA MASC p-value	Leukocyte-rich RA vs Leukocyte-poor RA and OA Odds Ratio (CI)	Leukocyte-rich RA vs Leukocyte-poor RA and OA MASC p-value
HLA+ fibroblast (SC-F2)	719	F-5, F-8	6860	2.7 (1.1-6.9)	0.04	3.3 (1.9-5.8)	5E-05
Tph/Tfh (SC-T3)	70	T-3, T-7	8966	1.9 (1.0-3.6)	0.04	1.63 (1.1-2.4)	0.01
ABCs (SC-B3)	48	B-5	1965	2.5 (1.3-4.9)	8E-03	1.9 (1.2-2.9)	5E-03
IL1B+ (SC-M1)	349	M-1, M-7, M-8, M-14	21219	1.6 (0.9-2.9)	0.06	3.1 (1.9-5.1)	1E-04
IFN-activated (SC-M4)	71	M-6	1715	2.9 (1.6-5.3)	2E-03	2.4 (1.6-3.5)	3E-05

95% confidence interval (CI) for the odds ratio (OR) and one-sided MASC (mixed-effects modeling of associations) p-value are shown.

Supplementary Table 7: Statistics of single-cell associations with CTAPs and histologic parameters

This table is provided separately in .xlsx format

Supplementary Table 8. Percentages of cell types within each CTAP

This table is provided separately in .xlsx format

Supplementary Table 9: Demographic, clinical, and histologic metrics across RA CTAPs

	CTAP-EFM	CTAP-F	CTAP-M	CTAP-TB	CTAP-TF	CTAP-TM	P-value	Missing %
N	7	11	18	14	8	12		
Age in years, mean (SD)	66.57 (7.11)	56.18 (14.43)	56.17 (20.79)	54.50 (12.87)	58.00 (9.07)	51.08 (14.44)	0.435	0.0
Female, N (%)	6 (85.7)	7 (63.6)	13 (72.2)	11 (78.6)	6 (75.0)	9 (75.0)	0.934	0.0
Race and ethnicity, N (%)								
White	5 (71.4)	7 (63.6)	12 (66.7)	11 (78.6)	5 (62.5)	9 (75.0)	0.949	0.0
Black or African American	1 (14.3)	4 (36.4)	2 (11.1)	2 (14.3)	1 (12.5)	3 (25.0)	0.587	0.0
Hispanic	1 (14.3)	3 (27.3)	4 (22.2)	5 (35.7)	4 (50.0)	3 (25.0)	0.655	0.0
Years since diagnosis, mean (SD)	12.08 (8.79)	4.39 (7.36)	8.36 (13.33)	7.42 (7.87)	5.85 (8.32)	2.41 (3.01)	0.281	0.0
CRP (mg/dL), mean (SD)	0.61 (0.41)	0.85 (0.72)	2.23 (3.98)	2.28 (2.72)	5.02 (6.34)	1.99 (2.14)	0.251	10.0
ESR, mean (SD)	24.17 (22.52)	40.22 (28.03)	41.44 (31.77)	37.45 (21.13)	48.80 (45.48)	40.36 (29.54)	0.813	14.3
DAS28-CRP3, mean (SD)	4.47 (0.71)	4.80 (1.46)	4.61 (1.34)	4.81 (1.29)	5.34 (2.56)	5.43 (1.49)	0.626	10.0
CDAI, mean (SD)	28.86 (10.45)	34.87 (16.55)	31.74 (13.96)	30.28 (15.55)	40.98 (21.88)	42.62 (16.01)	0.241	0.0
Number of tender joints, mean (SD)	9.00 (4.04)	12.45 (9.36)	8.67 (6.93)	9.86 (7.34)	13.12 (12.18)	15.08 (10.00)	0.363	0.0
Number of swollen joints, mean (SD)	7.71 (6.02)	12.36 (7.86)	10.06 (7.28)	9.57 (7.23)	12.88 (9.01)	13.75 (7.35)	0.469	0.0
Patient global assessment, mean (SD)	4.83 (1.40)	4.82 (1.65)	6.03 (2.58)	5.12 (2.78)	7.75 (1.56)	6.55 (1.12)	0.031	8.6
Physician global assessment, mean (SD)	6.21 (1.91)	5.37 (1.78)	6.35 (1.57)	5.64 (1.51)	6.91 (1.50)	6.37 (1.59)	0.308	0.0
Serology								
Seropositive, N (%)	6 (85.7)	9 (81.8)	13 (72.2)	13 (92.9)	7 (100.0)	11 (91.7)	0.435	1.4
RF+, N (%)	5 (71.4)	7 (63.6)	10 (55.6)	11 (84.6)	7 (100.0)	10 (83.3)	0.187	2.9
anti-CCP+, N (%)	6 (85.7)	9 (81.8)	9 (50.0)	13 (92.9)	7 (100.0)	10 (83.3)	0.027	1.4
anti-CCP+ (u/mL), mean (SD)	203.51 (94.45)	186.95 (100.33)	86.91 (101.88)	243.46 (266.58)	262.12 (52.79)	200.15 (113.68)	0.041	0.0
Comorbidity, N (%)	3 (42.9)	3 (27.3)	7 (38.9)	3 (21.4)	3 (37.5)	7 (58.3)	0.498	0.0
Histology, mean (SD)								
Krenn lining	0.57 (0.53)	1.00 (0.45)	1.29 (0.59)	0.62 (0.51)	1.33 (0.82)	1.00 (0.00)	0.003	5.7
Krenn inflammation	1.14 (1.07)	1.10 (0.74)	2.00 (0.94)	2.07 (1.00)	1.75 (0.71)	2.09 (0.83)	0.033	4.3
Density	0.83 (0.45)	1.19 (0.48)	1.81 (0.60)	1.74 (0.68)	1.65 (0.62)	1.71 (0.63)	0.005	1.4
Aggregates	0.67 (0.82)	0.55 (1.04)	1.17 (1.25)	1.57 (1.22)	1.62 (1.06)	1.75 (1.42)	0.117	1.4
Pathotype	2.17 (0.75)	2.00 (0.63)	2.39 (0.50)	2.43 (0.76)	2.75 (0.46)	2.42 (0.79)	0.238	1.4
Medication, N (%)								
Corticosteroid	1 (14.3)	3 (27.3)	4 (22.2)	4 (28.6)	4 (50.0)	4 (33.3)	0.700	0.0
Any DMARD	6 (85.7)	7 (63.6)	7 (38.9)	9 (64.3)	4 (50.0)	8 (66.7)	0.319	0.0
Methotrexate	1 (14.3)	4 (36.4)	6 (33.3)	7 (50.0)	3 (37.5)	5 (41.7)	0.734	0.0
TNFi	3 (42.9)	1 (9.1)	1 (5.6)	2 (14.3)	0 (0.0)	2 (16.7)	0.157	0.0
Treatment group, N (%)							0.152	0.0
DMARD naive	1 (14.3)	5 (45.5)	9 (50.0)	5 (35.7)	4 (50.0)	4 (33.3)		
Methotrexate naive	1 (14.3)	5 (45.5)	7 (38.9)	7 (50.0)	2 (25.0)	5 (41.7)		
TNFi inadequate	5 (71.4)	1 (9.1)	2 (11.1)	2 (14.3)	2 (25.0)	3 (25.0)		

Note: Comorbidities include diabetes, cardiovascular disease, pulmonary disease, autoimmune thyroid disease, inflammatory bowel disease, psoriasis, multiple sclerosis,
P-values are from one-way ANOVAs for continuous variables and chi-squared tests for binary variables.

Supplementary Table 10: Antibodies used in synovial tissue flow cytometry panel

Marker	Clone	Fluorochrome	Vendor	Catalog number	Dilution
CD146	P1H12	BV421	BioLegend	361004	1:200
CD8	SK1	BV510	BioLegend	344732	1:100
CD3	UCHT1	BV605	BioLegend	300460	1:50
PD-1	EH12.2H7	BV650	BioLegend	329950	1:50
HLA-DR	L243	BV711	BioLegend	307644	1:50
CD19	HIB19	BV785	BioLegend	302240	1:50
CD45	HI30	FITC	BioLegend	304006	1:200
CD11c	3.9	PerCP Cy5.5	BioLegend	301624	1:50
CD27	M-T271	PE	BioLegend	356406	1:100
CD14	M5E2	PE Dazzle 594	BioLegend	301852	1:200
CD90	5E10	PE Cy7	BioLegend	328124	1:200
CD4	OKT4	APC	BioLegend	317416	1:50
CD31	WM59	Alexa 700	BioLegend	303134	1:200
CD235a	11E4B-7-6 (KC16)	APC-Alexa 750	Beckman Coulter	A89314	1:100

Supplementary References

50. Weisenfeld, D. *et al.* Associations between rheumatoid arthritis clinical factors with synovial cell types and states. *bioRxiv* (2023) doi:10.1101/2023.04.27.23289104.
51. Prevoo, M. L. *et al.* Modified disease activity scores that include twenty-eight-joint counts. Development and validation in a prospective longitudinal study of patients with rheumatoid arthritis. *Arthritis Rheum.* **38**, 44–48 (1995).
52. Wells, G. *et al.* Validation of the 28-joint Disease Activity Score (DAS28) and European League Against Rheumatism response criteria based on C-reactive protein against disease progression in patients with rheumatoid arthritis, and comparison with the DAS28 based on erythrocyte sedimentation rate. *Ann. Rheum. Dis.* **68**, 954–960 (2009).
53. Choi, I. Y. *et al.* Stromal cell markers are differentially expressed in the synovial tissue of patients with early arthritis. *PLoS One* **12**, e0182751 (2017).
54. Krenn, V. *et al.* Synovitis score: discrimination between chronic low-grade and high-grade synovitis. *Histopathology* **49**, 358–364 (2006).
55. Donlin, L. T. *et al.* Methods for high-dimensonal analysis of cells dissociated from cyropreserved synovial tissue. *Arthritis Res. Ther.* **20**, 139 (2018).
56. Stoeckius, M. *et al.* Simultaneous epitope and transcriptome measurement in single cells. *Nat. Methods* **14**, 865–868 (2017).
57. Wolock, S. L., Lopez, R. & Klein, A. M. Scrublet: Computational Identification of Cell Doublets in Single-Cell Transcriptomic Data. *Cell Syst* **8**, 281–291.e9 (2019).
58. Stuart, T. *et al.* Comprehensive Integration of Single-Cell Data. *Cell* **177**, 1888–1902.e21 (2019).
59. Baglama, J. & Reichel, L. Augmented implicitly restarted lanczos bidiagonalization methods. *SIAM J. Sci. Comput.* **27**, 19–42 (2005).
60. Korsunsky, I. *et al.* Fast, sensitive and accurate integration of single-cell data with Harmony. *Nat. Methods* (2019) doi:10.1038/s41592-019-0619-0.
61. McInnes, L., Healy, J. & Melville, J. UMAP: Uniform Manifold Approximation and Projection for Dimension Reduction. *arXiv [stat.ML]* (2018).
62. Nathan, A. *et al.* Multimodally profiling memory T cells from a tuberculosis cohort identifies cell state associations with demographics, environment and disease. *Nat. Immunol.* **22**, 781–793 (2021).
63. González, I., Déjean, S., Martin, P. & Baccini, A. CCA: An R Package to Extend Canonical Correlation Analysis. *Journal of Statistical Software, Articles* **23**, 1–14 (2008).
64. Blondel, V. D., Guillaume, J.-L., Lambiotte, R. & Lefebvre, E. Fast unfolding of communities in large networks. *arXiv [physics.soc-ph]* (2008).
65. Slowikowski, K. *cellguide: Navigate single-cell RNA-seq datasets in your web browser*. (2023). doi:10.5281/zenodo.8144195.
66. Kang, J. B. *et al.* Efficient and precise single-cell reference atlas mapping with Symphony. *Nat. Commun.* **12**, 1–21 (2021).
67. Reshef, Y. A. *et al.* Co-varying neighborhood analysis identifies cell populations associated

with phenotypes of interest from single-cell transcriptomics. *Nat. Biotechnol.* 1–9 (2021).
68. Bates, D., Mächler, M., Bolker, B. & Walker, S. Fitting Linear Mixed-Effects Models Using lme4. *Journal of Statistical Software, Articles* **67**, 1–48 (2015).
69. Kanehisa, M., Sato, Y., Kawashima, M., Furumichi, M. & Tanabe, M. KEGG as a reference resource for gene and protein annotation. *Nucleic Acids Res.* **44**, D457–62 (2016).
70. Boardman, D. A. *et al.* Flagellin-specific human CAR Tregs for immune regulation in IBD. *J. Autoimmun.* **134**, 102961 (2023).
71. Kang, J. B. *et al.* Mapping the dynamic genetic regulatory architecture of HLA genes at single-cell resolution. *medRxiv* (2023) doi:10.1101/2023.03.14.23287257.
72. Purcell, S. *et al.* PLINK: a tool set for whole-genome association and population-based linkage analyses. *Am. J. Hum. Genet.* **81**, 559–575 (2007).
73. Sakaue, S. *et al.* Tutorial: a statistical genetics guide to identifying HLA alleles driving complex disease. *Nat. Protoc.* 1–17 (2023).
74. Luo, Y. *et al.* A high-resolution HLA reference panel capturing global population diversity enables multi-ancestry fine-mapping in HIV host response. *Nat. Genet.* **53**, 1504–1516 (2021).
75. Jia, X. *et al.* Imputing amino acid polymorphisms in human leukocyte antigens. *PLoS One* **8**, e64683 (2013).
76. Han, B. *et al.* Fine mapping seronegative and seropositive rheumatoid arthritis to shared and distinct HLA alleles by adjusting for the effects of heterogeneity. *Am. J. Hum. Genet.* **94**, 522–532 (2014).
77. Jin, S. *et al.* Inference and analysis of cell-cell communication using CellChat. *Nat. Commun.* **12**, 1088 (2021).
