# Supplementary material for: Deconstruction of rheumatoid arthritis synovium defines inflammatory subtypes
Source: Nature. 2023 Nov 8;623(7987):616–24. doi: 10.1038/s41586-023-06708-y (PMC10651487; doi:10.1038/s41586-023-06708-y)
Supplement: Supplementary file 2 — Reporting Summary [file 41586_2023_6708_MOESM2_ESM.pdf]

Corresponding author(s): Soumya Raychaudhuri (soumya@broadinstitute.org)

Last updated by author(s): Sep 6, 2023

Reporting Summary

Nature Portfolio wishes to improve the reproducibility of the work that we publish. This form provides structure for consistency and transparency in reporting. For further information on Nature Portfolio policies, see our Editorial Policies and the Editorial Policy Checklist.

Statistics

For all statistical analyses, confirm that the following items are present in the figure legend, table legend, main text, or Methods section.

n/a	Confirmed	
☐	☒	The exact sample size (n) for each experimental group/condition, given as a discrete number and unit of measurement
☐	☒	A statement on whether measurements were taken from distinct samples or whether the same sample was measured repeatedly
☐	☒	The statistical test(s) used AND whether they are one- or two-sided<br>*Only common tests should be described solely by name; describe more complex techniques in the Methods section.*
☐	☒	A description of all covariates tested
☐	☒	A description of any assumptions or corrections, such as tests of normality and adjustment for multiple comparisons
☐	☒	A full description of the statistical parameters including central tendency (e.g. means) or other basic estimates (e.g. regression coefficient) AND variation (e.g. standard deviation) or associated estimates of uncertainty (e.g. confidence intervals)
☐	☒	For null hypothesis testing, the test statistic (e.g. F, t, r) with confidence intervals, effect sizes, degrees of freedom and P value noted<br>*Give P values as exact values whenever suitable.*
☒	☐	For Bayesian analysis, information on the choice of priors and Markov chain Monte Carlo settings
☒	☐	For hierarchical and complex designs, identification of the appropriate level for tests and full reporting of outcomes
☐	☒	Estimates of effect sizes (e.g. Cohen's d, Pearson's r), indicating how they were calculated

Our web collection on statistics for biologists contains articles on many of the points above.

Software and code

Policy information about availability of computer code

Data collection	Clinical data were collected using REDCap v. 6.9.0 onwards. Flow cytometry data of disaggregate synovial tissue were acquired on a BD FACSAria Fusion using FACSDiva software v. 8.0.1. For functional experiments, T cell and B cell subsets were isolated using a BD FACSAria Fusion sorter, and analytic flow cytometry was performed on a BD Fortessa analyzer (B cell differentiation) or a BD Canto II analyzer (cytotoxicity assays), all using FACSDiva software.
Data analysis	Flow cytometry data were analyzed with FlowJo v10.6. Immunofluorescence microscopy images were analyzed with the Visiopharm platform (version 2022.10). Single-cell RNA-seq data were aligned and quantified with Cell Ranger (v. 3.1.0). Bulk RNA-seq data were aligned and quantified with STAR (v. 2.5.3). Other analyses were conducted with R (version 3.6 and 4.0) and Python (version 3.10).<br>Scripts to reproduce analyses are available on GitHub (https://github.com/immunogenomics/RA_Atlas_CITEseq) and Zenodo (https://zenodo.org/record/8118599).

For manuscripts utilizing custom algorithms or software that are central to the research but not yet described in published literature, software must be made available to editors and reviewers. We strongly encourage code deposition in a community repository (e.g. GitHub). See the Nature Portfolio guidelines for submitting code & software for further information.

Data

Policy information about availability of data

All manuscripts must include a data availability statement. This statement should provide the following information, where applicable:

- Accession codes, unique identifiers, or web links for publicly available datasets
- A description of any restrictions on data availability
- For clinical datasets or third party data, please ensure that the statement adheres to our policy

CITE-seq single-cell expression matrices and sequencing, bulk expression matrices, genotyping, and clinical data are available on Synapse (doi:10.7303/syn52297840). A cell browser website https://immunogenomics.io/ampra2/ is available to visualize our data and results. AMP Phase 1 single-cell data from Zhang*, Wei*, Slowikowski*, Rao*, Fonseka*, et al. 2019 are available on Immport (accession: SDY998). PEAC clinical trial RNA-seq data from Lewis, et al. 2019 are available on ArrayExpress (accession: E-MTAB-6141). R4RA clinical trial RNA-seq data from Rivellese*, Surace*, et al. 2022 are available on ArrayExpress (accession: E-MTAB-11611). Single-cell and bulk RNA sequencing data were aligned to GRCh38 (Ensembl 93), available as part of Cell Ranger v. 3.1.0.

Field-specific reporting

Please select the one below that is the best fit for your research. If you are not sure, read the appropriate sections before making your selection.

☒ Life sciences ☐ Behavioural & social sciences ☐ Ecological, evolutionary & environmental sciences

For a reference copy of the document with all sections, see nature.com/documents/nr-reporting-summary-flat.pdf

Life sciences study design

All studies must disclose on these points even when the disclosure is negative.

Sample size	Sample size (n = 82) was based on sample recruitment. No formal power calculations were performed because this was a discovery cohort with multiple advanced technical output measures. Cohort size was determined by approximate estimates of power for identifying differences between the treatment-naive, methotrexate inadequate-responder, and TNF inhibitor inadequate-responder groups.
Data exclusions	Biopsies that lacked synovial tissue on histological exam were excluded from the pipeline. Biopsies that yielded <400 live cells by flow cytometric sorting were excluded. Quality control of the sequencing data excluded cells with fever than 500 genes or more than 20% of UMIs from mitochondrial genes. Doublets were removed using Scrublet and a linear-discriminant analysis-based classifier. Three samples with <40% of cells passing QC were excluded from the analysis.
Replication	CITE-seq was conducted once per sample, with a total of 82 samples. CITE-seq data were validated by comparing cell type proportions based on CITE-seq to those calculated in the same samples with flow cytometry (n = 18). All attempts at replication were successful.<br>For the T/B cell co-culture experiment, the experiment was conducted independently on three biological replicates.<br>For the myeloid differentiation experiment, samples were randomly assigned to experimental groups (i.e., stimulus conditions). There were three biological replicates for each stimulus condition.
Randomization	Samples were randomized into tissue disaggregation processing batches based on treatment group and collection site. Other experiments did not have experimental groups, and there were no treatment interventions provided by this study, so randomization was not otherwise relevant.
Blinding	No blinding was performed in this study due to the cross-sectional nature of the study. There were no treatment interventions provided by this study. Cell clustering and CTAP categorization were performed without regard to treatment history or other clinical parameters.

Reporting for specific materials, systems and methods

We require information from authors about some types of materials, experimental systems and methods used in many studies. Here, indicate whether each material, system or method listed is relevant to your study. If you are not sure if a list item applies to your research, read the appropriate section before selecting a response.

Materials & experimental systems

n/a	Involved in the study	
☐	☒	Antibodies
☐	☒	Eukaryotic cell lines
☒	☐	Palaeontology and archaeology
☒	☐	Animals and other organisms
☐	☒	Human research participants
☒	☐	Clinical data
☒	☐	Dual use research of concern

Methods

n/a	Involved in the study	
☒	☐	ChIP-seq
☐	☒	Flow cytometry
☒	☐	MRI-based neuroimaging

Antibodies

Antibodies used

An anti-CD235 antibody (1:100, clone 11E4B-7-6 (KC16), 1IM2211U, Beckman Coulter) was included in live cell sorting to exclude red blood cells.

Antibodies used for flow cytometry are listed with clone, dilution and catalog number (as in Supplementary Table 10): CD3 (UCHT1, 1:50, 300460), CD4 (OKT4, 1:50, 317416), CD8A (SK1, 1:100, 344732), CD11c (3.9, 1:50, 301624), CD14 (M5E2, 1:200, 301852), CD19 (HIB19, 1:50, 302240), CD27 (M-T271, 1:100, 356406), CD31 (WM59, 1:200, 303134), CD45 (HI30, 1:200, 304006), CD90 (5E10, 1:200, 328124), CD146 (P1H12, 1:200, 361004), HLA-DR (L243, 1:50, 307644), PD-1 (EH12.2H7, 1:50, 329950), all purchased from BioLegend.

CITE-seq was performed using the following TotalSeq-A antibodies from BioLegend, listed with clone and catalog numbers (barcode sequences, and dilutions in Supplementary Table 2): CD107a/LAMP-1 (H4A3, 328647); CD314/NKG2D (1D11, 320835); CD19 (HIB19, 302259); CD8a (RPA-T8, 301067); CD21 (Bu32, 354915); IgG Fc (M1310G05, 410725); CD209/DC-SIGN (9E9A8, 330119); EGFR (AY13, 352923); CD196/CCR6 (G034E3, 353437); CD1c (L161, 331539); CD309/VEGFR2 (7D4-6, 359919); CD127/IL-7Rα (A019D5, 351352); CD273/B7-DC/PD-L2 (24F.10C12, 329619); CD226/DNAM-1 (TX25, 337111); CD278/ICOS (C398.4A, 313555); CD119/IFN-γ R α chain (GIR-208, 308607); CD274/B7-H1/PD-L1 (29E.2A3, 329743); CD3 (UCHT1, 300475); CD55 (JS11, 311317); IgM (MHM-88, 314541); CD155/PVR (SKII.4, 337623); CD112/Nectin-2 (TX31, 337417); CD4 (SK3, 344649); CD11c (S-HCL-3, 371519); CD34 (581, 343537); CD90/Thy1 (5E10, 328135); CD45RA (HI100, 304157); CD16 (3G8, 302061); CD45RO (UCHL1, 304255); CD20 (2H7, 302359); Podoplanin (NC-08, 337019); CD140a/PDGFRα (16A1, 323509); CD146 (P1H12, 361017); CD195/CCR5 (J418F1, 359135); CD69 (FN50, 310947); CD161 (HP-3G10, 339945); HLA-DR (L243, 307659); CD64 (10.1, 305037); CD24 (ML5, 311137); CD192/CCR2 (K036C2, 357229); CD163 (GHI/61, 333635); CD44 (IM7, 103045); CD141/Thrombomodulin (M80, 344121); CD27 (LG.3A10, 124235); CD206/MMR (15-2, 321143); Folate Receptor β/FR-β (94b/FOLR2, 391707); CD45 (2D1, 368543); CD31 (WM59, 303137); CD11b (ICRF44, 301353); CD68 (Y1/82A, custom conjugate); CD38 (HIT2, 303541); CD144/VE-Cadherin (BV9, 348517); CD304/Neuropilin-1 (12C2, 354525); CD86 (IT2.2, 305443); CD279/PD-1 (EH12.2H7, 329955); CX3CR1 (K0124E1, 355709); CD56/NCAM (QA17A16, custom barcode); CD14 (63D3, custom barcode). Antibodies against CD107a (LAMP-1), CD314 (NKG2D), CD19, CD8a, CD21, IgG Fc, CD209 (DC-SIGN), EGFR, CD196 (CCR6), CD1c, CD309 (VEGFR2), CD127 (IL-7Rα), CD273 (B7-DC, PD-L2), CD226 (DNAM-1), CD278 (ICOS), CD119 (IFN-γ R α chain), CD274 (B7-H1, PD-L1), CD3, CD55, IgM were used at a dilution of 1:250 (0.2 ug per 100 uL staining reaction), whereas the remaining antibodies were used at a dilution of 1:50 (1 ug per 100 uL staining reaction).

Antibodies used for immunofluorescence microscopy studies include CD3 (1:100, Clone M-20, sc-1127, Santa Cruz Biotechnology), CD138 (1:50, PA5-32305, Thermo Fisher Scientific), and CD20 (1:50, Clone L26, GTX29475, GeneTex), CLIC5 (1:50, clone 1E6, SAB1402589, Sigma), CD68 (1:50, clone 514H12, CD68-L-CE, Leica) CD3 (1:25, clone LN10, CD3-565-L-CE, Leica), HLA-DR (1:200, clone EPR3692, ab92511, Abcam), CD34 (1:100, clone QBEnd/10, END-L-CE, Leica) and CD90 (1:200, clone D3V8A, 13801, Cell Signaling Technology). All antibodies are listed in the following format (dilution, clone, catalog number, company).

Antibodies used for sorting T cell subsets for the T cell functional assays include anti-CD4 APC (1:100, RPA-T4, 300537), anti-CD8A BV711 (1:100, RPA-T8, 301044), anti-CD3 APC-Cy7 (1:100, OKT3, 317342), anti-CD14 FITC (1:100, HCD14, 325604), anti-CD45RA BV605 (1:100, HI100, 304134), anti-CCR7 PE-Cy7 (1:100, G043H7, 353226) and anti-PD-1 BV421 (1:100, EH12.2H7, 329920), all from Biolegend. Antibodies used for sorting memory B cells for T cell functional assays include anti-CD19 PE (1:100, HIB19, 302208), anti-CD27 BV421 (1:100, M-T271, 356418), anti-CD3 FITC (1:100, OKT3, 317306) and anti-CD14 APC (1:100, HCD14, 325608) all from Biolegend. Antibodies used to identify B cell subsets at the conclusion of the T-B cell co-cultures include anti-CD3 FITC (1:100, OKT3, 317306), anti-CD20 BV605 (1:100, 2H7, 302334), anti-CD19 APC-Cy7 (1:100, HIB19, 302218), anti-CD27 PE-Cy7 (1:100, M-T271, 356412), anti-CD38 BV785 (1:100, HIT2, 303530), anti-CD11c PE (1:50, Bu15, 337206), and anti-CD21 PerCP-Cy5.5 (1:100, Bu32, 354908), all from Biolegend. Cytotoxicity assays used anti-CD3 antibodies (OKT3, 50 ug/mL, BioXcell) as well as Annexin V (Biolegend).

Validation

All antibodies are commercially available and validated for flow cytometry, microscopy, functional assays, or CITE-seq of human cells as stated in the manufacturer's product information, quoted below:
Beckman Coulter flow cytometry: Beckman Coulter tests each lot for consistent performance, as verified on the Certificate of Analysis that accompanies each antibody.
BioLegend flow cytometry: Each lot of this antibody is quality control tested by immunofluorescent staining with flow cytometric analysis.
BioLegend TotalSeq-A: All lots are tested by flow cytometry to make sure they stain the expected cell population and that oligos are attached to the antibodies. This process has been validated by comparison with a traditional two-step flow cytometry staining as shown.
Leica immunofluorescence: Leica performs extensive staining experiments using a diversity of human normal and abnormal tissues to validate their antibodies. The results of these staining QC experiments are described in each Product Detail sheet.
Santa Cruz Biotechnology immunofluorescence: M-20 is a polyclonal goat anti-human CD3 that has been cited in 56 publications dating back over 20 years.
Thermo Fisher Scientific immunofluorescence: PA5-32305 is a polyclonal rabbit anti-human CD138 antibody. It was purified with antigen affinity chromatography and validated by the vendor by staining of human tonsil tissue.
GeneTex immunofluorescence: To optimize the performance of our reagents, we employ various analytic validation strategies to ensure both consistent quality and specificity. These modalities are in line with guidelines described by the International Working Group on Antibody Validation (IWGAV) and have become fundamental components of our quality assurance process:
- KO/KD Validation
- Comparable Abs
- IP/MS Analysis
- Orthogonal Validation
- Protein Overexpression

Sigma immunofluorescence: Clone 1E6 is a mouse monoclonal antibody against GST-tagged human CLIC5. According to the vendor, it is specific for the immunogen by Western blot. According to Novus Bio, which sells the same clone, it is also specific for recombinant CLIC5 without the GST tag by ELISA and Western. The vendor has also performed validation staining of human placenta.
Abcam immunofluorescence: Antibody specificity is confirmed by looking at cells that either do or do not express the target protein

within the same tissue. Initially, our scientists will review the available literature to determine the best cell lines and tissues to use for validation. We then check the protein expression by IHC/ICC to see if it has the expected cellular localization. If the localization of the signal is as expected, this antibody will pass and is considered suitable for use in IHC/ICC. We use a variety of methods, including staining multi-normal human tissue microarrays (TMAs), multi-tumor human TMAs, and rat or mouse TMAs during antibody development. These high-throughput arrays allow us to check many tissues at the same time, providing uniformly as all tissues are exposed to the exact same conditions.
CST immunofluorescence: All CST™ antibodies that are approved for use in immunofluorescent assays have undergone a rigorous validation process. Validation steps include:
- Cell lines or tissues with known target expression levels are used to verify specificity.
- Appropriate cell lines and tissues are used to verify subcellular localization.
- Antibody performance is assessed on appropriate tissues.
- Cells are subjected to phosphatase treatment to verify phospho-specificity. Target specificity is also verified with the use of known knockout or null cell lines.
- Cells are subjected to siRNA treatment or over-expression of the target protein to verify target specificity.
- Activation state specification, target expression, and translocation are examined using ligands or inhibitors to modulate pathway activity.
- Requirement of threshold signal-to-noise ratio in antibody:isotype comparison and minimum fold-induction for phospho-specific antibodies ensures the greatest possible sensitivity.
- Fixation and permeabilization conditions are optimized; alternative protocols are recommended if necessary.
Stringent testing ensures lot-to-lot consistency.

Eukaryotic cell lines

Policy information about cell lines

Cell line source(s)	CD32-expressing murine fibroblast L cells were kindly gifted to Deepak Rao by Megan Levings (PMID: 36470208)
Authentication	The L cells were not recently authenticated.
Mycoplasma contamination	L cells were not tested for mycoplasma contamination.
Commonly misidentified lines (See ICLAC register)	No commonly misidentified cell lines were used in this study as L cells are not on the list of commonly misidentified cell lines.

Human research participants

Policy information about studies involving human research participants

Population characteristics	Male and female patients with rheumatoid arthritis according to the ACR 2020 Rheumatoid Arthritis classification criteria. The patients were recruited into three different cohorts: treatment-naive patients (n=28) early in their disease course (mean 2.64 years), methotrexate-inadequate (MTX) responders (n=27), and anti-TNF agent inadequate responders (n=15). The patients were similar in age, sex, disease activity, and other clinical parameters across the three treatment groups. In addition, nine patients with osteoarthritis were recruited. Additional population characteristics detailed in Supplementary Table 1.
Recruitment	Participants were recruited by physician referral from 13 clinical sites across the United States and 2 sites in the United Kingdom. Only patients with active disease were recruited. Recruitment occurred mainly at academic medical centers, which may be more likely to see complex cases. Different sites used different techniques for joint biopsies, which may introduce bias. Recruitment site and biopsy method are addressed as potential confounders in the paper.
Ethics oversight	The study was performed in accordance with protocols approved by the Institutional Review Board at Stanford University (Protocol ID: 33561). All clinical and experimental sites obtained approval for this study from their Institutional Review Boards.

Note that full information on the approval of the study protocol must also be provided in the manuscript.

Flow Cytometry

Plots

Confirm that:

☒ The axis labels state the marker and fluorochrome used (e.g. CD4-FITC).

☒ The axis scales are clearly visible. Include numbers along axes only for bottom left plot of group (a 'group' is an analysis of identical markers).

☒ All plots are contour plots with outliers or pseudocolor plots.

☒ A numerical value for number of cells or percentage (with statistics) is provided.

Methodology

Sample preparation	Cryopreserved synovial tissue fragments were disaggregated, and live cells were obtained by cell sorting (CD235a- live-dead dye-). The first 60,000 cells were used for CITE-seq studies. The next 50,000 sorted live cells were used for flow cytometry.

Instrument	A BD FACSAria Fusion cell sorter was used for all sorting and analytic flow cytometry except for the outcome analytic flow cytometry of the T-B cell co-culture assay, which was performed on a BD Fortessa analyzer.
Software	Data were collected using FACSDiva software 8.0.1 and analysed using FlowJo v10.6.
Cell population abundance	During the initial sort of live cells for CITE-seq, live cells represented a mean of 59.2% and median of 61.6% of cells (S.D. 17.9%). Cell populations of interest in the other flow panels ranged from <1% to >50%, depending on the population and sample.
Gating strategy	Synovial cell populations were gated as follows: A very large FSC vs SSC gate designed to capture small lymphocytes as well as large fibroblasts and macrophages. After singlet gating, dead cells and red blood cells were gated out using fixable viability dye and anti-CD235a antibodies pooled into the same channel. Cell populations were identified as follows: CD45+CD3-CD14-CD19+ (B cells), CD45-CD31-CD146- (fibroblasts), CD45+CD3-CD14+ (myeloid), CD45+CD3+CD14- (T cells). Gating of these populations is shown in Supplementary Figure 1G.<br>For functional T cell assays, T cells were sorted from live cells (negative for LIVE/DEAD Fixable Aqua Dead Cell Stain) as follows: CD14-CD3+CD4+CD8-CD45RA-PD-1hi (TPH+TFH), CD14-CD3+CD4+CD8-CD45RA-PD-1- (PD-1- Memory CD4), CD14-CD3+CD4-CD8+CD45RA- (Memory CD8), CD14-CD3+CD4-CD8+CD45RA+CCR7- (TEMRA CD8). For these assays, memory B cells were sorted from live cells (negative for LIVE/DEAD Fixable Aqua Dead Cell Stain) as follows: CD19+CD27+CD3-CD14-. At the conclusion of the T-B co-culture, B cell subsets were identified as follows: CD27hi CD38hi CD19+ (plasmablasts) and CD11c+ CD21- CD19+ (ABCs). In the cytotoxicity assay, dead cells were identified as Annexin V+. Representative gating of the conclusion of the T-B co-culture experiment is shown in Figure 3d.

☒ Tick this box to confirm that a figure exemplifying the gating strategy is provided in the Supplementary Information.
