# Supplementary material for: Deconstruction of rheumatoid arthritis synovium defines inflammatory subtypes
Source: Nature. 2023 Nov 8;623(7987):616–24. doi: 10.1038/s41586-023-06708-y (PMC10651487; doi:10.1038/s41586-023-06708-y)
Supplement: Supplementary file 3 — Supplementary Figure 4 [file 41586_2023_6708_MOESM3_ESM.pdf]

Supplementary Figure 4a-b

a

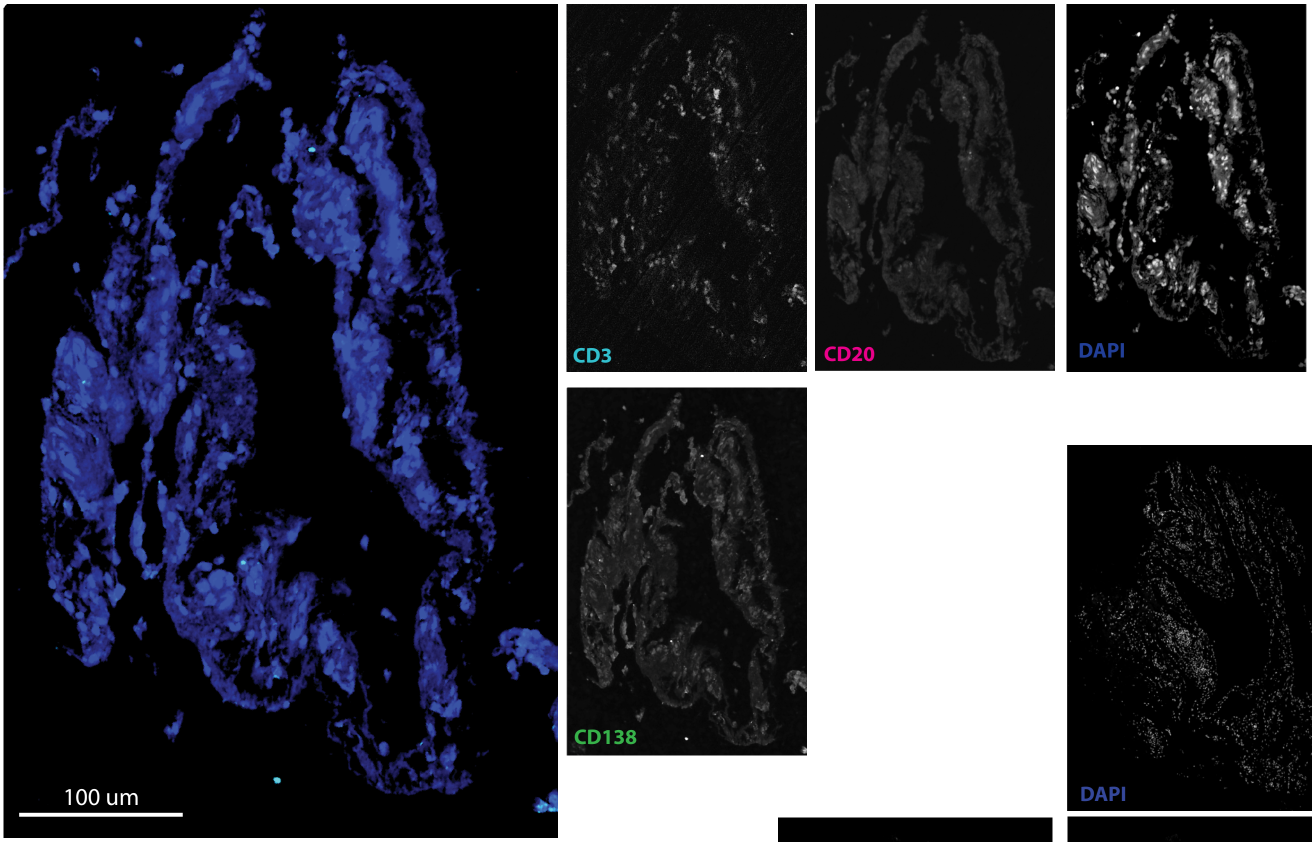

b

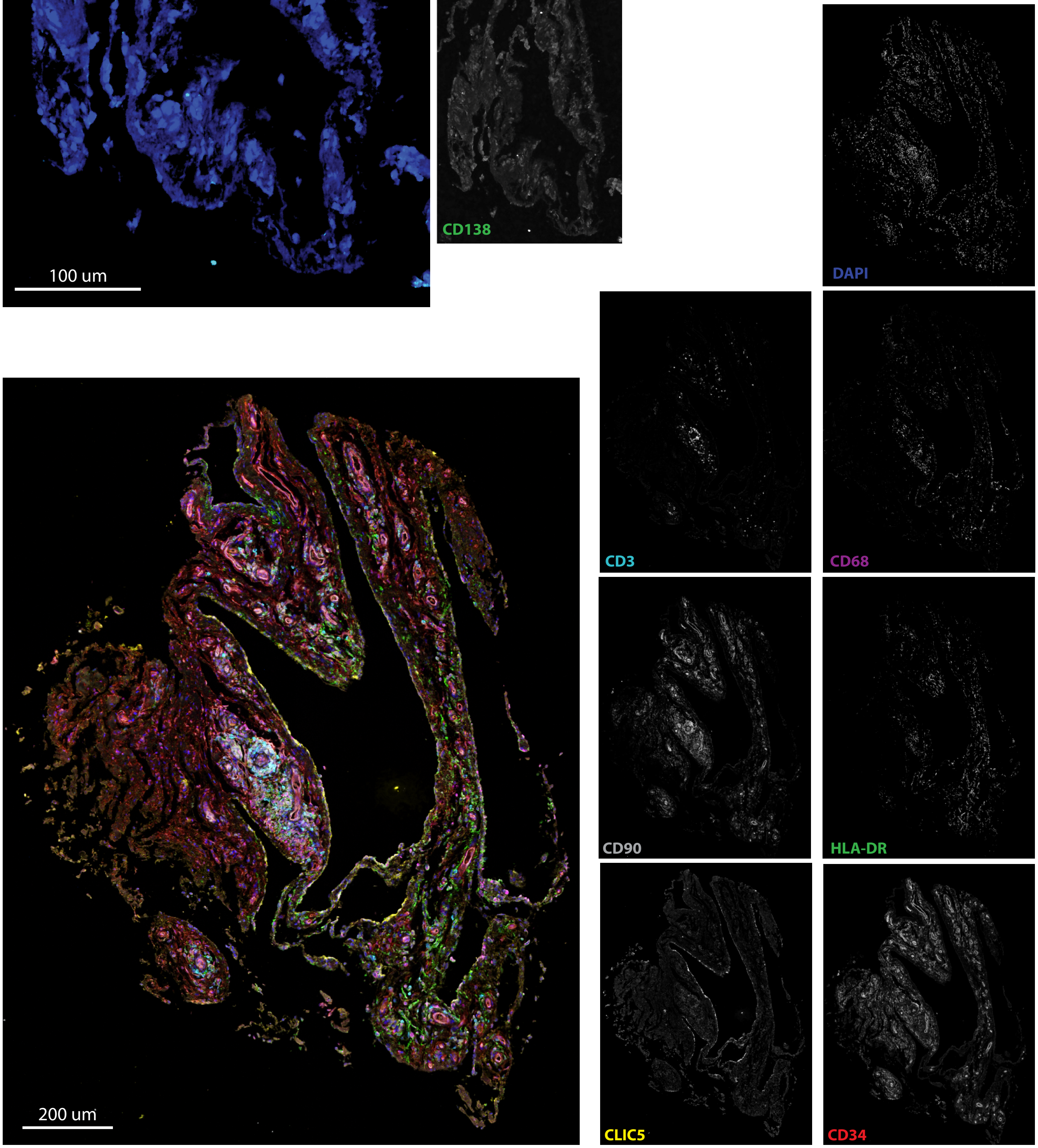

Supplementary Figure 4c-d

c

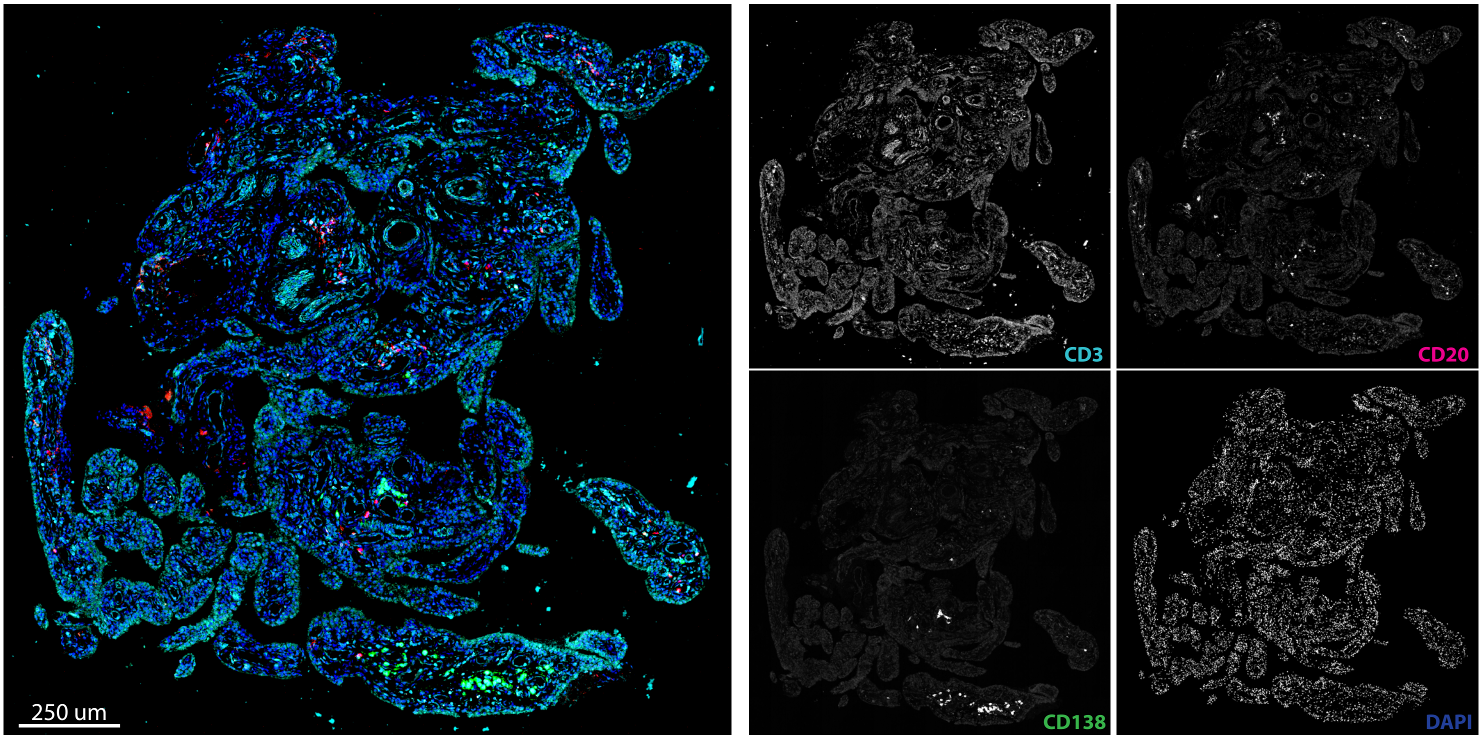

d

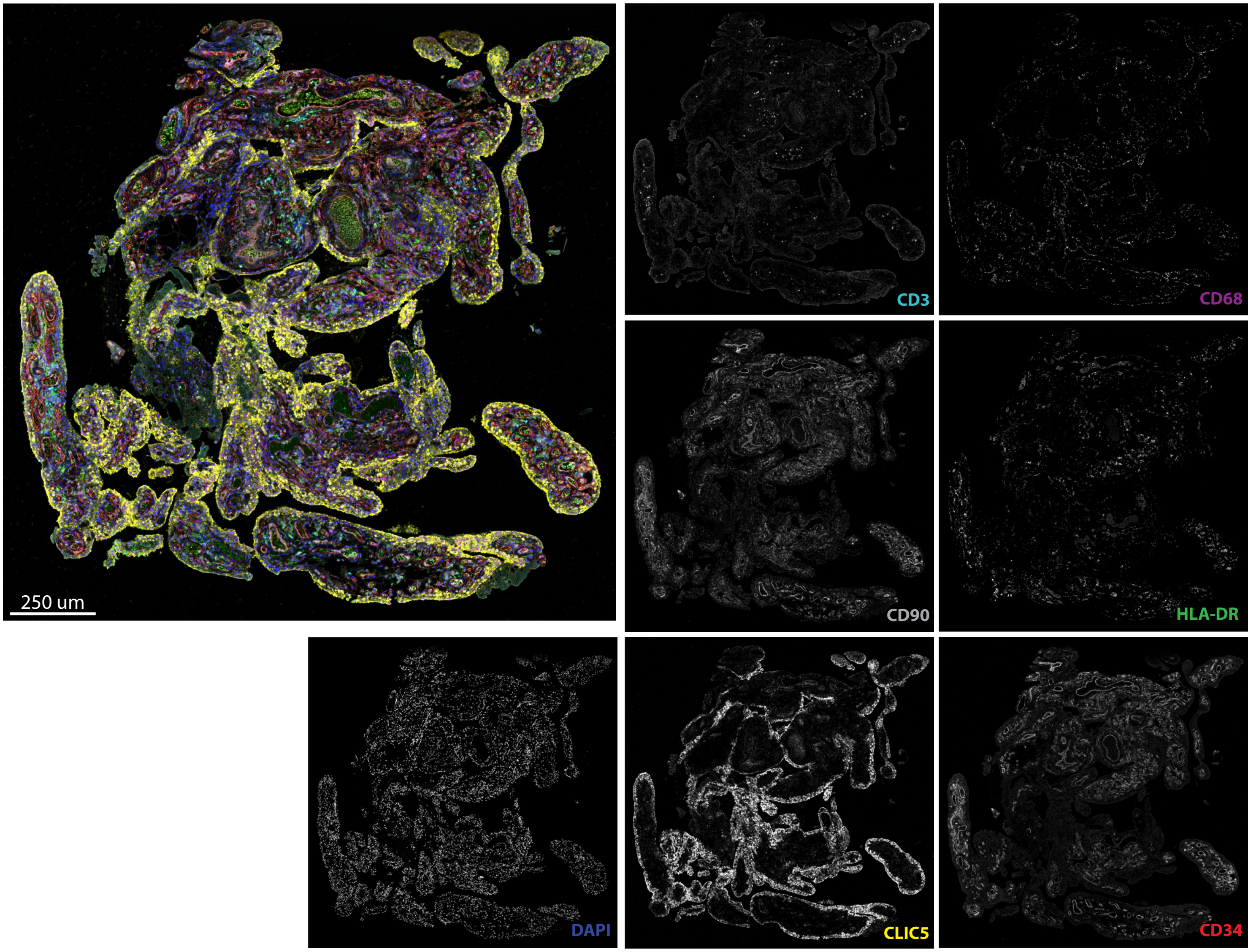

e

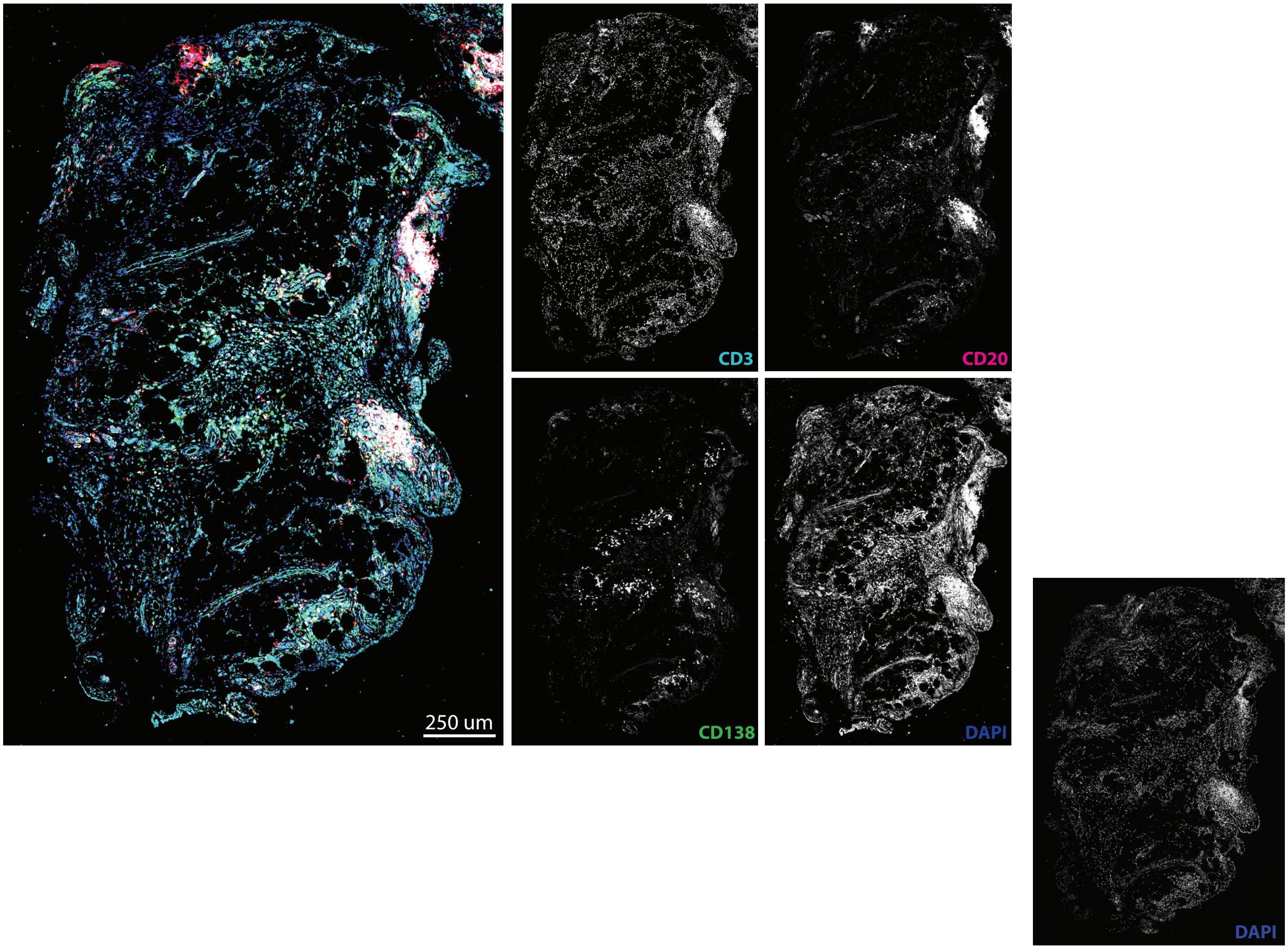
250 um
CD3
CD20
CD138
DAPI
DAPI

f

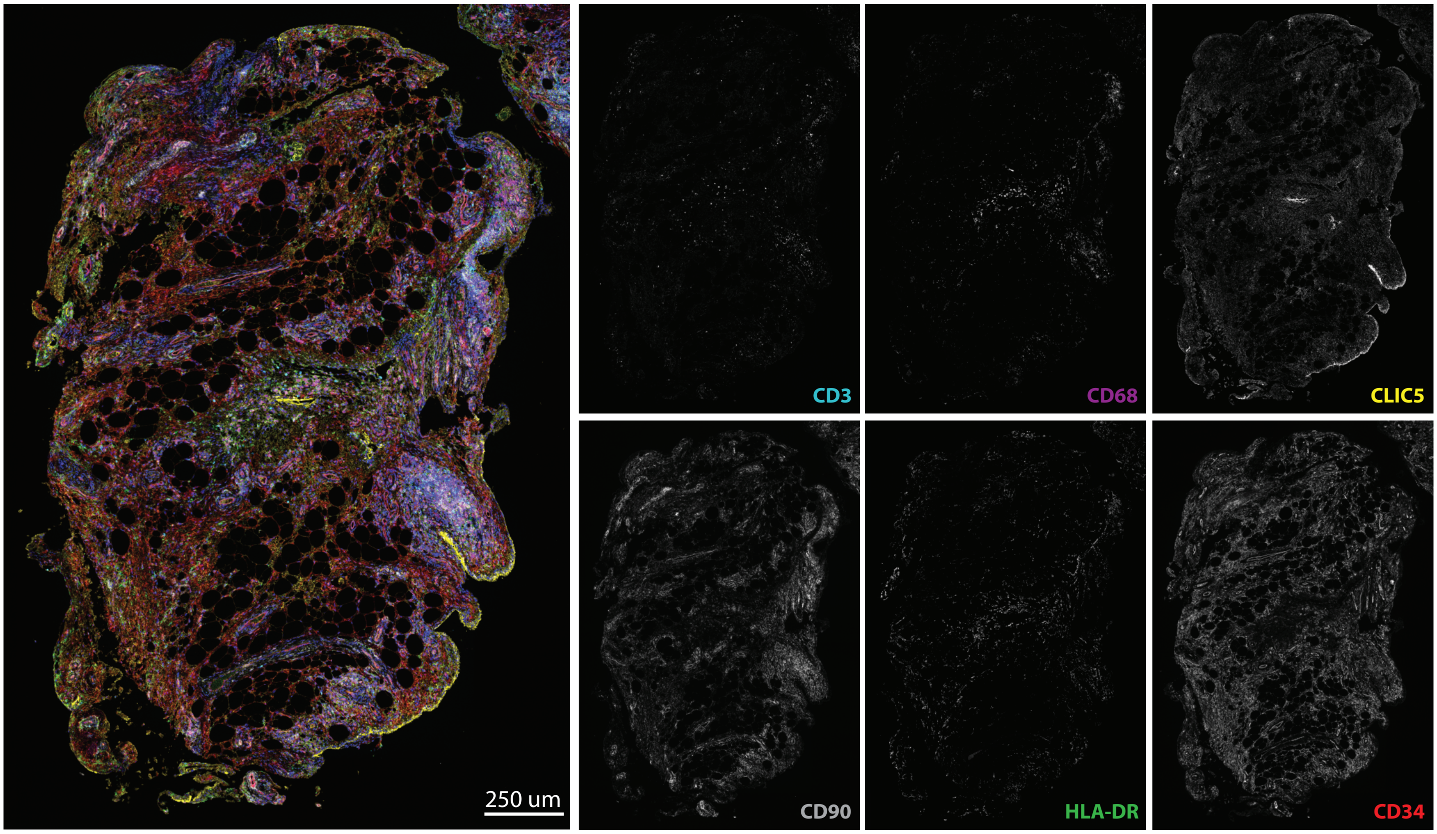
250 um
CD3
CD68
CLIC5
CD90
HLA-DR
CD34

g

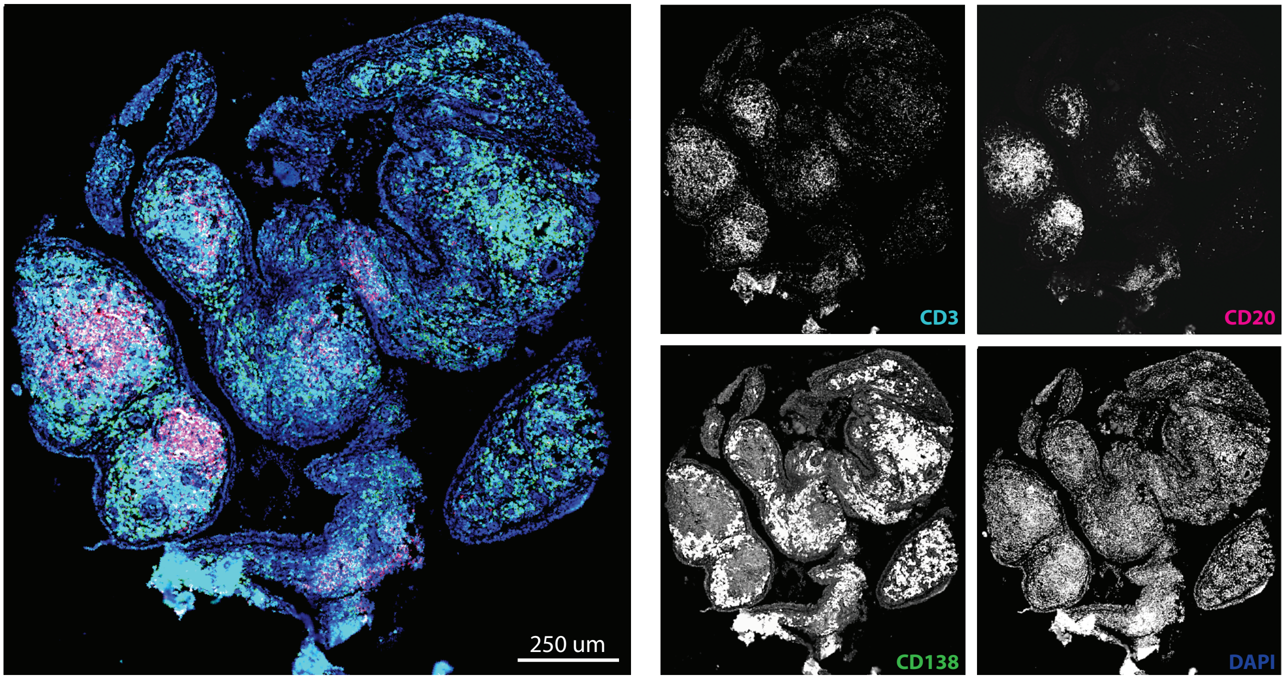
250 um
CD3
CD20
CD138
DAPI

h

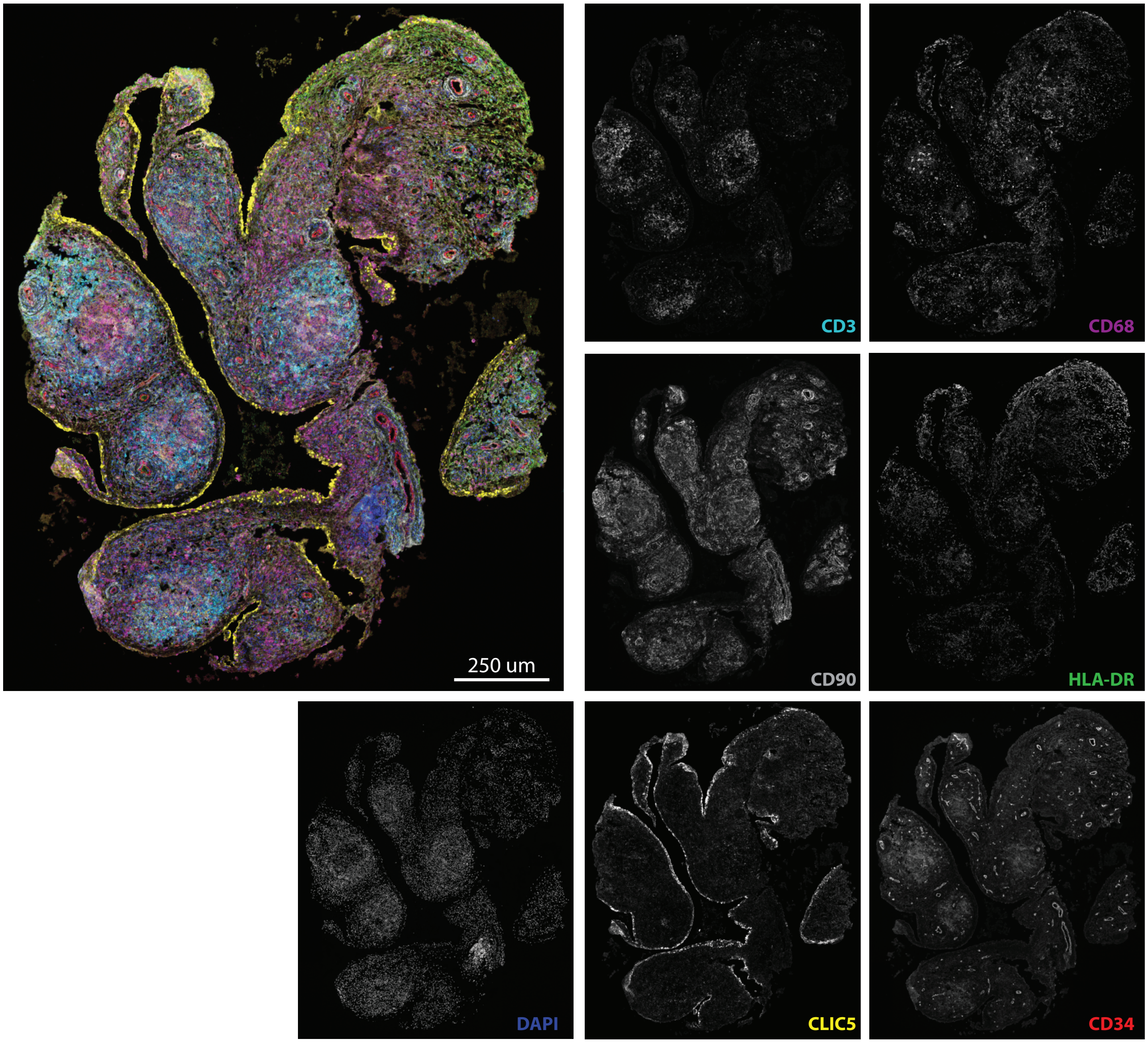
250 um
CD3
CD68
CD90
HLA-DR
DAPI
CLIC5
CD34

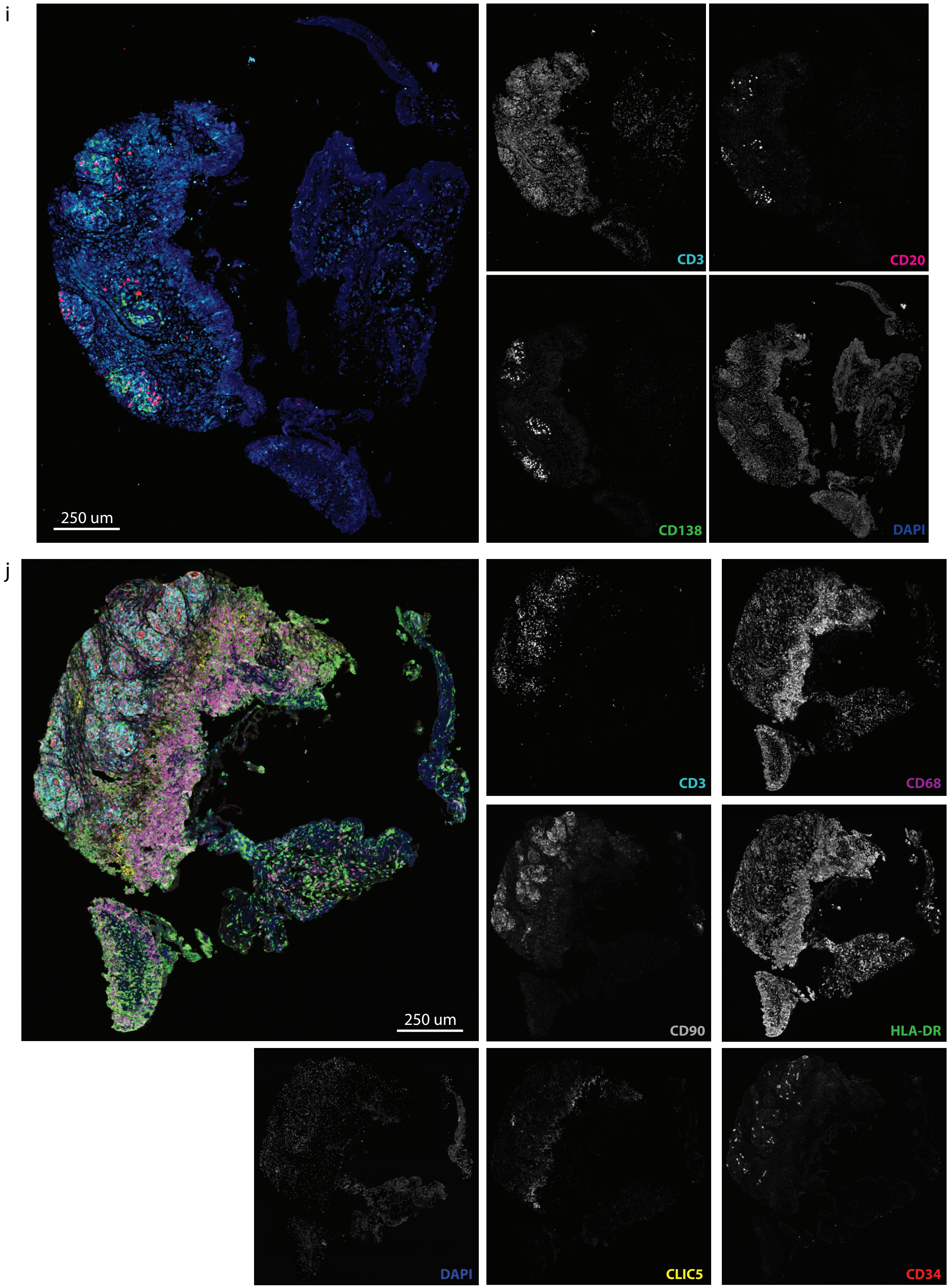
i
250 um
CD3
CD20
CD138
DAPI
j
250 um
CD3
CD68
CD90
HLA-DR
DAPI
CLIC5
CD34

Supplemental Figure 4k-l

k

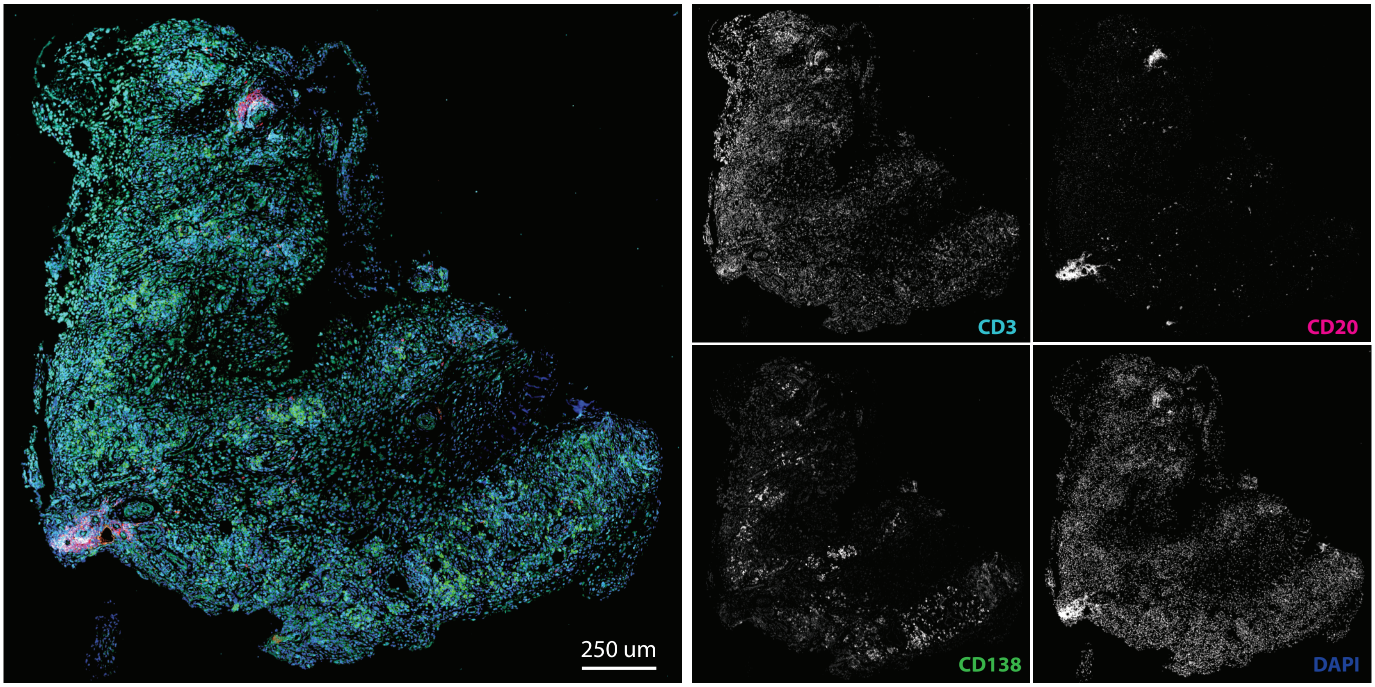

l

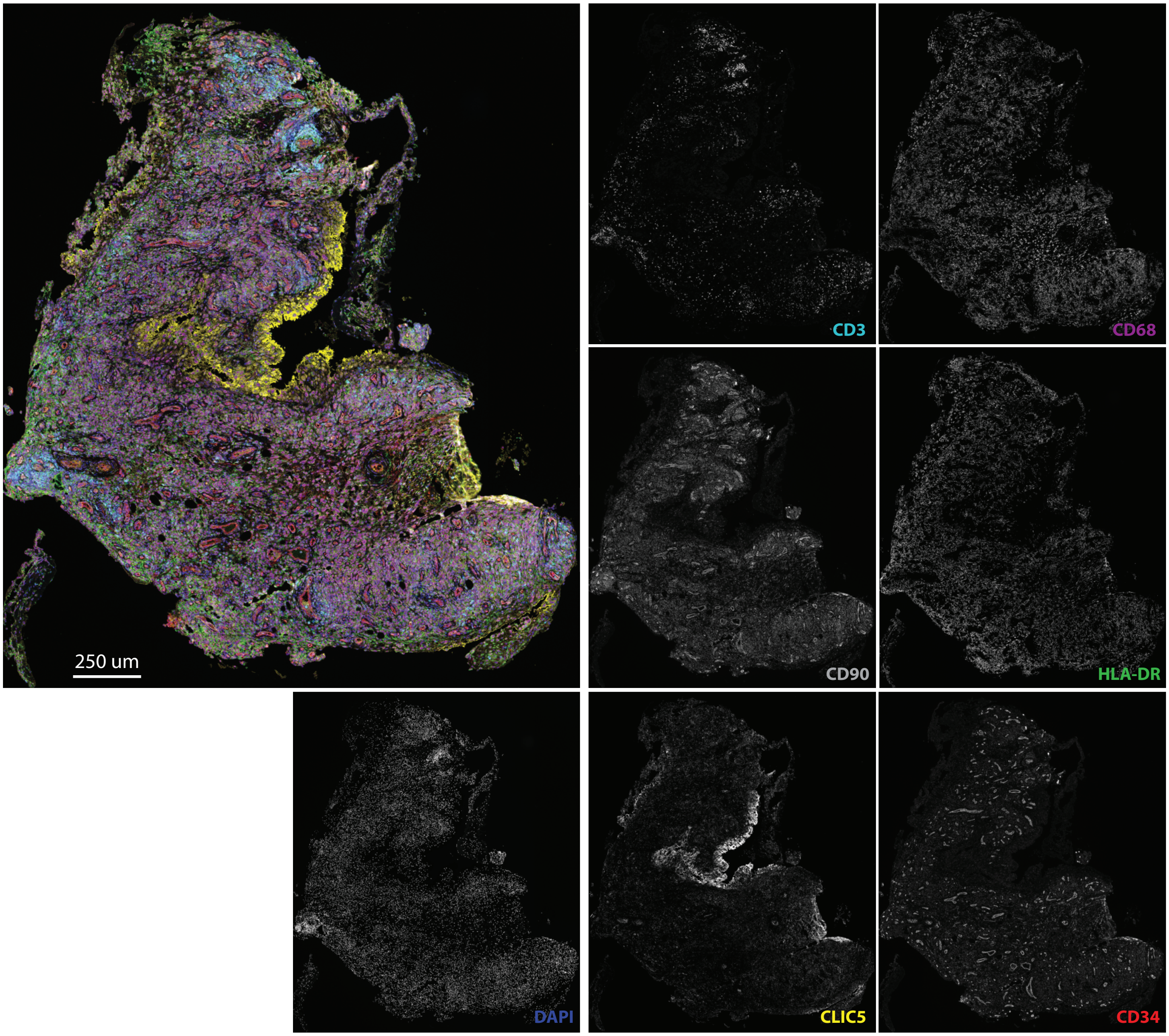

Supplementary Fig. 4. Representative histology images for each CTAP. Representative fragments from patients in each CTAP, showing composite and individual staining of each marker in the lymphocyte panel (**a, c, e, g, i, k**) or stromal cell panel (**b, d, f, h, j, l**). A total of 150 fragments from 36 individuals (mean 4.2 fragments per individual, range 2-9 fragments per individual) were stained in batches and analyzed as a single cohort. A high-resolution version of this figure is available at https://doi.org/10.5281/zenodo.8364277.
